# Reversible interconversion and maintenance of mammary epithelial cell characteristics by the ligand-regulated EGFR system

**DOI:** 10.1038/srep20209

**Published:** 2016-02-02

**Authors:** Shinji Fukuda, Hisayo Nishida-Fukuda, Daisuke Nanba, Koh-ichi Nakashiro, Hironao Nakayama, Hiroyuki Kubota, Shigeki Higashiyama

**Affiliations:** 1 Division of Cell Growth and Tumor Regulation, Proteo-Science Center (PROS), Ehime University, Toon, Ehime, Japan; 2 Department of Biochemistry and Molecular Genetics, Ehime University Graduate School of Medicine, Toon, Ehime, Japan; 3 Department of Oral and Maxillofacial Surgery, Ehime University Graduate School of Medicine, Toon, Ehime, Japan; 4 Division of Integrated Omics, Research Center for Transomics Medicine, Medical Institute of Bioregulation, Kyushu University, Fukuoka, Japan; 5 PRESTO, Japan Science and Technology Corporation, Fukuoka, Japan

## Abstract

Epithelial cell plasticity is controlled by extracellular cues, but the underlying mechanisms remain to be fully understood. Epidermal growth factor (EGF) and amphiregulin (AREG) are high- and low-affinity ligands for EGF receptor (EGFR), respectively. EGFR signaling is known to promote epithelial-mesenchymal transition (EMT) by the activation of ERK and the induction of an EMT transcription factor, ZEB1. Here, we demonstrate that ligand-switching between EGF and AREG at equivalent molarity reversibly interconverts epithelial and mesenchymal-like states of EGFR signal-dependent mammary epithelial cells. The EGF- and AREG-cultured cells also differ in their epithelial characteristics, including the expression of cell surface markers, the mode of migration and the ability for acinus-formation. The ligand-switching between EGF and AREG temporally alters strength of the shared EGFR-ERK signaling. This alteration inverts relative expression levels of ZEB1 and its antagonizing microRNAs, *miR-205* and *miR-200c*, those are critical determinants of the epithelial phenotype. Further, AREG-induced EGFR accumulation on the plasma membrane compensates for the weak association between AREG and EGFR. The EGFR dynamics enables AREG to support proliferation as efficiently as EGF at equivalent molarity and to maintain epithelial characteristics. Our findings reveal a role of EGFR ligands-generated signal strength in the regulation of mammary epithelial cell plasticity.

Epithelial and mesenchymal cell characteristics are regulated by the epithelial-mesenchymal transition (EMT) and the mesenchymal-epithelial transition (MET) programs^1^,^2^. During the development, successive rounds of EMT and MET are required for the generation of many tissues/organs, such as mammary gland, liver, kidney and heart^1^. In animal tumor models, EMT and the subsequent MET have been shown to play critical roles in the metastatic process^3^,^4^. These findings illustrate the fundamental importance of epithelial cell plasticity regulated by the repeated EMT/MET cycles.

Numerous growth factors and cytokines act as the EMT inducers^1^. In response to these extracellular cues, EMT transcriptions factors, including ZEB, SNAIL, SLUG and TWIST, act as repressors of epithelial genes, such as *E-cadherin*. As the post-translational mechanism, microRNAs (miRNAs) that suppress the expression of EMT transcription factors participate in EMT. Transforming growth factor β (TGFβ) superfamily are well-known EMT inducers^5^. In response to TGFβ stimulation, ZEB1 suppresses the expression of its antagonizing miRNAs, *miR-200* family and *miR-205*, and promotes EMT^6^,^7^. Further, the removal of TGFβ1 triggers the inversion of the relative expression levels of ZEB1 and its antagonizing miRNAs, by which mesenchymal state can be reversed to epithelial state^8^. These results suggest that the negative feedback loop involving EMT transcription factors and miRNAs establishes the EMT/MET regulatory signaling network, and it could act as the molecular switch to determine the epithelial or mesenchymal phenotypes^9^.

Epidermal growth factor (EGF) is also known as the inducer of EMT. In humans, there are 13 ligands in the EGF family^10^,^11^. In certain normal cells and at least some pre-malignant cells, EGF family members act as indispensable ligands for activation of the EGFR and subsequent cell growth^11^. EGF and amphiregulin (AREG) are structurally conserved cognate growth factors that bind to EGFR. Knockout mouse studies showed not only a functional redundancy between EGF and AREG, but also a putative unique role of AREG in mammary glands development^12^,^13^. Biochemical analysis revealed that the affinity of AREG for EGFR is 10-fold lower than that of EGF^14^. Because of the difference in their affinities to EGFR, EGF induces strong activation of EGFR and the downstream molecule, ERK, whereas the extent of AREG-induced EGFR-ERK activation is comparatively weak. Previous studies reported that Raf1-estrogen receptor fusion protein or oncogenic Ras-V12 regulate EMT and cell motility through ZEB1^15^,^16^; however, it still remains elusive whether the signal strength generated by the different EGFR ligands and their concentrations affect epithelial cell plasticity.

In a series of reports, we have characterized EGFR ligands, emphasizing juxtacrine and paracrine signaling, ectodomain shedding, intracellular trafficking and mRNA stability^17^,^18^,^19^,^20^,^21^,^22^. Here, we used EGF and AREG as the representatives of cognate growth factors, and set to understand their mechanism of action using immortalized mammary epithelial cell lines, MCF10A and HMT-3522 S1. These cells show complete dependence on EGFR signaling for cell growth, but not other RTK signaling, allowing for the precise and direct evaluation of the properties of EGFR ligands as the cellular phenotypes.

## Results

### EGF and AREG ligand-switching induced reversible phenotypic changes in MCF10A

**Figure 1:**
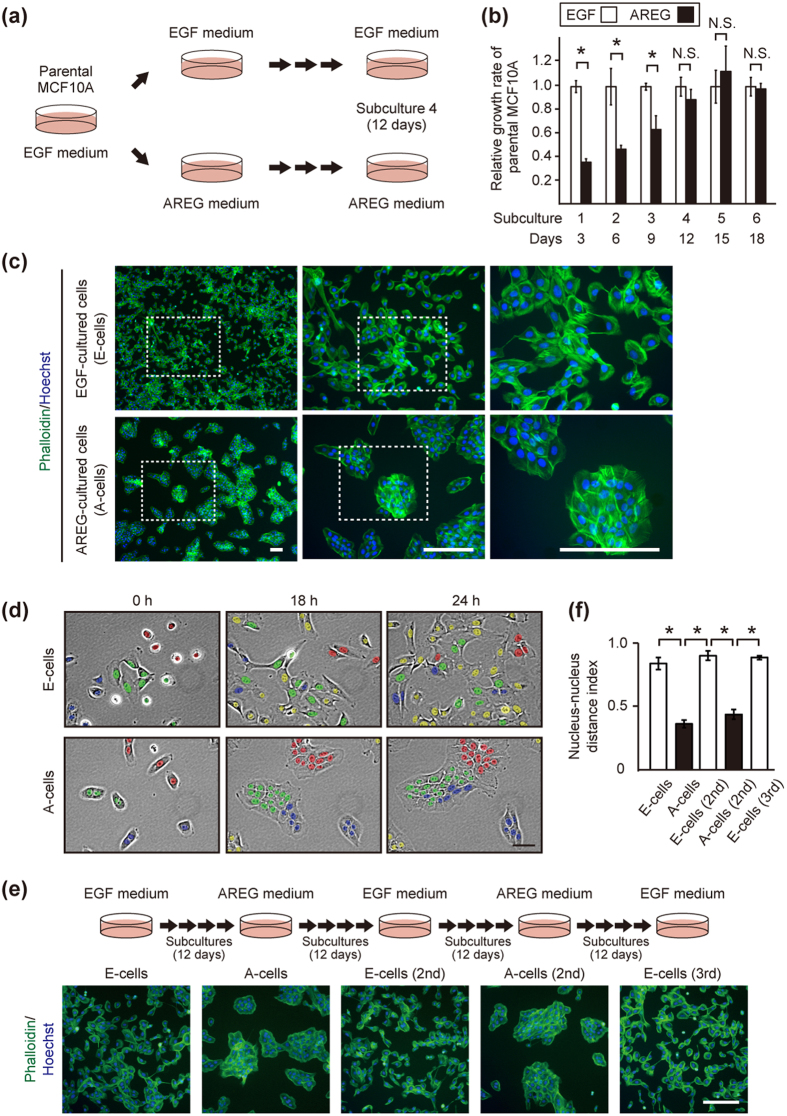
EGF and AREG reversibly generated distinct phenotypes of MCF10A. (**a**) Illustrations of the culture systems. Parental MCF10A cells were cultured in parallel in the presence of EGF (10 ng/mL [1.8 nM]) or AREG (20 ng/mL [1.8 nM]). “Subculture” denotes the trypsinization and replating of the cells in new culture dishes every 3 days. For further details of cell culture, see Supplementary Fig. 1b. (**b**) Relative growth rates of parental MCF10A cells cultured in the presence of EGF or AREG. The values of fold-increase in the number of A-cells were expressed relative to that of E-cells, which was assigned a value of 1. *denotes p < 0.05; N.S. denotes not significant. See Supplementary Fig. 1c. (**c**) Fluorescent phalloidin- and Hoechst-staining images of E-cells (upper panels) and A-cells (lower panels) of the 4th subculture on the 3rd day after plating. Boxed areas are shown to the right at a higher magnification. Scale bar: 100 μm. (**d**) Representative time-lapse images of E-cells and A-cells (4th subculture). Image acquisition was started the day after plating. Cells observed at the starting point of image acquisition (0 h) were labeled with red, green and blue according to cluster location. During observation, some cells appeared from outside of the field, and they were labeled with yellow. Initial cluster labeling became obscure over time in E-cells, whereas the clusters were retained in A-cells. See Supplementary Fig. 1d and Supplementary Movies 1a and 1b. Scale bar: 50 μm. (**e**) Upper panels illustrate repeated ligand-switching. For further details of cell culture, see Supplementary Fig. 1b. Lower panels show fluorescent phalloidin- and Hoechst-staining images of the sequentially generated E-cells and A-cells on the 3rd day after plating. Cells were subcultured at least 4 times after each ligand-switch. Scale bar: 50 μm. (**f**) Quantification of cell clustering. Nucleus-nucleus distance index is close to 1.0 when cells are sparsely distributed while it is close to 0 as cells become clustered. *p < 0.05. For further details, see Supplementary Fig. 1e and Methods.

We utilized MCF10A cells, for which exogenous EGF is essential for proliferation^23^ (Supplementary Fig. 1a). MCF10A cells were subcultured in parallel in either conventional EGF medium or in medium containing AREG at equivalent molarity (Fig. 1a See also Supplementary Fig. 1b for further details of cell culture). In the first through third subcultures, the growth rate of AREG-cultured cells was significantly lower than that of EGF-cultured cells (Fig. 1b and Supplementary Fig. 1c). This effect was consistent with a previous report showing that AREG acted as a much weaker growth stimulator than EGF^14^. Unexpectedly, however, the growth rate of AREG-cultured cells gradually increased. After the fourth subculture, there was no statistical difference between the growth rates of the two populations (Fig. 1b and Supplementary Fig. 1c). The results indicated that AREG was able to support cell proliferation as efficiently as EGF after several subcultures.

Of note, the AREG-cultured cells looked like islands and their edges became smooth, showing characteristics typical of epithelial cells (Fig. 1c, lower panels). In contrast, EGF-cultured cells showed a mesenchymal cell-like appearance (Fig. 1c, upper panels). Although MCF10A cells were originally established as luminal ductal cells^24^, previous reports showed that EGF-cultured MCF10A underwent spontaneous morphologic changes into a fibroblast-like shape in sparse culture conditions^25^,^26^. In this study, after the ligand-switching (subculture 1), we further repeated the subculture at least 3 times, that is, cells were kept for at least 12 days under the individual conditions. We designated the EGF-cultured mesenchymal-like cells as “E-cells” and the AREG-cultured epithelial-like cells as “A-cells” (Fig. 1c).

Live imaging revealed that E-cells migrated in a random manner almost independent of their neighboring cells, whereas A-cells did not separate from one another after division (Fig. 1d Supplementary Fig. 1d and Supplementary Movies 1a and 1b). It should be noted that when A-cells were detached and subcultured in the EGF medium again, intercellular contacts were markedly decreased and cells re-acquired morphological characteristics of the parental MCF10A (Fig. 1e). Moreover, by repeating ligand-switching between EGF and AREG, parental MCF10A underwent at least 4 cycles of morphological change, yielding a third generation of E-cells (Fig. 1e,f and Supplementary Fig. 1e See also Supplementary Fig. 1b for further details of cell culture). These results demonstrated that EGF and AREG at equivalent molarity induced different characteristics of MCF10A in a reversible manner.

### EGF and AREG regulated mesenchymal and epithelial gene programs

**Figure 2:**
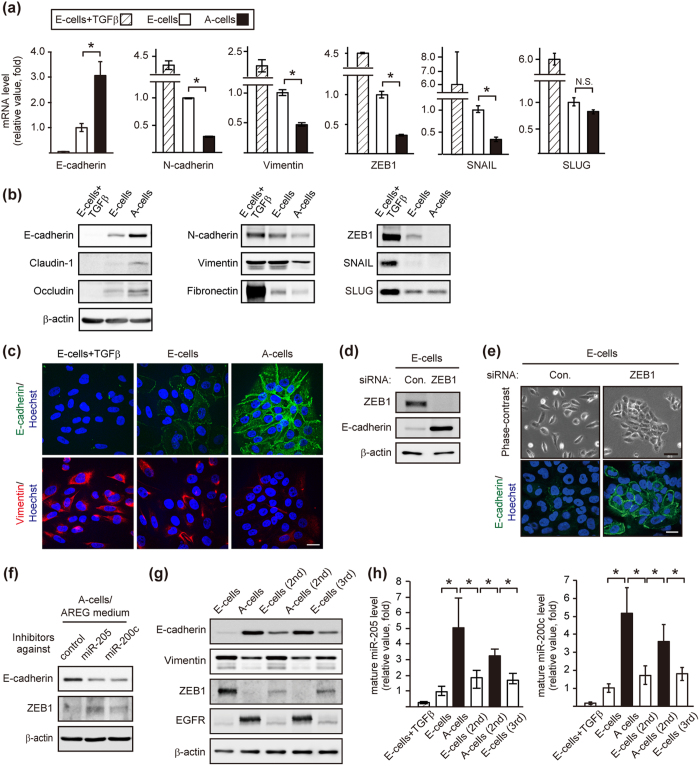
Ligand-switching reversibly regulated mesenchymal and epithelial gene programs via ZEB1. (**a**) RT-qPCR analysis of the expression of EMT-related factors. The mRNA levels in A-cells were expressed relative to that of E-cells. *p < 0.05. E-cells were treated with TGFβ (10 ng/mL) for 12 days and used as a positive control. (**b**) Western blot analysis of EMT-related factors in E-cells, A-cells and TGFβ-treated E-cells. (**c**) Immunofluorescent images of E-cells, A-cells and TGFβ-treated E-cells stained with anti-E-cadherin (green) and vimentin (red) antibodies. Nuclei were stained with Hoechst 33342 (blue). Scale bar: 25 μm. (**d**) Western blot analysis of E-cadherin in E-cells transfected with a control or *ZEB1* siRNA. (**e**) Phase contrast and immunofluorescent images of E-cells transfected with a control or *ZEB1* siRNA. Cells were stained with an E-cadherin (green) antibody. Nuclei were stained with Hoechst 33342 (blue). Scale bar: upper panel, 50 μm; lower panel, 20 μm. (**f**) Western blot analysis of E-cadherin and ZEB1 in A-cells transfected with inhibitors against *miR-205* or *miR-200c*. (**g**) Western blot analysis of EMT-related factors and EGFR in sequentially generated E-cells and A-cells. (**h**) RT-qPCR analysis of mature *miR-205* and *miR-200c* expression levels in sequentially generated E-cells and A-cells. TGFβ-treated E-cells were used as control. The miRNA levels in A-cells, E-cells (2nd), A-cells (2nd) and E-cells (3rd) were expressed relative to that of E-cells. *p < 0.05.

Microarray analysis revealed that the relative expression levels of epithelial markers, E-cadherin, occludin and claudin, were higher in A-cells than E-cells. By contrast, E-cells showed higher expression levels of mesenchymal markers, N-cadherin, vimentin and fibronectin-1, than A-cells (Supplementary Table 1). Such features were reminiscent of EMT^1^,^2^. Since it is important to precisely interpret the degree of EGF/AREG-induced epithelial/mesenchymal alteration against a positive control, E-cells were cultured in the presence of recombinant TGFβ and EGF for 12 days^27^, and used as a positive control that displayed strong mesenchymal phenotype. We confirmed that representative mesenchymal and epithelial markers were expressed at higher and lower levels in E-cells than A-cells, respectively (Fig. 2a–c).

The microarray analysis also showed a higher expression of *ZEB1* and *ZEB2,* well-known EMT transcription factors, in E-cells than A-cells (Supplementary Table 1). Among key EMT transcription factors, the expression of ZEB1 was significantly higher in E-cells than A-cells (Fig. 2a,b and Supplementary Fig. 2a). Knockdown of *ZEB1* alone in E-cells was sufficient to induce E-cadherin expression in the EGF medium (Fig. 2d,e). Further, E-cadherin promoter activity^28^ was significantly higher in A-cells than E-cells, which was suppressed by ZEB1 overexpression (Supplementary Fig. 2b). As a reciprocal pattern to ZEB1, the expression of the *MIR205* host gene, a precursor of *microRNA (miR)-205*, was higher in A-cells than E-cells (Supplementary Table 1). It has been established that *miR-205/miR-200* and ZEB1 reciprocally suppress each other’s expression, and this double-negative feedback loop between ZEB1 and the *miR-200* family regulates EMT^7^. Among 4 mature miRNAs (*miR-205, 200a, 200b* and *200c*), *miR-205* and *miR-200c* appeared to be the major miRNAs expressed in A-cells, as judged by the threshold cycle (Ct value) in the quantitative reverse transcription polymerase chain reaction (RT-qPCR, Supplementary Fig. 2c). Indeed, transfection of oligonucleotide inhibitors against *miR-205* or *miR-200c* partially, but reproducibly, increased and decreased ZEB1 and E-cadherin expression in A-cells, respectively (Fig. 2f). Taken together, these results indicated that reciprocal expression of ZEB1 and *miR-205/200c* contributed to the phenotypic change.

We observed that the expression of the epithelial and mesenchymal markers were gradually increased and decreased, respectively, after the ligand-switching from EGF to AREG (Supplementary Fig. 2d,e). In the sequentially converted cells shown in Fig. 1e, the expression levels of ZEB1 and Vimentin were consistently higher in E-cells than A-cells, whereas those of E-cadherin, *miR-205* and *miR-200c* were consistently lower in E-cells than A-cells (Fig. 2g,h). These results suggested that the observed phenotypic change was associated with the alteration of EMT marker expressions. Further, the changes in EMT marker expressions were also observed in the 4 independent clones established by limiting dilution (Supplementary Fig. 2f,g). These results suggest that the process of phenotypic change involved at least cell conversion, and cannot simply be explained by the expansion of a specific subpopulation.

On the other hand, E cells (2nd and 3rd) displayed slightly higher E-cadherin expression and the lower ZEB1 expression than the original E cells (Fig. 2g and Supplementary Fig. 2g). We thus examined whether E-cells (2nd and 3rd) maintained for more passages become more closely resemble the original E-cells. We found that there was no significant difference in the expression of E-cadherin and ZEB1 between the early- and the late-passage populations (Supplementary Fig. 2h). These results suggest that an additional factor that acts together with EGF might be necessary for the full-reversion from the E-cells (2nd and 3rd) to the original E-cells’ characteristics.

### EGF and AREG reversibly interconverted distinct characteristics of mammary epithelial cells

**Figure 3:**
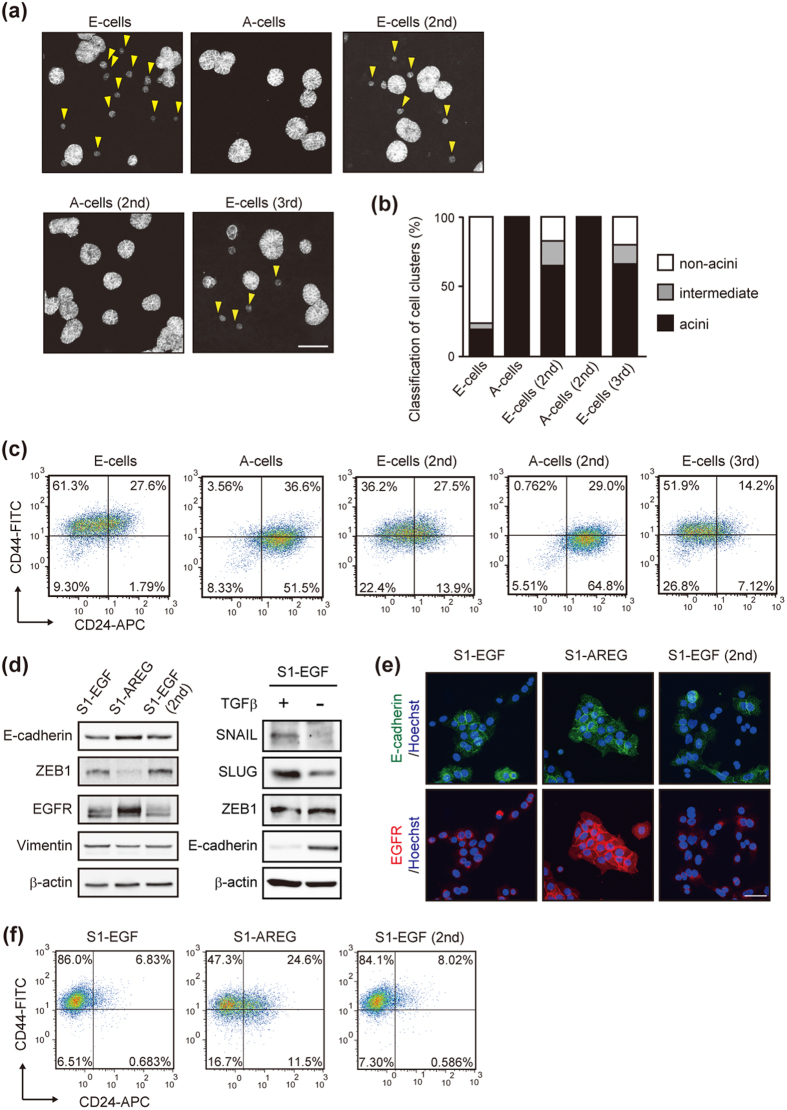
Ligand-switching reversibly interconverted distinct characteristics of mammary epithelial cells. (**a**) Representative projected Z-stack images of the acinus-like structures formed by E-cells and A-cells after a 2-week culture on the reconstituted basement membrane. Cells were visualized by fluorescent phalloidin staining. Yellow arrowheads indicate cell clusters judged as non-acinus. Scale bar: 100 μm. (**b**) Quantification of acinus-formation efficiency of sequentially generated E-cells and A-cells. The criteria for evaluating the acinus size are shown in Supplementary Fig. 3a. (**c**) Flow cytometric analysis of CD24 (X-axis) and CD44 (Y-axis) expression in sequentially generated E-cells and A-cells. (**d**) Western blot analysis of EMT related factors and EGFR in HMT-3522 S1 cells generated by the ligand-switching between EGF (10 ng/mL) and AREG (20 ng/mL). S1-EGF and S1-AREG indicate EGF- and AREG-cultured parental cells, respectively. S1-AREG cells were further cultured in the presence of EGF, generating S1-EGF (2nd). (**e**) Immunofluorescent images of S1-EGF, S1-AREG and S1-EGF (2nd) cells stained with anti-E-cadherin antibody (green) and anti-EGFR antibody (red). Nuclei were stained with Hoechst 33342 (blue). Scale bar: 40 μm. (**f**) FACS analysis of the expression of CD44 and CD24 in S1-EGF, S1-AREG and S1-EGF (2nd) cells.

We next assessed the character of E-cells and A-cells using a three-dimensional (3D) culture system. The 3D culture of MCF10A resulted in the formation of polarized acinus-like spheroids that recapitulate several aspects of glandular architecture *in vivo*^29^. In our experimental conditions, 30 to 50% of E-cells established a well-organized acinus-like structure. The remaining E-cells only formed cell clusters composed of 2 to 5 cells. In stark contrast, over 95% of A-cells established acinus-like structures (Fig. 3a,b and Supplementary Fig. 3a). The high efficiency with which acini were formed was also obtained when A-cells (2nd) were used, whereas E-cells (2nd and 3rd) showed significantly lower efficiency than A-cells (Fig. 3a,b).

Discrete subpopulations have been identified in mammary glands^30^,^31^,^32^ and in breast carcinomas^33^,^34^. Since the ligand-switching eventually affects the expression of ZEB1 that is implicated in breast cancer plasticity^35^, cells were characterized using antibodies against CD44 and CD24^33^,^34^. The main population of E-cells expressed a CD44^hi^/CD24^neg^ phenotype, whereas the phenotype of A-cells was CD44^lo^/CD24^pos^ (Fig. 3c). Purification of the 2 sub-populations, CD44^hi^/CD24^neg^ cells and CD44^lo^/CD24^pos^ cells in the E-cells, revealed that acinus-formation efficiency inversely correlated with the expression level of ZEB1, suggesting a role of ZEB1 in the EGF/AREG-induced determination of cell characteristics (Supplementary Fig. 3b). We also examined the expression of human mammary epithelial markers, CD49f (also known as integrin α6) and EpCAM^32^. The proportion of CD49^hi^/EpCAM^hi^ cells was consistently higher in A-cells than E-cells (Supplementary Fig. 3c). Judged by the acinus-formation efficiency^30^ and the expression of cell surface markers^36^, we speculated that A-cells were more similar to luminal progenitor cells in their characteristics than E-cells.

We also asked whether the EGF/AREG-induced phenotypic change could be observed in another human immortalized mammary epithelial cell line, HMT-3522 S1. This cell line also requires EGF for their cell proliferation^37^. In response to the ligand-switching from EGF to AREG, the expression of E-cadherin and ZEB1 was increased and decreased, respectively (Fig. 3d,e; S1-EGF vs. S1-AREG). After the switching from AREG to EGF, the reversal change occurred (Fig. 3d,e; S1-AREG vs. S1-EGF [2nd]). The expression of CD44 and CD24 was also reversibly regulated by the ligand-switching (Fig. 3f). These data indicated that ligand-switching resulted in a reversible change of the mammary epithelial cell characteristics, a change that we referred to as reversible interconversion.

### Both EGF and AREG drive mesenchymal characteristics but to different degrees

**Figure 4:**
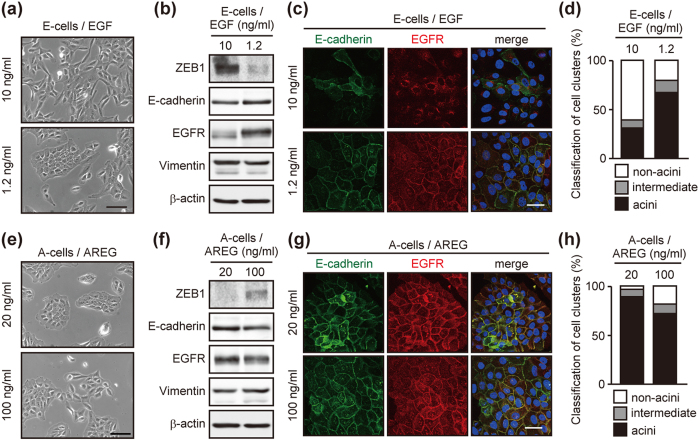
Dose-changing of EGF and AREG induces phenotypic conversion. (**a**) Phase-contrast images of cells cultured in the presence of different concentrations of EGF. Scale bar: 50 μm. (**b**) Western blot analysis of EMT-related factors and EGFR in cells cultured as in (**a**). (**c**) Immunofluorescent images of cells cultured as in (**a**). Cells were stained with antibodies against E-cadherin (green) and EGFR (red). Nuclei were stained with Hoechst 33342 (blue). Scale bar: 50 μm. (**d**) Quantification of acinus-formation efficiency. Cells shown in (**a**) were cultured for 2 weeks on a reconstituted basement membrane and their acinus size was evaluated. (**e**) Phase contrast images of cells cultured in the presence of different concentration of AREG. Scale bar: 50 μm. (**f**) Western blot analysis of EMT-related factors and EGFR expression in cells cultured as in (**e**). (**g**) Immunofluorescent images of cells cultured as in (**e**). Cells were stained as in (**c**). Scale bar: 50 μm. (**h**) Quantification of acinus-formation efficiency for cells shown in (**e**). Cells were processed as in (**d**).

Two possible mechanisms were considered to interpret the observed reversible interconversion: (1) EGF drives mesenchymal characteristics, whereas AREG drives epithelial characteristics, (2) both EGF and AREG drive mesenchymal characteristics but to different degrees. To distinguish the two possibilities, we changed the concentration of ligands. The low-dose EGF (1.2 ng/mL)-cultured cells established cell-cell contacts (Fig. 4a), and the expressions of E-cadherin and ZEB1 were upregulated and downregulated, respectively (Fig. 4b,c). These cells showed higher acinus-formation efficiency than the original E-cells (Fig. 4d and Supplementary Fig. 4a). In a complementary experiment, A-cells were cultured with a high-dose of AREG (100 ng/mL). A portion of A-cells lost cell-cell contact (Fig. 4e), and the expression of E-cadherin and ZEB1 was downregulated and upregulated, respectively (Fig. 4f,g). The high-dose AREG-cultured cells showed lower acinus-formation efficiency than the original A-cells (Fig. 4h and Supplementary Fig. 4a). These results suggest that both EGF and AREG drive mesenchymal characteristics, but the degree of AREG-induced mesenchymalization is much weaker than that induced by EGF at equivalent molarity. These experiments also revealed that the dose-changing as well as the ligand-switching trigger the changes in epithelial cell characteristics.

To further confirm the effect of AREG, E-cells were cultured in the absence of EGF for 24 h. Since cells no longer proliferated at this time points, we assumed that the AREG-specific effect would be observed in this condition. In the EGF-deprived cells, strong upregulation of ZEB1 was observed at 24 h and 48 h after the EGF stimulation. By contrast, AREG-induced ZEB1 upregulation was only weakly observed at 24 h, then decreased to the background level at 48 h (Supplementary Fig. 4b). These results suggest that both EGF and AREG upregulate the ZEB1 expression and drive mesenchymal characteristics, but to significantly different degrees.

### EGF and AREG directed reversible interconversion exclusively through EGFR

We next characterized receptors responsible for EGF/AREG signaling. To the best of our knowledge, the receptors for EGF/AREG are the EGFR homodimer and EGFR-ErbB2, EGFR-ErbB3 and EGFR-ErbB4 heterodimers^10^. Full inhibition of EGFR by AG1478 (high-dose: 1 μM) completely blocked cell growth (Supplementary Fig. 5a), indicating the indispensable role of EGFR for EGF/AREG. On the other hand, the knockdown of ErbB2 and/or ErbB3 had little effect on cell morphology and the E-cadherin expression (Supplementary Fig. 5b–d). The expression of ErbB4 was not detected in MCF10A (Supplementary Fig. 5e). These results suggested that EGF/AREG signals were exclusively mediated by EGFR, and the involvement of the other ErbB members was negligible at least in the interconversion.

**Figure 5:**
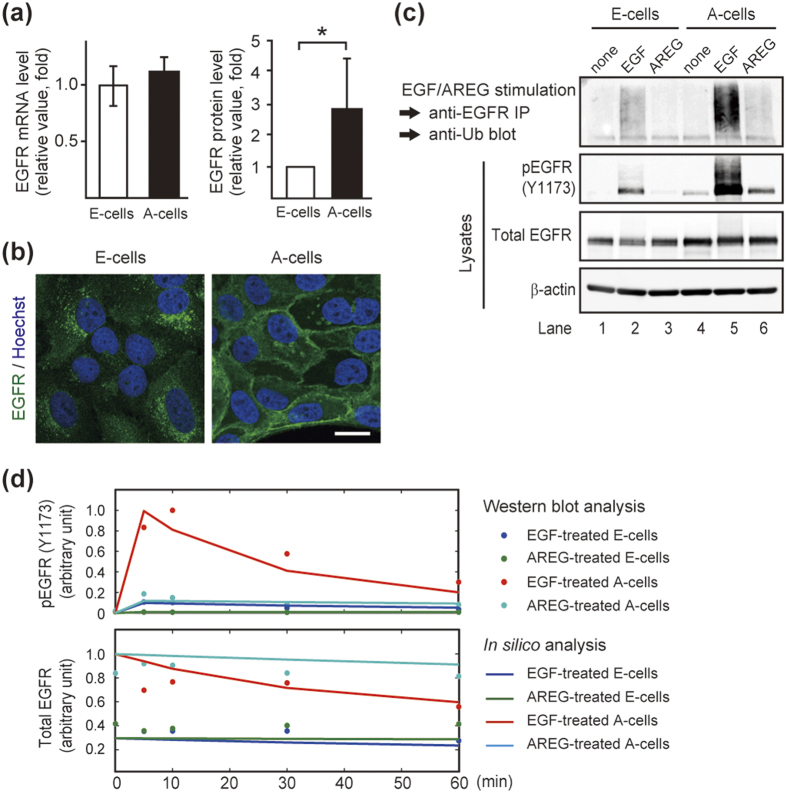
EGFR was responsible for EGF- and AREG-induced phenotypic conversion. (**a**) Quantification of *EGFR* mRNA by RT-qPCR (left) and EGFR protein in whole cell lysates by western blot analysis (right). The mRNA and protein levels of EGFR in A-cells were expressed relative to that of E-cells. *p < 0.05. (**b**) Immunofluorescent images of E-cells and A-cells stained with an anti-EGFR antibody (green). Nuclei were stained with Hoechst 33342 (blue). EGFR mainly localized in endosomes of E-cells and at the cell surface of A-cells. Scale bar: 20 μm. (**c**) EGF- and AREG-induced ubiquitination of EGFR. The top panel shows ubiquitinated EGFR after EGF (10 ng/mL) or AREG (20 ng/mL) stimulation for 10 min. The middle 2 panels show western blot analysis of total and phosphorylated EGFR in whole cell lysates. (**d**) Computational model of EGFR activation. Values for dots and lines were obtained by western blot and *in silico* analyses, respectively.

We found that A-cells showed an approximately 3-fold greater increase in the EGFR protein expression compared with E-cells despite the fact that there was no significant difference in the *EGFR* mRNA expression (Fig. 5a). Further, EGFR was mainly localized in endosomes of E-cells, whereas an intense EGFR signal was detected at the plasma membrane of A-cells (Fig. 5b). Due to the different expression levels and intracellular distributions, the amount of cell surface EGFR was approximately 10-fold higher in A-cells than E-cells (Supplementary Fig. 5f,g). The different expression levels and the intracellular localization of EGFR were also observed when the doses of EGF and AREG were reduced or increased, respectively (Fig. 4b,c,f,g).

Ubiquitination plays a critical role in the endocytosis of EGFR^38^. It is known that EGF and AREG differently regulate EGFR trafficking^39^,^40^,^41^. As shown in these reports, we confirmed that AREG is much less effective than equimolar EGF at EGFR ubiquitination (Fig. 5c, lane 2 vs. lane 3 and also lane 5 vs. lane 6 in the top panel). The previous reports, however, did not address the functional significance of the accumulated EGFR on signal transduction. It should be noted that as a result of the accumulated EGFR on the cell surface, AREG-induced EGFR phosphorylation in A-cells was as efficient as EGF achieved in E-cells (Fig. 5c, lane 2 vs. lane 6 in the second panel from the top). To support this finding, we developed a computational model of EGFR phosphorylation based on the previously proposed trafficking pathway^11^. Among the parameters describing kinase signaling, the model made no distinction between EGF and AREG stimulation except for their affinities to EGFR. Our model actually showed that AREG could activate EGFR as efficiently as equimolar EGF when the amount of cell surface EGFR increased (Fig. 5d and Supplementary Fig. 6).

These results suggested that AREG-mediated EGFR accumulation on the plasma membrane compensated for the weak association between AREG and EGFR, thereby contributing to the efficient signal transduction and stimulation of cell growth (Fig. 1b).

### MEK-ERK signaling, but not TGFβ signaling, acted in the phenotypic conversion

E-cadherin plays a critical role for epithelial intercellular connections^42^. Indeed, addition of an E-cadherin neutralizing antibody disrupted cell-cell contacts and allowed A-cells to migrate as fast as EGF-cultured E-cells (Supplementary Fig. 7a,b and Supplementary Movies 2a–d), suggesting its indispensable role for the A-cell trait. We found that MEK inhibitors (U0126 and PD98059), but not phosphatidylinositol 3-kinase inhibitor (LY294002), significantly increased E-cadherin expression and promoted the formation of intercellular adhesions in EGF medium (Supplementary Fig. 7c–e). Previous studies revealed that Raf1-estrogen receptor fusion protein or Ras-V12 act on upstream of MEK and regulate EMT and cell motility^15^,^16^. As described in these reports, knockdown of either MEK1, ERK2, RSK1, RSK2 or FRA1 was confirmed to decrease the ZEB1expression and increased the E-cadherin expression (Supplementary Fig. 7f,g).

As an alternative mechanism, EGF and AREG might affect the secretion of EMT-inducing factors to different degrees and indirectly promote conversion. One possible candidate was the TGFβ that cooperates with EGF in EMT^43^. Indeed, the expression of *TGF*β*1* and β*2* mRNA was higher in E-cells than A-cells (Supplementary Table 1 and Supplementary Fig. 7h). To examine the effect of the endogenous TGFβ, E-cells were treated with SB431542. We found that the TGFβ receptor inhibition did not alter the E-cadherin expression (Supplementary Fig. 7d). Further, even in the presence of exogenous TGFβ (10 ng/mL), a 2-day culture was not sufficient to alter the E-cadherin expression or cell morphology. By contrast, after the 2-day culture with EGF, A-cells lost cell-cell contact and showed the down-regulation of E-cadherin (Supplementary Fig. 7i,j). Based on these observations and a previous report^27^, it was considered that TGFβ signaling would not play the primary role in the EGF/AREG-induced conversion. In addition, we found that the expression levels of SNAIL and SLUG in TGFβ-treated E-cells were substantially higher than those in EGF/AREG-cultured cells (Fig. 2a,b). The increased expression of SNAL and SLUG was also observed in TGFβ1-treated HMT-3522 S1 (Fig. 3d). These results raised a possibility that EGFR ligands control reversible conversion through ZEB1, whereas TGFβ may execute EMT using of a distinct set of EMT transcription factors, such as SNAIL and SLUG.

### Ligand-switching altered the integrated signal strength of EGFR-ERK pathway

**Figure 6:**
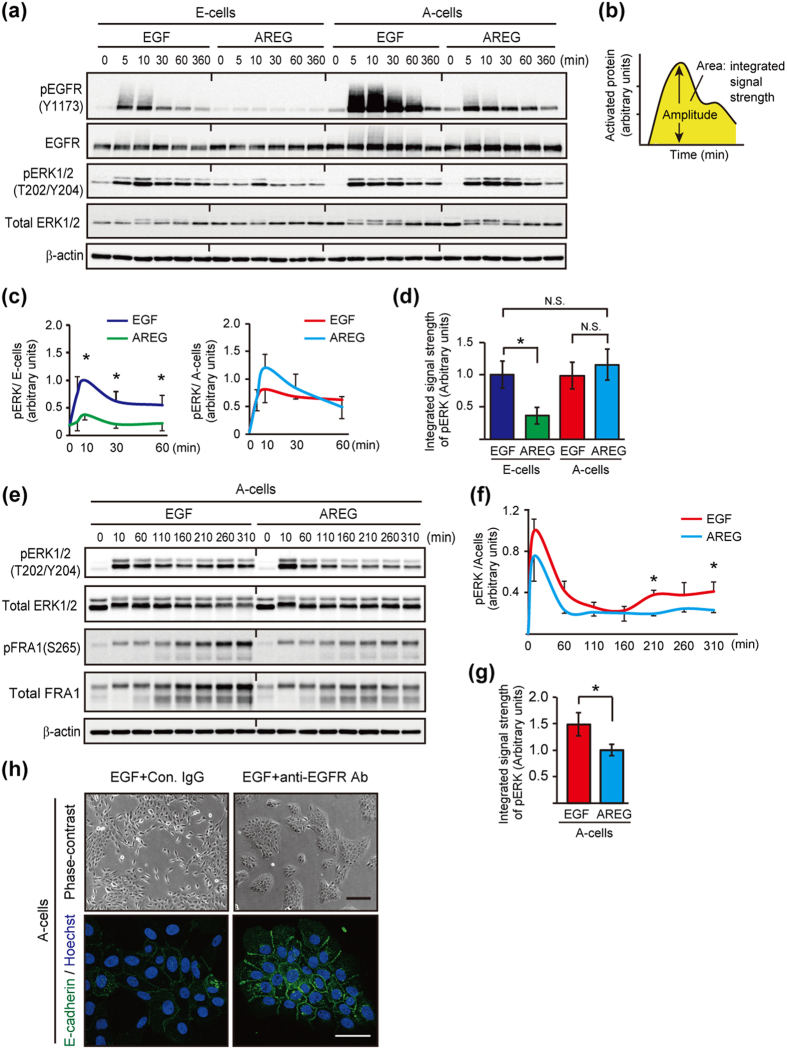
Ligand-switching between EGF and AREG altered signal strength of EGFR and ERK. (**a**) Western blot time course analysis of the phosphorylation of EGFR and ERK1/2 by 10 ng/mL EGF or 20 ng/mL AREG in E-cells and A-cells. (**b**) Schematic representation of the quantitative measurement of EGFR or ERK activation (Modified from Andreu-Perez *et al.* Fig. 1b). (**c**) Quantification of pERK phosphorylation. Data were obtained from 3 independent experiments, one of which is shown in (**a**). The band intensities were normalized to that of pERK in E-cells treated with EGF for 10 min. (**d**) Quantification of the integrated signal strength of ERK, which was calculated from the area under the curve (0 to 60 min) shown in (**c**). The values were normalized to that of pERK in E-cells treated with EGF. (**e**) Western blot analysis of the phosphorylation of ERK1/2 and FRA1 by EGF or AREG in A-cells over a time course. (**f**) Quantification of pERK phosphorylation. Data were obtained from 3 independent experiments, one of which is shown in (**e**). (**g**) Quantification of the integrated signal strength of ERK, which was calculated from the area under the curve (0 to 310 min) shown in (**f**). The values were normalized to that of pERK in A-cells treated with AREG. (**h**) Phase-contrast and immunofluorescent images of A-cells treated with EGFR inhibitors. AREG-depleted A-cells were treated with EGF or AREG, allowing the immediate activation of EGFR signaling. After the 2 h incubation, a control antibody (10 μg/mL) or a neutralizing antibody against EGFR (10 μg/mL) was administered. Cells were further cultured for 2 days and stained with an anti-E-cadherin antibody (green). Nuclei were stained with Hoechst 33342 (blue). See also Supplementary Fig. 8d. Scale bar: upper panel, 100 μm; lower panel, 50 μm.

The above results strongly suggested that EGF and AREG differently affect EGFR-ERK signaling to induce the conversion. We confirmed that both ligands could phosphorylate endogenous EGFR at 845, 1045, 1068, 1086 and 1173 (Fig. 6a and Supplementary Fig. 8a). By contrast, the amplitude of AREG-induced EGFR phosphorylation was consistently much lower than that induced by EGF. These results suggest that the conversion could be regulated by the EGFR-ERK signal strength, but not by differential phosphorylation sites in EGFR. This is consistent with our observation that the ligand-switching and the dose-changing have an equivalent effect on the phenotypic conversion (Fig. 4).

To understand the alteration of EGFR-ERK signal strength during the conversion from E-cells to A-cells, we compared the EGF-induced EGFR-ERK phosphorylation with that induced by AREG. E-cells were deprived of EGF for 8 h, and then stimulated with either EGF or AREG. Compared to the EGF control, AREG decreased the 1 h integrated signal strength of EGFR and ERK by 10- and 2.1-fold, respectively (Fig. 6a–d and Supplementary Fig. 8b,c; dark blue versus green). In a complementary experiment, A-cells were deprived of AREG for 8 h, and then stimulated with either EGF or AREG to test A-cell to E-cell conversion. Compared to the AREG control, EGF increased the 1 h integral signal strength of EGFR by 5-fold (Fig. 6a, Supplementary Fig. 8b,c; red versus light blue). In this case, however, the integrated signal strength of ERK did not show a significant difference between the EGF- and AREG-stimulated cells (Fig. 6a–d; red versus light blue). We therefore analyzed ERK phosphorylation over a 5 h period following stimulation. In addition to the immediate ERK phosphorylation, ERK re-activation began to occur after 160 min (Fig. 6e,f). The 5 h integrated signal strength of ERK in EGF-simulated cells was 1.5-fold higher than that in AREG-simulated cells (Fig. 6g). Furthermore, the amount of total and phosphorylated FRA1 protein, a transcriptional activator of the *ZEB1* gene^44^, was higher in EGF-treated cells than AREG-treated cells (Fig. 6e). The correlation between pERK and FRA1 was consistent with previous reports showing that the ERK pathway induced phosphorylation and stabilization of the FRA1 protein^45^,^46^. Taken together, these results suggested that ligand-switching induced the alteration of the integrated strength of EGFR-ERK signaling, which is associated with cell conversion.

To address whether the re-activation of the EGFR-ERK pathway was required for A-cell to E-cell conversion, A-cells were stimulated with EGF. Then, after a 2 h incubation, an EGFR neutralizing antibody or a low-dose of AG1478 (125 nM) was administrated. The partial blockages of EGFR re-activation canceled the EGF-mediated downregulation of E-cadherin and also prevented the conversion (Fig. 6h and Supplementary Fig. 8d). These data suggested that continuous signal input derived from the extracellular EGF was primarily responsible for the A-cell to E-cell conversion.

These experiments also revealed that the integrated EGFR-ERK strengths were equivalent between EGF-stimulated E-cells and AREG-stimulated A-cells (Fig. 6a–d, and Supplementary Fig. 8b,c; dark blue versus light blue), which would allow for the equivalent growth rates of E-cells and A-cells (Fig. 1b). A-cells expressed higher levels of *miR-205/200c* than E-cells, and the expression of ZEB1 protein was suppressed in A-cells (Fig. 2f–h). This mechanism was considered to be responsible for the protection of A-cells from being mesenchymal-like cells and maintenance of epithelial characteristics.

### Manipulation of the integrated signal strength of the EGFR-ERK pathway converted mammary epithelial cell characteristics

**Figure 7:**
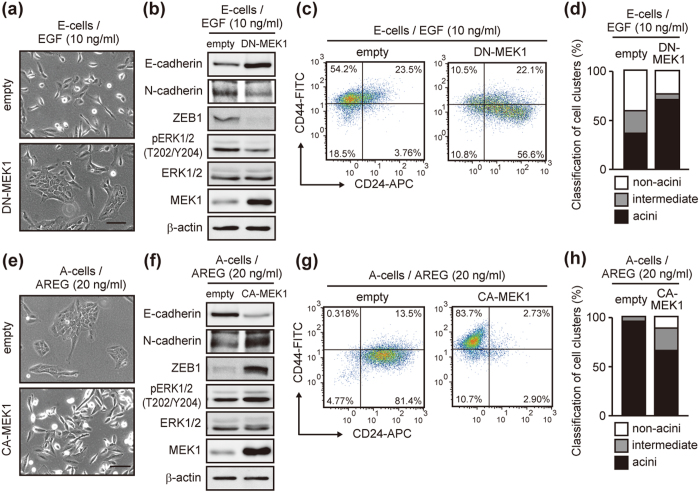
Manipulation of signal strength led to phenotypic conversion. (**a**) Phase-contrast images of E-cells infected with a lentivirus vector encoding dominant negative MEK1 (DN-MEK1). Enforced expression of DN-MEK1 increased cell-cell contact in EGF medium. Scale bar: 50 μm. (**b**) Western blot analysis of EMT-related factors, MEK1 and ERK1/2 in cells cultured as in (**a**). (**c**) Flow cytometric analysis of DN-MEK1-expressing cells cultured as in (**a**). (**d**) Quantification of acinus-formation efficiency. Cells shown in (**a**) were cultured for 2 weeks on a reconstituted basement membrane. (**e**) Phase-contrast images of A-cells infected with the lentivirus vector encoding constitutive active MEK1 (CA-MEK1). Enforced expression of CA-MEK1 decreased cell-cell contact in AREG medium. Scale bar: 50 μm. (**f**) Western blot analysis of EMT-related factors MEK1 and ERK1/2 in cells cultured as in (**e**). (**g**) Flow cytometric analysis of CA-MEK1-expressing cells cultured as in (**e**). (**h**) Quantification of acinus-formation efficiency of cells shown in (**e**). Cells were processed as in (**d**).

To directly verify the association between signal strength and phenotypic conversion, we finally modulated the signal strength by a dominant negative MEK1 (DN-MEK1; S218A/S222A) and a constitutive-active MEK1 (CA-MEK1; S218D/S222E)^47^. The DN-MEK1-overexpressing E-cells established cell-cell contact in EGF medium (Fig. 7a), which coincided with the increase of E-cadherin and the decrease of ZEB1 and N-cadherin (Fig. 7b). In contrast, CA-MEK1-overexpressing A-cells lost cell-cell contact in AREG medium (Fig. 7e), which coincided with the decrease of E-cadherin and the increase of ZEB1 and N-cadherin (Fig. 7f). Further, the main population of DN-MEK1-expressing E-cells were CD44^low^/CD24^pos^ (Fig. 7c) that formed acinus-like structures at a higher efficiency than the control cells (Fig. 7d and Supplementary Fig. 9a). In contrast, over 80% of CA-MEK1-expressing A-cells were CD44^hi^/CD24^neg^ (Fig. 7g). The acinus-formation efficiency of CA-MEK1-expressing A-cells was lower than that of the control cells (Fig. 7h and Supplementary Fig. 9a). It should be noted that, CA-MEK1 not only decreased the acinus-formation efficiency, but also generated aberrant acini with filled luminal spaces (Supplementary Fig. 9b). Our results showed that manipulation of the signal strength had a significant impact on phenotypic conversion in 2D culture and also the morphogenesis with respect to acinus-formation efficiency in 3D culture.

## Discussion

**Figure 8:**
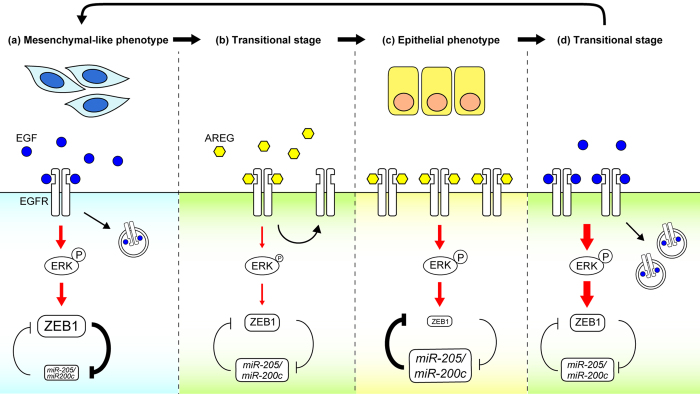
A Schematic representation of the signaling mechanism in the ligand-switching-regulated, reversible interconversion. (**a**) In E-cells, EGF-EGFR-ERK signaling activates the expression of ZEB1, which suppresses *E-cadherin* and *miR205/200c*. Under these conditions, cells exhibit mesenchymal-like characteristics. The red arrows indicate integrated signal strength. (**b**) In response to ligand-switching from EGF to AREG, the strength of EGFR-ERK signaling temporally decreases. The subsequent ZEB1 downregulation triggers the expression of *E-cadherin* and *miR205/200c*, leading to the change from mesenchymal-like to epithelial cell characteristics. Simultaneously, EGFR begins to accumulate on the plasma membrane. (**c**) AREG-mediated EGFR accumulation on the plasma membrane compensated for the weak association between AREG and EGFR, by which the EGFR-ERK strength of A-cells reaches levels almost equivalent to that of E-cells. On the other hand, A-cells expressed higher levels of *miR-205/200c* than E-cells and the expression of the ZEB1 protein is suppressed. Under these conditions, cells exhibit epithelial cell characteristics. (**d**) Ligand-switching from AREG to EGF results in an increase in integrated signal strength of the EGFR-ERK pathway. The alteration inverts the relative expression levels of ZEB1 and *miR-205/200c*, leading to a change from epithelial to mesenchymal-like cell characteristics. Simultaneously, EGFR on the plasma membrane begins to decrease. After the transitional stage (**d**), cells re-acquire mesenchymal-like cell characteristics shown in (**a**).

To date, two groups have reported that EGF and AREG induced uni-directional differentiation of human mammary epithelial cells using a 3D organoid culture of breast tissues^48^ or 2D culture of telomerase reverse transcriptase-immortalized stem/progenitor cells^49^. In both culture systems, AREG was found to support luminal cell fate, which is consistent with our current results (Fig. 3 and Supplementary Fig. 3). Although their studies implied the involvement of EGFR signal intensity in the determination of cell lineages, downstream effectors of ERK or the mechanism that regulated cell plasticity was not investigated. The present study showed that alteration of the EGFR dynamics and the EGFR-ERK strength by ligand-switching is interpreted to be the driving force that repeatedly inverts the relative expression levels of ZEB1 and *miR-205/200c*. Our findings provide a novel mechanistic link between the EGFR ligands-regulated signal strength and the ZEB/miRNAs feedback loop, enabling the reversible interconversion of distinct cell characteristics (Fig. 8).

ERK regulates cell fate decisions. In PC12 cells, transient ERK activation induced by EGF resulted in proliferation, whereas sustained activation by NGF resulted in differentiation^50^,^51^. In Swiss 3T3 fibroblasts, sustained ERK activation induced by PDGF stimulated S phase entry, whereas transient activation by EGF did not^46^. It should be noted that EGF, NGF and PDGF generated different signal strengths through their own unique receptors. Another report showed that TGFβ-treated MDCK cells could be repeatedly switched between epithelial and mesenchymal states^8^. In this case, however, different cellular states were generated by the addition and the removal of a single ligand, TGFβ1. In stark contrast, we showed that two cognate ligands generated distinctly different cell characteristics through a common signaling pathway, unambiguously demonstrating that the current model is mechanistically different from the previously reported signaling systems.

Although we have proposed a new regulatory mechanism that determines mammary epithelial cell characteristics, there remain unanswered questions about how EGFR ligands regulate cell surface EGFR, which directly affects EGFR-ERK signal strength. A previous report indicated that the EGF concentration and extent of ubiquitination can affect the internalization routes, clathrin-dependent and independent pathways^52^. It was also reported that the clathrin-dependent pathway included several redundant and interdependent mechanisms involving AP-2, Grb2 and ubiquitination^53^. These extensive studies suggest the complexity of regulation of EGFR trafficking. We believe that these multiple mechanisms collectively determine the cell surface EGFR level and the signaling strength in a cell-type- or context-dependent manner. In particular, some of these mechanisms could be involved in ERK re-activation observed in the conversion from A-cells to E-cells (Fig. 6e,f). Future studies will be needed to examine which mechanisms are dominantly used by mammary epithelial cells.

Here, we carried out *in vitro* ligand-switching as the analytical method for evaluating the action of EGF and AREG. Based on previous studies, it appears that the switching of EGFR ligands also occurs *in vivo*. In the developing mouse mammary grand, *Areg* mRNA is induced in the mature virgin and declines in late pregnancy/early lactation, followed by the induction of *Egf* mRNA^54^. Interestingly, we found that ligand-switching between EGF and AREG actively fine-tuned the amount of cell surface EGFR, a mechanism by which cells expand the dynamic range of ligand sensitivity (Fig. 6) and maintain a stable proliferation rate (Fig. 1). We speculate that the multiple ligand system could yield a greater variety of signal strength that may contribute to the generation of divergent cellular phenotypes during development. This idea might be consistent with the fact that knockout mice lacking EGFR ligands have defects in specific tissues, such as impaired ductal outgrowth of mammary glands in *Areg* KO mice^12^,^13^, wavy hair formation in *Tgf*α KO mice^55^, heart valve malformation in *Hb-egf* KO mice^56^ and dermatitis in *Ereg* KO mice^57^. Further, it was also reported that the switching of ligands, that is, down-regulation of EGF and up-regulation of AREG, occurs during the development of an early hyperplastic precursor of breast cancer^58^. Overexpression of AREG has also been described in the most common human epithelial malignancies^59^,^60^. Since EGFR ligands directly affect EGFR activity, a portion of epithelial cancers could be caused by the aberrant switching of EGFR ligands. In particular, AREG-induced EGFR accumulation on the cell surface followed by the secretion of high-affinity ligands, such as EGF or TGFα, could lead to the hyperactivation of EGFR-ERK signaling.

It has been strongly suggested that EMT is involved in metastatic events in cancer^1^,^2^. We speculate that the expression levels of EGFR ligands are relevant to EMT in some portion of cancers. MEK-targeting agents were identified as potent inhibitors of EGF-induced EMT^61^. With regard to the dosage of inhibitors, basic and clinical reports implied that near-complete (80 - 90%) inhibition of ERK signaling might be necessary to suppress tumor growth and achieve therapeutic efficacy^62^,^63^. Importantly, we showed here that EGFR-MAPK activation at a low level induced phenotypic conversion. It raised the possibility that incomplete EGFR-MAPK suppression might reverse EMT, which would increase the risk of secondary tumor induction^3^,^4^. For optimal tumor suppression, our current findings underscore the need for novel therapeutic agents against EMT as well as improved drug delivery systems that enable local administration of drugs at high-doses.

## Methods

### Cell culture

MCF10A was a generous gift of Dr. Joan Brugge. Recombinant EGF (AF-100-15) and AREG (262-AR) were purchased from PeproTech and R&D systems, respectively. According to the previous report^29^, MCF10A was cultured in growth medium (DMEM/F12 containing 5% horse serum, 0.5 μg/mL hydrocortisone, 100 ng/mL cholera toxin, 10 μg/mL insulin and EGF/AREG). In this study, EGF and AREG were used at approximately 1.8 nM (EGF: 10 ng/mL; AREG: 20 ng/mL) for routine culture. To maintain maximal growth factor activity, EGF, AREG and insulin were added just prior to use. 3D culture was performed according to the previous report^29^. Cells (5 × 10^3^ cells/well, 8well chamber) were plated on top of the solidified BD Matrigel Matrix Growth Factor Reduced (BD Biosciences) and cultured with the assay medium (DMEM/F12 containing 2% horse serum, 0.5 μg/mL hydrocortisone, 100 ng/mL cholera toxin, 10 μg/mL insulin, 5 ng/mL EGF, 2% Matrigel) for 2 weeks. HMT-3522 S1 was obtained from the Health Protection Agency and cultured according to the previous report^37^. Chemical inhibitors were purchased from the following companies: U0126 (Cell Signaling Technologies), PD98059 (Calbiochem), LY294002 (Sigma-Aldrich), SB431542 (Sigma-Aldrich). The effects of inhibitors were confirmed by Western blot analysis (Supplementary Fig. 10a,b).

### Microarray analysis

Total RNA (500 ng) was isolated from E-cells and A-cells cultured as shown Fig. 1c. GeneChip 3′ IVT expression kit (Affymetrix) was used to prepare the labeled complementary RNA. After the fragmentation, the probes were hybridized to the Human Genome U133 Plus 2.0 array (Affymetrix) according to the manufacturer’s instructions. The microarray image data were processed with the GeneChip Scanner 3000 (Affymetrix). GeneSpring GX 7.3 (Agilent Technologies) was used to analyze the gene expression profiles. DNA microarray data were shown in Supplementary Table 1.

### Western blot analysis

Cells lysates were prepared using RIPA buffer supplemented with Complete Protease Inhibitor Cocktail Tablets (Roche) and Phosphatase Inhibitor Cocktail 2 and 3 (Sigma). To observe the phosphorylation or ubiquitylation events after the growth factor stimulation (Figs 5c and 6a,e), cells were deprived of EGF/AREG for 8 h. Then, cells were stimulated by the addition of EGF (final concentration 10 ng/mL) or AREG (final concentration 20 ng/mL) for the indicated time periods and cell lysates were prepared. Protein concentration in cell lysates was measured using BCA Protein Assay kit (Thermo Scientific). Proteins were separated by SDS-PAGE and transferred to nitrocellulose membranes (Protran BA85, Whatman). After blocking with phosphoBLOCKER (Cell Biolabs) diluted in Tris-buffered saline containing 0.05% Tween20 (TBST), membranes were incubated with primary antibodies (Supplementary Table 2) diluted in TBST containing 5% BSA at 4 °C overnight. After washing with TBST, membranes were incubated with an appropriate horse radish peroxidase (HRP)-conjugated secondary antibody diluted in TBST at room temperature for 2 h. Western lighting plus-ECL (PerkinElmer) was used for the detection of HRP. Chemiluminescence signals were detected and quantified by ImageQuant LAS 4000 Scanner (GE Healthcare).

### qRT-PCR analysis

Total RNA was isolated using ISOGEN II (Nippon Gene), and reverse transcription was performed with a high-capacity RNA-to-cDNA kit (Applied Biosystems) according to the manufacture’s protocol. Real-time PCR was performed using 7500 real-time PCR system (Applied Biosystems) with FastStart Universal SYBR Green Master mixture (Roche). Based on the standard curve method, mRNA levels were measured with gene-specific primers (Supplementary Table 3) and normalized to that of GAPDH. Quantitative analysis of mature miRNAs was performed using TaqMan microRNA assays (Applied Biosystems). Total RNA was reverse transcribed with TaqMan microRNA RT kit (Assay ID: 000502, 002251, 002300, 000509). Taqman universal PCR master mix II (Applied Biosystems) was used for PCR amplification. Based on the ΔΔCT method, all miRNA data were expressed relative to that of U6 snRNA (Assay ID: 001973).

### Immunofluorescence staining and Imaging

Cells were fixed with 4% paraformaldehyde phosphate buffer solution (Nacalai Tesque) for 20 min and permeabilized with phosphate-buffered saline containing 0.1% TritonX-100 (PBST) for 5 min. After blocking using Blocking One (Nacalai Tesque), cells were incubated with primary antibodies (Supplementary Table 2) diluted in Blocking One at 4 °C overnight. After washing with PBST, cells were incubated with appropriate secondary antibodies conjugated with Alexa488 or 568. Nuclei were stained with Hoechst 33342 (Molecular Probes). Samples were mounted with ProLong Gold antifade reagent (Invitrogen). Immunostaining for 3D culture was performed according to previous report^29^. Images were acquired using the A1R laser confocal microscope (Nikon). Data analyses were performed using NIS-Elements program (Nikon). In the live imaging experiments, cells were plated on μ-Dish^35mm, high^ (ibidi). Live cell images were captured by the Biostation IM (Nikon) and analyzed using the Volocity software (PerkinElmer). Conventional phase-contrast images were captured by the IX70 inverted microscope (Olympus).

### Transfection of siRNAs and microRNA inhibitors

Cells were reverse transfected with siRNA (20 nM) or microRNA inhibitors (20 nM) using Lipofectamine RNAiMAX (Invitrogen) according to manufacturer’s protocol, and seeded at 5 × 10^5^ cells per 60 mm dishes. Total RNA or whole cell lysates were prepared after the 2-day culture. Information about siRNA and synthetic microRNA inhibitors was shown in Supplementary Tables 4 and 5, respectively. Knockdown of target proteins was confirmed by Western blot analysis (Supplementary Fig. 10d).

### Lentiviral expression

MEK1 mutants were expressed by the lentiviral system^64^. The human *MEK1* coding region (wild-type) was amplified using cDNA prepared from MCF10A. Then, amino acid substitution was introduced by PCR mutagenesis. The cDNA fragments was subcloned into the lentiviral expression vector, CSII-EF-MCS-IRES2-Bsd, and then transfected into 293T cells together with two packaging plasmids, pCAG-HIVgp and pCMV-VSV-G-RSV-Rev. MCF10A cells were infected with the MEK1 mutant-expressing lentiviruses and the infected cells were maintained in the presence of blasticidin at 5 μg/mL.

### Flow cytometry

Cells were detached from culture dishes by trypsin and re-suspended in ice-cold PBS containing 2% fetal bovine serum. Single cell suspensions were stained with antibodies (Supplementary Table 2) for 20 min on ice. After washing, cells were analyzed by Gallios Flow Cytometer (Beckman Coulter) and FlowJo software (FlowJo). For sorting experiments, subpopulations were purified using FACS Aria instrument (BD Bioscience).

### Quantification of cell clustering

To measure the degree of cell clustering, we used “nucleus-nucleus distance index”, which was computed by dividing the number of cell clusters by the total number of nuclei (Supplementary Fig. 1e, see equation 1). The index is close to 1.0 when cells are sparsely distributed while it is close to 0 as cells become clustered. For this measurement, we first randomly selected images with Hoechst-labeled nuclei and they were binarized using intensity-based automatic image threshold by Otsu algorithm followed by morphological fill-hole and open operations. We counted nucleus number (N0) using the resulted image. We then artificially expanded areas of nucleus using dilate-operation by five pixels. We chose the five-pixels dilation because this distance corresponded approximately to a half the size of cells. If cells were in close proximity to their neighbors and clustered, their nuclei became single object by this dilation, while nuclei of cells remote from others remained as a single object. We counted the number of objects after dilation operation as the cluster number (N5),


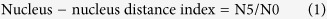


Image analysis was automatically performed by a custom Jython script using ImageJ^65^. This script can be freely downloaded (http://dx.doi.org/10.5281/zenodo.18246).

### Simulation of EGFR phosphorylation and Parameter Estimation

We developed the EGFR activation model based on the law of mass action, and performed simulation and parameter estimations using Matlab (version R2013b, Math Works). The parameters of the model were estimated using experimental data (Fig. 6a) according to two methods, a meta-evolutionary programming method and the Nelder-Mead method^66^. With these methods, the parameters were estimated to minimize the objective function value, which is defined as the sum of the square residuals between our measurements and the model trajectories. After 200 independent estimations for the model, we selected the model that had the minimum objective function value.

### Statistics

All data are presented as mean ± S.D. When two groups were compared, the Student’s t test was used. Values of p < 0.05 were considered statistically significant.

## Additional Information

**How to cite this article**: Fukuda, S. *et al.* Reversible interconversion and maintenance of mammary epithelial cell characteristics by the ligand-regulated EGFR system. *Sci. Rep.* **6**, 20209; doi: 10.1038/srep20209 (2016).

## Supplementary Material

Supplementary Information

Supplementary Movie 1

Supplementary Movie 2

Supplementary Movie 3

Supplementary Movie 4

Supplementary Movie 5

Supplementary Movie 6

## References

[b1] Thiery J. P., Acloque H., Huang R. Y. & Nieto M. A. Epithelial-mesenchymal transitions in development and disease. Cell 139, 871–890 (2009). 19945376 10.1016/j.cell.2009.11.007

[b2] Nieto M. A. Epithelial plasticity: a common theme in embryonic and cancer cells. Science 342, 1234850 (2013). 24202173 10.1126/science.1234850

[b3] Tsai J. H., Donaher J. L., Murphy D. A., Chau S. & Yang J. Spatiotemporal regulation of epithelial-mesenchymal transition is essential for squamous cell carcinoma metastasis. Cancer Cell 22, 725–736 (2012). 23201165 10.1016/j.ccr.2012.09.022 PMC3522773

[b4] Ocana O. H. *et al.* Metastatic colonization requires the repression of the epithelial-mesenchymal transition inducer Prrx1. Cancer Cell 22, 709–724 (2012). 23201163 10.1016/j.ccr.2012.10.012

[b5] Heldin C. H., Vanlandewijck M. & Moustakas A. Regulation of EMT by TGFbeta in cancer. FEBS Lett 586, 1959–1970 (2012). 22710176 10.1016/j.febslet.2012.02.037

[b6] Burk U. *et al.* A reciprocal repression between ZEB1 and members of the miR-200 family promotes EMT and invasion in cancer cells. EMBO Rep 9, 582–589 (2008). 18483486 10.1038/embor.2008.74 PMC2396950

[b7] Gregory P. A. *et al.* The miR-200 family and miR-205 regulate epithelial to mesenchymal transition by targeting ZEB1 and SIP1. Nat Cell Biol 10, 593–601 (2008). 18376396 10.1038/ncb1722

[b8] Gregory P. A. *et al.* An autocrine TGF-beta/ZEB/miR-200 signaling network regulates establishment and maintenance of epithelial-mesenchymal transition. Mol Biol Cell 22, 1686–1698 (2011). 21411626 10.1091/mbc.E11-02-0103 PMC3093321

[b9] Macara I. G., Guyer R., Richardson G., Huo Y. & Ahmed S. M. Epithelial homeostasis. Curr Biol 24, R815–825 (2014). 25202877 10.1016/j.cub.2014.06.068 PMC4196707

[b10] Higashiyama S. *et al.* Membrane-anchored growth factors, the epidermal growth factor family: beyond receptor ligands. Cancer Sci 99, 214–220 (2008). 18271917 10.1111/j.1349-7006.2007.00676.x PMC11158050

[b11] Citri A. & Yarden Y. EGF-ERBB signalling: towards the systems level. Nat Rev Mol Cell Biol 7, 505–516 (2006). 16829981 10.1038/nrm1962

[b12] Ciarloni L., Mallepell S. & Brisken C. Amphiregulin is an essential mediator of estrogen receptor alpha function in mammary gland development. Proc Natl Acad Sci U S A 104, 5455–5460 (2007). 17369357 10.1073/pnas.0611647104 PMC1838509

[b13] Luetteke N. C. *et al.* Targeted inactivation of the EGF and amphiregulin genes reveals distinct roles for EGF receptor ligands in mouse mammary gland development. Development 126, 2739–2750 (1999). 10331984 10.1242/dev.126.12.2739

[b14] Shoyab M., Plowman G. D., McDonald V. L., Bradley J. G. & Todaro G. J. Structure and function of human amphiregulin: a member of the epidermal growth factor family. Science 243, 1074–1076 (1989). 2466334 10.1126/science.2466334

[b15] Shin S., Dimitri C. A., Yoon S. O., Dowdle W. & Blenis J. ERK2 but not ERK1 induces epithelial-to-mesenchymal transformation via DEF motif-dependent signaling events. Mol Cell 38, 114–127 (2010). 20385094 10.1016/j.molcel.2010.02.020 PMC2854677

[b16] Doehn U. *et al.* RSK is a principal effector of the RAS-ERK pathway for eliciting a coordinate promotile/invasive gene program and phenotype in epithelial cells. Mol Cell 35, 511–522 (2009). 19716794 10.1016/j.molcel.2009.08.002 PMC3784321

[b17] Isokane M. *et al.* Plasma-membrane-anchored growth factor pro-amphiregulin binds A-type lamin and regulates global transcription. J Cell Sci 121, 3608–3618 (2008). 18946024 10.1242/jcs.031443

[b18] Nakayama H. *et al.* Cell surface annexins regulate ADAM-mediated ectodomain shedding of proamphiregulin. Mol Biol Cell 23, 1964–1975 (2012). 22438584 10.1091/mbc.E11-08-0683 PMC3350559

[b19] Fukuda S., Nishida-Fukuda H., Nakayama H., Inoue H. & Higashiyama S. Monoubiquitination of pro-amphiregulin regulates its endocytosis and ectodomain shedding. Biochem Biophys Res Commun 420, 315–320 (2012). 22425981 10.1016/j.bbrc.2012.02.156

[b20] Nakayama H. *et al.* Human antigen R-mediated mRNA stabilization is required for ultraviolet B-induced autoinduction of amphiregulin in keratinocytes. J Biol Chem 288, 10338–10348 (2013). 23430747 10.1074/jbc.M112.417527 PMC3624417

[b21] Hieda M. *et al.* Membrane-anchored growth factor, HB-EGF, on the cell surface targeted to the inner nuclear membrane. J Cell Biol 180, 763–769 (2008). 18299347 10.1083/jcb.200710022 PMC2373455

[b22] Nanba D., Mammoto A., Hashimoto K. & Higashiyama S. Proteolytic release of the carboxy-terminal fragment of proHB-EGF causes nuclear export of PLZF. J Cell Biol 163, 489–502 (2003). 14597771 10.1083/jcb.200303017 PMC2173632

[b23] Soule H. D. *et al.* Isolation and characterization of a spontaneously immortalized human breast epithelial cell line, MCF-10. Cancer Res 50, 6075–6086 (1990). 1975513

[b24] Tait L., Soule H. D. & Russo J. Ultrastructural and immunocytochemical characterization of an immortalized human breast epithelial cell line, MCF-10. Cancer Res 50, 6087–6094 (1990). 1697506

[b25] Sarrio D. *et al.* Epithelial-mesenchymal transition in breast cancer relates to the basal-like phenotype. Cancer Res 68, 989–997 (2008). 18281472 10.1158/0008-5472.CAN-07-2017

[b26] Maeda M., Johnson K. R. & Wheelock M. J. Cadherin switching: essential for behavioral but not morphological changes during an epithelium-to-mesenchyme transition. J Cell Sci 118, 873–887 (2005). 15713751 10.1242/jcs.01634

[b27] Brown K. A. *et al.* Induction by transforming growth factor-beta1 of epithelial to mesenchymal transition is a rare event *in vitro*. Breast Cancer Res 6, R215–231 (2004). 15084245 10.1186/bcr778 PMC400675

[b28] Hosono S. *et al.* E-cadherin is a WT1 target gene. J Biol Chem 275, 10943–10953 (2000). 10753894 10.1074/jbc.275.15.10943

[b29] Debnath J., Muthuswamy S. K. & Brugge J. S. Morphogenesis and oncogenesis of MCF-10A mammary epithelial acini grown in three-dimensional basement membrane cultures. Methods 30, 256–268 (2003). 12798140 10.1016/s1046-2023(03)00032-x

[b30] Shackleton M. *et al.* Generation of a functional mammary gland from a single stem cell. Nature 439, 84–88 (2006). 16397499 10.1038/nature04372

[b31] Stingl J. *et al.* Purification and unique properties of mammary epithelial stem cells. Nature 439, 993–997 (2006). 16395311 10.1038/nature04496

[b32] Lim E. *et al.* Aberrant luminal progenitors as the candidate target population for basal tumor development in BRCA1 mutation carriers. Nat Med 15, 907–913 (2009). 19648928 10.1038/nm.2000

[b33] Al-Hajj M., Wicha M. S., Benito-Hernandez A., Morrison S. J. & Clarke M. F. Prospective identification of tumorigenic breast cancer cells. Proc Natl Acad Sci USA 100, 3983–3988 (2003). 12629218 10.1073/pnas.0530291100 PMC153034

[b34] Mani S. A. *et al.* The epithelial-mesenchymal transition generates cells with properties of stem cells. Cell 133, 704–715 (2008). 18485877 10.1016/j.cell.2008.03.027 PMC2728032

[b35] Chaffer C. L. *et al.* Poised chromatin at the ZEB1 promoter enables breast cancer cell plasticity and enhances tumorigenicity. Cell 154, 61–74 (2013). 23827675 10.1016/j.cell.2013.06.005 PMC4015106

[b36] Liu S. *et al.* Breast cancer stem cells transition between epithelial and mesenchymal states reflective of their normal counterparts. Stem Cell Reports 2, 78–91 (2014). 24511467 10.1016/j.stemcr.2013.11.009 PMC3916760

[b37] Vidi P. A., Bissell M. J. & Lelievre S. A. Three-dimensional culture of human breast epithelial cells: the how and the why. Methods Mol Biol 945, 193–219 (2013). 23097109 10.1007/978-1-62703-125-7_13 PMC3666567

[b38] Haglund K. & Dikic I. The role of ubiquitylation in receptor endocytosis and endosomal sorting. J Cell Sci 125, 265–275 (2012). 22357968 10.1242/jcs.091280

[b39] Roepstorff K. *et al.* Differential effects of EGFR ligands on endocytic sorting of the receptor. Traffic 10, 1115–1127 (2009). 19531065 10.1111/j.1600-0854.2009.00943.x PMC2723868

[b40] Stern K. A., Place T. L. & Lill N. L. EGF and amphiregulin differentially regulate Cbl recruitment to endosomes and EGF receptor fate. Biochem J 410, 585–594 (2008). 18045238 10.1042/BJ20071505 PMC3507514

[b41] Willmarth N. E. *et al.* Altered EGFR localization and degradation in human breast cancer cells with an amphiregulin/EGFR autocrine loop. Cell Signal 21, 212–219 (2009). 18951974 10.1016/j.cellsig.2008.10.003 PMC2632975

[b42] Takeichi M. Dynamic contacts: rearranging adherens junctions to drive epithelial remodelling. Nat Rev Mol Cell Biol 15, 397–410 (2014). 24824068 10.1038/nrm3802

[b43] Zavadil J. & Bottinger E. P. TGF-beta and epithelial-to-mesenchymal transitions. Oncogene 24, 5764–5774 (2005). 16123809 10.1038/sj.onc.1208927

[b44] Caramel J. *et al.* A switch in the expression of embryonic EMT-inducers drives the development of malignant melanoma. Cancer Cell 24, 466–480 (2013). 24075834 10.1016/j.ccr.2013.08.018

[b45] Basbous J., Chalbos D., Hipskind R., Jariel-Encontre I. & Piechaczyk M. Ubiquitin-independent proteasomal degradation of Fra-1 is antagonized by Erk1/2 pathway-mediated phosphorylation of a unique C-terminal destabilizer. Mol Cell Biol 27, 3936–3950 (2007). 17371847 10.1128/MCB.01776-06 PMC1900028

[b46] Murphy L. O., Smith S., Chen R. H., Fingar D. C. & Blenis J. Molecular interpretation of ERK signal duration by immediate early gene products. Nat Cell Biol 4, 556–564 (2002). 12134156 10.1038/ncb822

[b47] Torii S., Kusakabe M., Yamamoto T., Maekawa M. & Nishida E. Sef is a spatial regulator for Ras/MAP kinase signaling. Dev Cell 7, 33–44 (2004). 15239952 10.1016/j.devcel.2004.05.019

[b48] Pasic L. *et al.* Sustained activation of the HER1-ERK1/2-RSK signaling pathway controls myoepithelial cell fate in human mammary tissue. Genes Dev 25, 1641–1653 (2011). 21828273 10.1101/gad.2025611 PMC3182019

[b49] Mukhopadhyay C., Zhao X., Maroni D., Band V. & Naramura M. Distinct effects of EGFR ligands on human mammary epithelial cell differentiation. PLoS One 8, e75907 (2013). 24124521 10.1371/journal.pone.0075907 PMC3790811

[b50] Vaudry D., Stork P. J., Lazarovici P. & Eiden L. E. Signaling pathways for PC12 cell differentiation: making the right connections. Science 296, 1648–1649 (2002). 12040181 10.1126/science.1071552

[b51] Andreu-Perez P. *et al.* Protein arginine methyltransferase 5 regulates ERK1/2 signal transduction amplitude and cell fate through CRAF. Sci Signal 4, ra58 (2011). 21917714 10.1126/scisignal.2001936 PMC6312726

[b52] Sigismund S. *et al.* Clathrin-mediated internalization is essential for sustained EGFR signaling but dispensable for degradation. Dev Cell 15, 209–219 (2008). 18694561 10.1016/j.devcel.2008.06.012

[b53] Goh L. K., Huang F., Kim W., Gygi S. & Sorkin A. Multiple mechanisms collectively regulate clathrin-mediated endocytosis of the epidermal growth factor receptor. J Cell Biol 189, 871–883 (2010). 20513767 10.1083/jcb.201001008 PMC2878939

[b54] Schroeder J. A. & Lee D. C. Dynamic expression and activation of ERBB receptors in the developing mouse mammary gland. Cell Growth Differ 9, 451–464 (1998). 9663464

[b55] Mann G. B. *et al.* Mice with a null mutation of the TGF alpha gene have abnormal skin architecture, wavy hair, and curly whiskers and often develop corneal inflammation. Cell 73, 249–261 (1993). 8477444 10.1016/0092-8674(93)90227-h

[b56] Iwamoto R. *et al.* Heparin-binding EGF-like growth factor and ErbB signaling is essential for heart function. Proc Natl Acad Sci USA 100, 3221–3226 (2003). 12621152 10.1073/pnas.0537588100 PMC152273

[b57] Shirasawa S. *et al.* Dermatitis due to epiregulin deficiency and a critical role of epiregulin in immune-related responses of keratinocyte and macrophage. Proc Natl Acad Sci USA 101, 13921–13926 (2004). 15365177 10.1073/pnas.0404217101 PMC518854

[b58] Lee S. *et al.* Alterations of gene expression in the development of early hyperplastic precursors of breast cancer. Am J Pathol 171, 252–262 (2007). 17591970 10.2353/ajpath.2007.061010 PMC1941596

[b59] Berasain C. & Avila M. A. & Amphiregulin. Semin Cell Dev Biol 28, 31–41 (2014). 24463227 10.1016/j.semcdb.2014.01.005

[b60] Busser B., Sancey L., Brambilla E., Coll J. L. & Hurbin A. The multiple roles of amphiregulin in human cancer. Biochim Biophys Acta 1816, 119–131 (2011). 21658434 10.1016/j.bbcan.2011.05.003

[b61] Chua K. N. *et al.* A cell-based small molecule screening method for identifying inhibitors of epithelial-mesenchymal transition in carcinoma. PLoS One 7, e33183 (2012). 22432005 10.1371/journal.pone.0033183 PMC3303807

[b62] Albeck J. G., Mills G. B. & Brugge J. S. Frequency-modulated pulses of ERK activity transmit quantitative proliferation signals. Mol Cell 49, 249–261 (2013). 23219535 10.1016/j.molcel.2012.11.002 PMC4151532

[b63] Bollag G. *et al.* Clinical efficacy of a RAF inhibitor needs broad target blockade in BRAF-mutant melanoma. Nature 467, 596–599 (2010). 20823850 10.1038/nature09454 PMC2948082

[b64] Miyoshi H., Blomer U., Takahashi M., Gage F. H. & Verma I. M. Development of a self-inactivating lentivirus vector. J Virol 72, 8150–8157 (1998). 9733856 10.1128/jvi.72.10.8150-8157.1998 PMC110156

[b65] Schindelin J. *et al.* Fiji: an open-source platform for biological-image analysis. Nat Methods 9, 676–682 (2012). 22743772 10.1038/nmeth.2019 PMC3855844

[b66] Kubota H. *et al.* Temporal coding of insulin action through multiplexing of the AKT pathway. Mol Cell 46, 820–832 (2012). 22633957 10.1016/j.molcel.2012.04.018

