# Supplementary material for: Reversible interconversion and maintenance of mammary epithelial cell characteristics by the ligand-regulated EGFR system
Source: Sci Rep. 2016 Feb 2;6:20209. doi: 10.1038/srep20209 (PMC4735799; doi:10.1038/srep20209)
Supplement: Supplementary Information [file srep20209-s1.pdf]

Supplementary Materials

Reversible interconversion and maintenance of mammary epithelial cell characteristics by the ligand-regulated EGFR system

Shinji Fukuda, Hisayo Nishida-Fukuda, Daisuke Nanba, Koh-ichi Nakashiro, Hironao Nakayama, Hiroyuki Kubota, Shigeki Higashiyama

Contents

Supplementary Figure legends..................page 1-6

Supplementary Figures 1 to 10..................page 7-16

Supplementary Table 1..............................page 17-66

Supplementary Table 2 to 5................ ......page 67-70

Supplementary Figure 1. Growth properties, migration speed and cluster formation of E-cells and A-cells

(a) Representative time-lapse images of MCF10A cultured in the presence (upper panels) or absence (lower panels) of recombinant EGF. MCF10A cells require recombinant EGF, and insulin and horse serum are not sufficient for their proliferation. Scale bar: 50 μm.

(b) Illustrations of the detailed process of cell culture. Parental MCF10A cells were cultured in parallel in the presence of EGF (10 ng/mL [1.8 nM]) or AREG (20 ng/mL [1.8 nM]). "Subculture" denotes the trypsinization and replating of the cells in new culture dishes every 3 days. After ligand-switching (subculture 1), we further repeated the subculture at least 3 times, that is, cells were kept for at least 12 days under the individual conditions. Then, the next round of ligand-switching was performed.

(c) Cell number of parental MCF10A cultured in the presence of EGF (10 ng/mL) or AREG (20 ng/mL). The values in this graph were used to calculate the relative growth rate of MCF10A cultured with EGF or AREG (see Fig. 1b).

(d) The migration speed of E-cells and A-cells calculated by the live imaging. *: $p<0.05$. See Supplementary Movies 1a and 1b.

(e) An example of the quantification of cell clustering. Randomly selected Hoechst staining images (left panels) were binarized (middle panels), and the numbers of nuclei were counted (N0). In

this figure, the values of N0 in E-cells (upper middle panel) and A-cells (lower middle pane) are 241 and 216, respectively. Next, the areas of nucleus were artificially expanded by five pixels, and the numbers of clusters were counted (N5). In this figure, the values of N5 in E-cells (upper middle panel) and A-cells (lower middle pane) are 205 and 87, respectively. The nucleus-nucleus distance index was defined as the division of N5 by N0 (see Methods for further detail).

Supplementary Figure 2. Expression of EMT related molecules in E-cells and A-cells

(a) Left: RT-qPCR analysis of the *Twist1* and *ZEB2* expression. *: $p < 0.05$. Right: Western blot analysis of the Twist1 and ZEB2 expression. NIH3T3 and human fibroblast cell lysates were used as positive controls.

(b) The E-cadherin promoter assay. Cells were transfected with human E-cadherin promoter-firefly luciferase reporter plasmid and an internal Renilla luciferase plasmid, together with or without the ZEB1 expression plasmid. After 24 h, luciferase activity was measured. Left: The E-cadherin promoter activity of A-cells was higher than that of E-cells. Right: The E-cadherin promoter activity was suppressed by the ZEB1 overexpression in A-cells.

(c) Left: RT-qPCR analysis of the mature *miR-200* family and *miR-205* expression. Right: The average Ct values of *miR-200* family and *miR-205* in E-cells and A-cells.

(d) Western blot analysis of the change of the expression of EMT markers and EGFR after the ligand-switching from EGF to AREG. The left lane shows the expression of these proteins in E-cells (before the ligand-switching).

(e) RT-qPCR analysis of the change of the mature *miR-200* family and *miR-205* expression after the ligand-switching from EGF to AREG. The left back bar shows the expression of these miRNAs in E-cells (before the ligand-switching).

(f) Phase-contrast images of 4 independent clones established by limiting dilution (left panels). Then, the E-cells were converted into A-cells (middle panels), and further into E-cells (2nd) (right panels) by the ligand-switching. Scale bar: 100 μm.

(g) Western blot analysis of EMT related factors and EGFR in the 4 clones shown in (f).

(h) Western blot analysis of E-cadherin and ZEB1 in E-cells (2nd) and E-cells (3rd) before and after the long subculture. E-cells (2nd) and E-cells (3rd) at subculture 6 were maintained for the additional 6 passages. Then, the expression levels of EMT markers were compared between the early- (subculture 6) and the late- (subculture 12) passage populations.

Supplementary Figure 3. Characterization of E-cells and A-cells by 3D assay and FACS analysis

(a) Left: The size distribution of cell clusters after 2-week culture on the reconstituted basement membrane. Right: The projected Z-stack images of cell clusters stained with fluorescent phalloidin (green) and Hoechst 33342 (blue). Maximum cross-section area was calculated by NIS-Elements (Nikon). Non-acini cell cluster (<500 μm^2) composed of two to three cells, whereas acini-like cell cluster (2000-3000 μm^2) showed the well-organized structure.

(b) Left: $CD44^{hi}/CD24^{neg}$ cells and $CD44^{lo}/CD24^{pos}$ cells in E-cells were separated by FACS. The sorted cells were immediately lysed and the expression of EMT related factors and EGFR were analyzed by western blotting. Right: Quantification of the acini formation efficiency of the sorted cells.

(c) FACS analysis of the expression of EpCAM and CD49f in sequentially generated E-cells and A-cells.

Supplementary Figure 4. Effects of the different concentrations of EGF and AREG on the acinus-formation

(a) The projected Z-stack images of acini formation assay using MCF10A cultured in the presence of various concentration of EGF and AREG. Cell clusters were stained with fluorescent phalloidin. Yellow arrow heads indicate cell clusters judged as non-acini. Scale bar: 100 μm.

(b) Expression of ZEB1 induced by EGF or AREG. Cells were deprived for EGF for 24 h. Then, cells were treated with EGF (10 ng/mL) or AREG (20 ng/mL). After the 24 h or 48 h incubation, cell lysates were prepared and subjected to the western blot analysis with anti-ZEB1 antibodies.

Supplementary Figure 5. Roles of the ErbB family members in the phenotypic conversion

(a) Phase-contrast images of E-cells treated with AG1478 (1 μM) or DMSO after 48 h. Scale bar: 50 μm.

(b) RT-qPCR analyses of the knockdown of *ErbB2* (upper graph) and *ErbB3* (lower graph) expression in E-cells.

(c) Western blot analyses of the effect of ErbB2 and ErbB3 knockdown on the expression of E-cadherin in E-cells. The knockdown of ErbB2 at the protein level was also shown. The expression of ErbB3 protein was not detected.

(d) Phase-contrast images of E-cells transfected with control, *ErbB2* and *ErbB3* siRNA after 48 h. Scale bar: 50 μm.

(e) RT-qPCR analysis of *ErbB2, ErbB3* and *ErbB4* mRNA level in E-cells and A-cells. The expression of *ErbB4* mRNA was not detected. *: $p < 0.05$.

(f) Flow cytometric analysis of cell surface EGFR expression in E-cells and A-cells. The gray histogram signifies staining with isotype control.

(g) Biotinylation assay for the detection of the cell surface EGFR in E-cells and A-cells. Under the steady state growth condition, cell surface proteins of E-cells and A-cells were labeled with 0.01% sulfo-NHS-biotin (Pierce) in 0.1 M HEPES and 0.15 M NaCl (pH 8.0) for 15 min at 4°C. Excess reagent was quenched and removed with ice-cold DMEM/F12 containing 20% horse serum. Cells were lysed in RIPA buffer and the lysates were subjected to immunoprecipitation using an antibody against EGFR. After the SDS-PAGE and western blotting, cell surface EGFR was detected by HRP-conjugated streptavidin (VECTOR LABORATORIES). Aliquots of cell lysates were analyzed by the standard western blotting using an anti-EGFR antibody.

Supplementary Figure 6. Computational model of the EGFR phosphorylation

(a) Schematic overview of the EGFR model. A boxed arrow and a lined circle represent a directional biochemical reaction and a pool of substrate, respectively. “Re#” indicates the reaction number.

(b) Reactions and rate constants in the EGFR model. “Re” indicates the reaction number.

(c) Initial amounts of molecules in the EGFR model.

(d) Scale factor of pEGFR in the EGFR model.

(e) Ordinary differential equations in the EGFR model. “Re” indicates the reaction number.

Supplementary Figure 7. Roles of EGFR-ERK and other signaling pathways in the regulation of E-cadherin expression

(a) Representative images of E-cells and A-cells cultured for 48 h in the presence of control IgG (4 μg/mL) or an E-cadherin neutralizing antibody (4 μg/mL). Cells were plated in the growth medium and cultured for 6 h. Then, the control or neutralizing antibodies were administrated. Image acquisition was started just after the addition of antibodies. See Supplementary Movies 2a to 2d. Scale bar: 50 μm.

(b) The migration speed of E-cells and A-cells in the presence of control IgG or an E-cadherin neutralizing antibody calculated by the live imaging. *: $p<0.05$.

(c) RT-qPCR analysis of *E-cadherin* expression. E-cells were treated with 5 μM U0126 (MEK1/2 inhibitor), 50 μM PD98059 (MEK1/2 inhibitor), 50 μM LY294002 (Akt inhibitor) or 10 μM

SB431542 (TGFβR inhibitor). Total RNA was isolated after the 2-day culture. The *E-cadherin* mRNA levels of inhibitor-treated cells were expressed relative to that of DMSO-treated cells.

(d) Western blot analysis of E-cadherin in E-cells treated with inhibitors as in (c).

(e) Phase-contrast images of E-cells treated with DMSO or PD98059. After the 2-day culture, cell-cell contacts were established in the presence of PD98059 in EGF medium. Scale bar: 50 μm.

(f) Western blot analysis of E-cadherin and ZEB1 in E-cells transfected with siRNAs against MEK-ERK signaling molecules after the 2-day culture.

(g) Immunofluorescent images of E-cells transfected with siRNAs against MEK-ERK signaling molecules. After the 2-day culture, cells were stained with an anti-E-cadherin antibody (green). Nuclei were stained with Hoechst 33342 (blue). Scale bar: 20 μm.

(h) RT-qPCR analysis of *TGF-β1*, *β2* and *β3* mRNA levels in E-cells and A-cells. *: $p < 0.05$.

(i) Western blot analysis of E-cadherin in recombinant TGFβ-treated A-cells. Ligand-switching from AREG to EGF significantly decreased the expression of E-cadherin, whereas no additional reduction was observed in the presence of recombinant TGFβ1 (10 ng/mL) together with EGF in the 2-day culture.

(j) Phase-contrast images of A-cells treated with recombinant TGFβ1 (10 ng/mL) together with EGF or AREG. The 2-day TGFβ1-treatment had no significant effect on the cell-cell adhesion. Scale bar: 100 μm.

Supplementary Figure 8. Signal strength of EGFR activation

(a) Western blot analysis of the phosphorylation of EGFR (pY845, pY1045, pY1068, pY1086) by EGF (10 ng/mL) or AREG (20 ng/mL) in E-cells and A-cells. See Fig. 6a.

(b) Quantification of the EGFR phosphorylation (pY1173). Data were obtained from 3 independent experiments, one of which was shown in Fig. 6a. The band intensities were normalized to that of pEGFR in E-cells treated with EGF for 10 min.

(c) Quantification of the integrated signal strength of EGFR (pY1173) calculated from data shown in (b). The values were normalized to that of pEGFR in E-cells treated with EGF.

(d) Phase-contrast and immunofluorescent images of A-cells treated with an EGFR inhibitor, AG1478. AREG-deprived A-cells were treated with EGF or AREG, allowing the immediate activation of EGFR signaling. After the 2 h incubation, AG1478 (125 nM) were administered. Cells were further cultured for 2 days and stained with E-cadherin antibodies (green). Nuclei were stained with Hoechst 33342 (blue). Scale bar: upper panel, 100 μm; Lower panel, 50 μm.

Supplementary Figure 9. Effects of the expression of MEK1 mutants on acini formation

(a) The projected Z-stack images of acini formation assay using DN-MEK1 or CA-MEK1-expressing cells. Cell clusters were stained with fluorescent phalloidin. Yellow arrowheads indicate cell clusters judged as non-acini. Scale bar: 100 μm.

(b) The confocal images of cell clusters stained with fluorescentphalloidin (green) and Hoechst 33342 (blue). Note that acini of empty and DN-MEK1-expressing cells were well organized, whereas that of CA-MEK1-expressing cells showed luminal-filling phenotype. Scale bar: 50 μm.

Supplementary Figure 10. Validation of chemical inhibitors and siRNAs

(a) E-cells were treated with chemical inhibitors for 30 min at the following concentrations: U0126, 5 μM; PD98059, 50 μM; LY294002 50 μM; SB431542, 10 μM. Cells were further treated with EGF (10 ng/mL) for 20 min, and then cell lysates were prepared for the western blot analysis. The effect of SB431542 was also shown in (b).

(b) E-cells were treated with SB431542 (10 μM) for 30 min. Cells were further treated with recombinant TGFβ (10 ng/mL) for 20 min, and then cell lysates were prepared for the western blot analysis.

(c) E-cells were reverse transfected with siRNA (20nM). After the 2-day culture, whole cell lysates were prepared for the western blot analysis. The expression levels of ZEB1 and β-actin in the same lysates were shown in Supplementary Fig. 7f.

Supplementary Figure 1

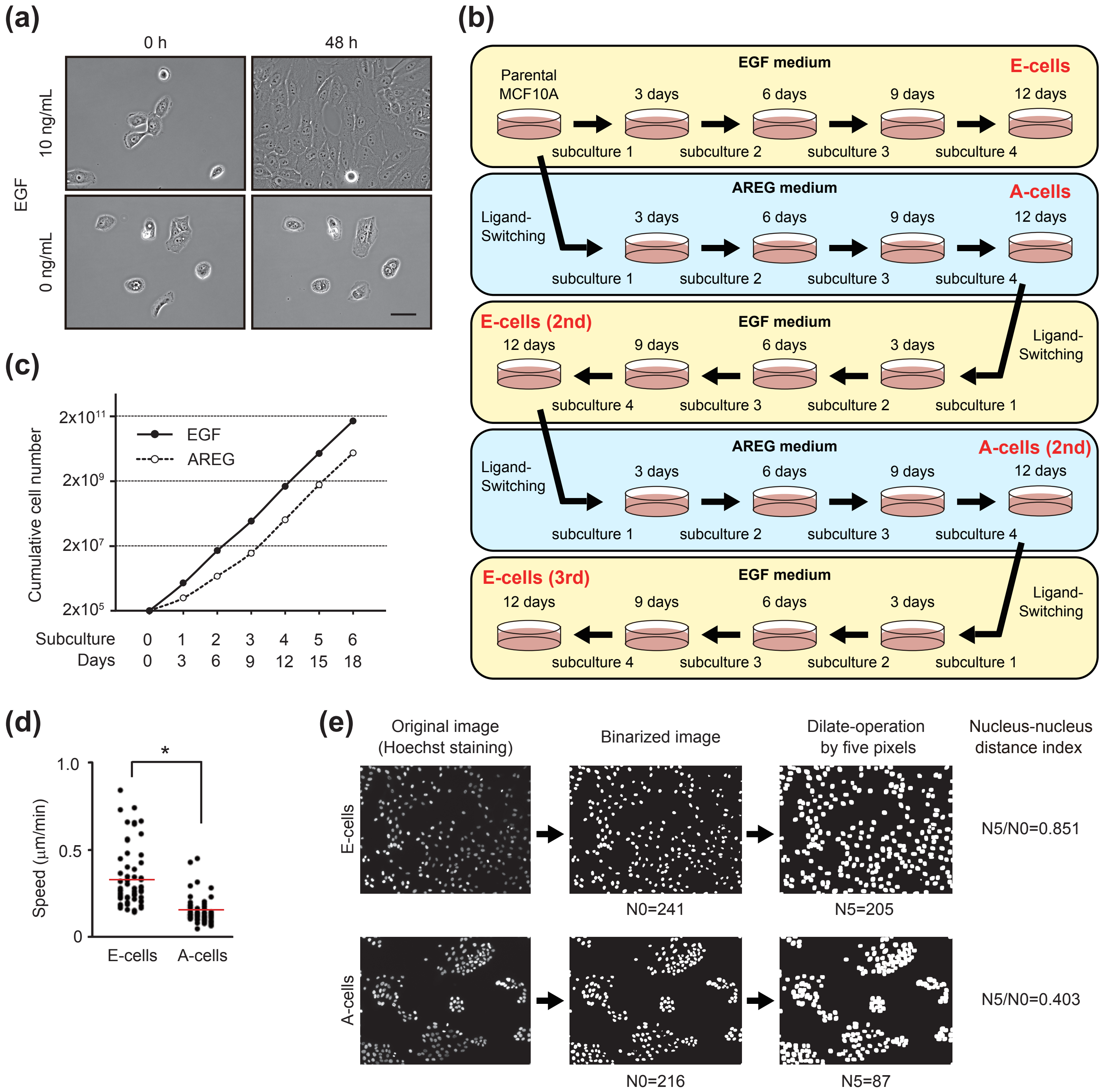

Supplementary Figure 2

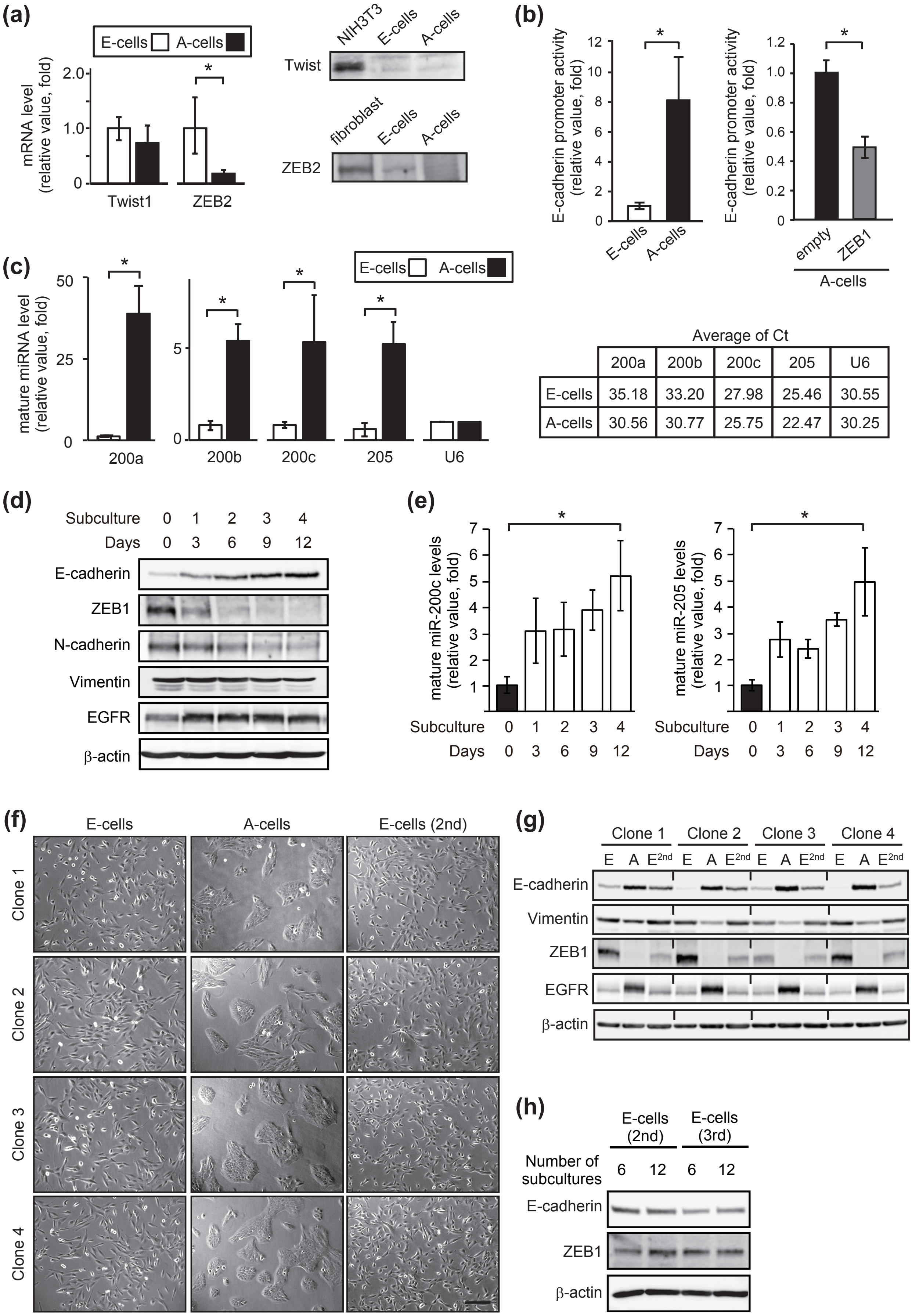

Average of Ct

	200a	200b	200c	205	U6
E-cells	35.18	33.20	27.98	25.46	30.55
A-cells	30.56	30.77	25.75	22.47	30.25

Supplementary Figure 3

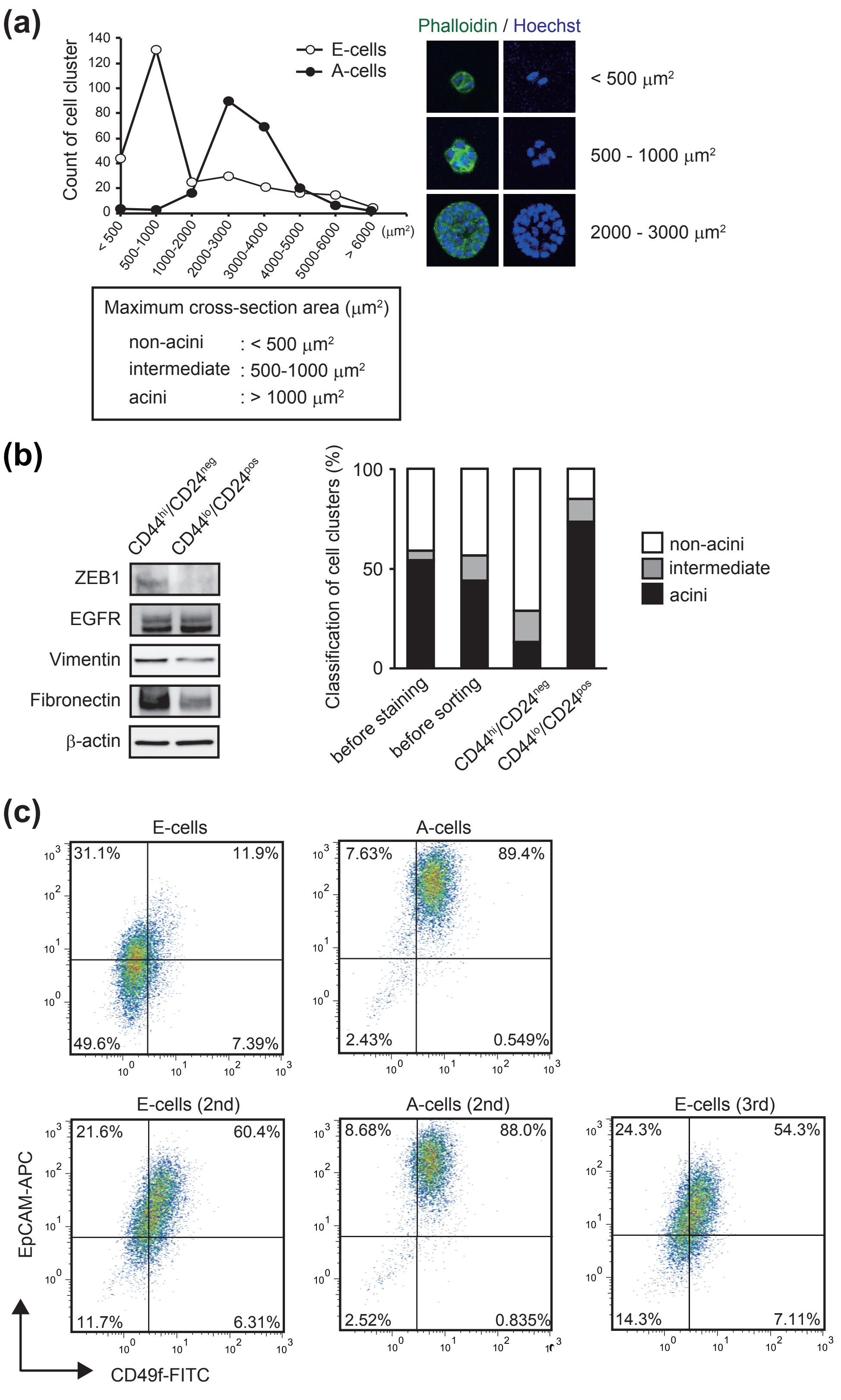

Supplementary Figure 4

(a)

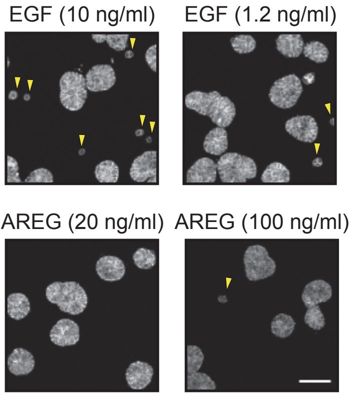

(b)

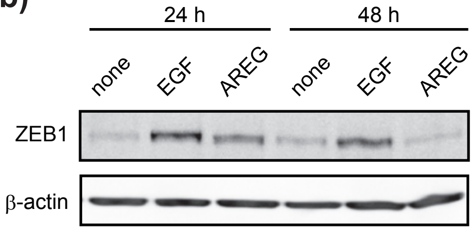

Supplementary Figure 5

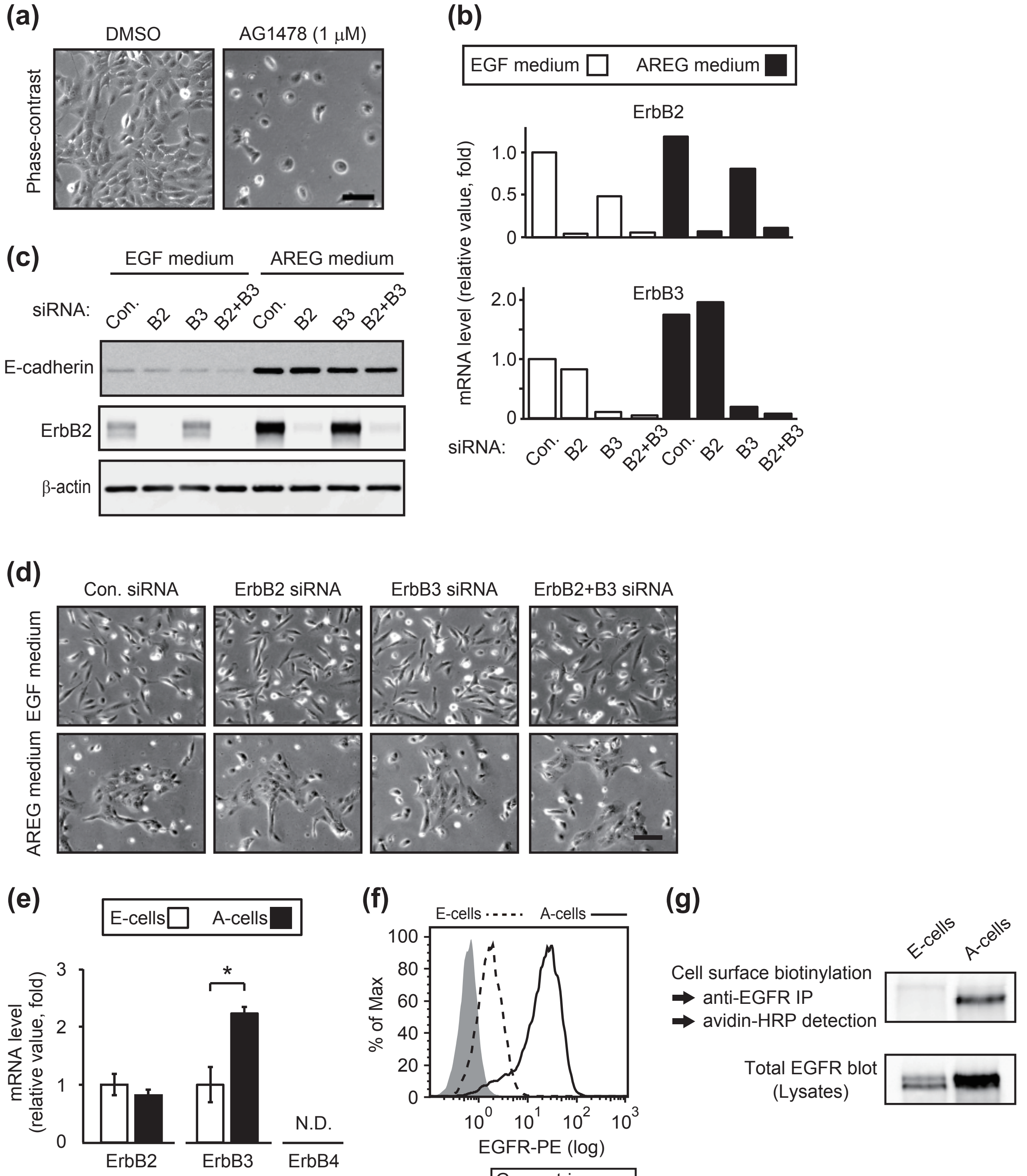

	Geometric mean
control	0.727
E-cells	2.59
A-cells	31.0

Supplementary Figure 6

(a)

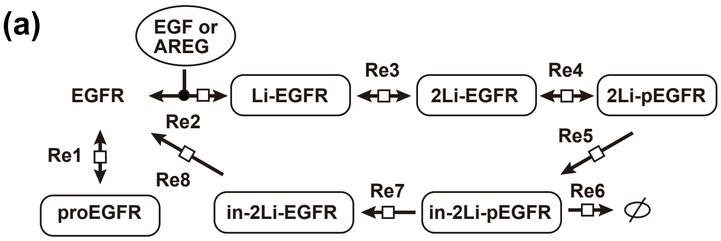

(b)

Re	Reaction name	Reaction	k1 (/min)	k2 (/min)
1	EGFR synthesis	proEGFR $\xleftrightarrow{k1}$ EGFR	0.0000002	
2	Ligand + EGFR	EGF + EGFR $\underset{k2}{\overset{k1}{\rightleftharpoons}}$ EGF-EGFR	46139	1042.0
		AREG + EGFR $\underset{k2}{\overset{k1}{\rightleftharpoons}}$ AREG-EGFR	469.91	1042.0
3	Li-EGFR + Li-EGFR	Li-EGFR + Li-EGFR $\underset{k2}{\overset{k1}{\rightleftharpoons}}$ 2Li-EGFR	0.01295	0.000011
4	2Li-EGFR phosphorylation	2Li-EGFR $\underset{k2}{\overset{k1}{\rightleftharpoons}}$ 2Li-pEGFR	565113	3.5697
5	2Li-pEGFR internalization	2Li-pEGFR $\xrightarrow{k1}$ in-2Li-pEGFR	0.94055	
6	in-2Li-pEGFR degradation	in-2Li-pEGFR $\xrightarrow{k1}$ $\varnothing$	1128.4	
7	in-2Li-pEGFR dephosphorylation	in-2Li-pEGFR $\xrightarrow{k1}$ in-2Li-EGFR	535.30	
8	in-2Li-EGFR recycling	in-2Li-EGFR $\xrightarrow{k1}$ EGFR	0.000264	

(c)

name	initial amounts
proEGFR	0.29519 (E-cell, fixed)
	0.99884 (A-cell, fixed)
EGFR	0.29519 (E-cell)
	0.99884 (A-cell)
Li-EGFR	0
2Li-EGFR	0
2Li-pEGFR	0
in-2Li-pEGFR	0
in-2Li-EGFR	0
Ligands	1 (fixed)

(d)

name	scale factor
pEGFR	45.9468

(e)

Differential equation	Re
$\frac{d[EGFR]}{dt}$ = k1(synthesis)·([proEGFR]−[EGFR])	1
-k1(Li+EGFR)·[Li]·[EGFR]+k2(LiEGFR-dess)·[Li-EGFR]	2
+2·k1(recycle)·[in-2Li-EGFR]	8
$\frac{d[Li\text{-}EGFR]}{dt}$ = k1(Li+EGFR)·[Li]·[EGFR]-k2(LiEGFR-dess)·[Li-EGFR]	2
-2·k1(2LiEGFR-syns)·[Li-EGFR]·[Li-EGFR]	3
+2·k2(2LiEGFR-dess)·[2Li-EGFR]	
$\frac{d[2Li\text{-}EGFR]}{dt}$ = k1(2LiEGFR-syns)·[Li-EGFR]·[Li-EGFR]	3
-k2(2LiEGFR-dess)·[2Li-EGFR]	
-k1(2LiEGFR-phos)·[2Li-EGFR]	4
+k2(2LiEGFR-dephos)·[2Li-pEGFR]	
$\frac{d[2Li\text{-}pEGFR]}{dt}$ = k1(2LiEGFR-phos)·[2Li-EGFR]	4
-k2(2LiEGFR-dephos)·[2Li-pEGFR]	
-k1(2LiEGFR-inter)·[2Li-pEGFR]	5
$\frac{d[in\text{-}2Li\text{-}pEGFR]}{dt}$ = k1(2LiEGFR-inter)·[2Li-pEGFR]	5
-k1(2LiEGFR-deg)·[in-2Li-pEGFR]	6
-k1(2LiEGFR-dephos)·[in-2Li-pEGFR]	7
$\frac{d[in\text{-}2Li\text{-}EGFR]}{dt}$ = k1(2LiEGFR-dephos)·[in-2Li-pEGFR]	7
-k1(recycle)·[in-2Li-EGFR]	8

Supplementary Figure 7

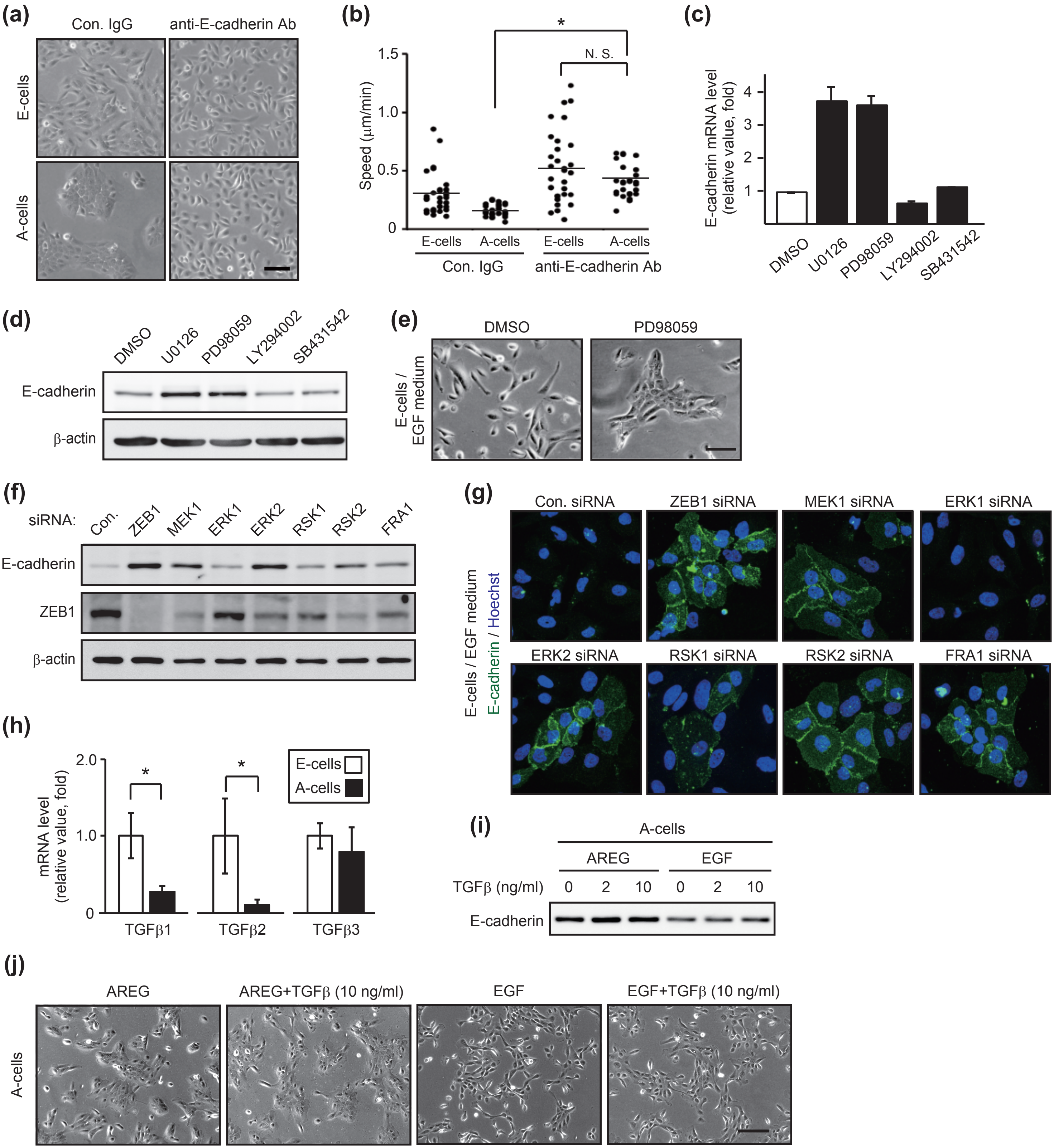

Supplementary Figure 8

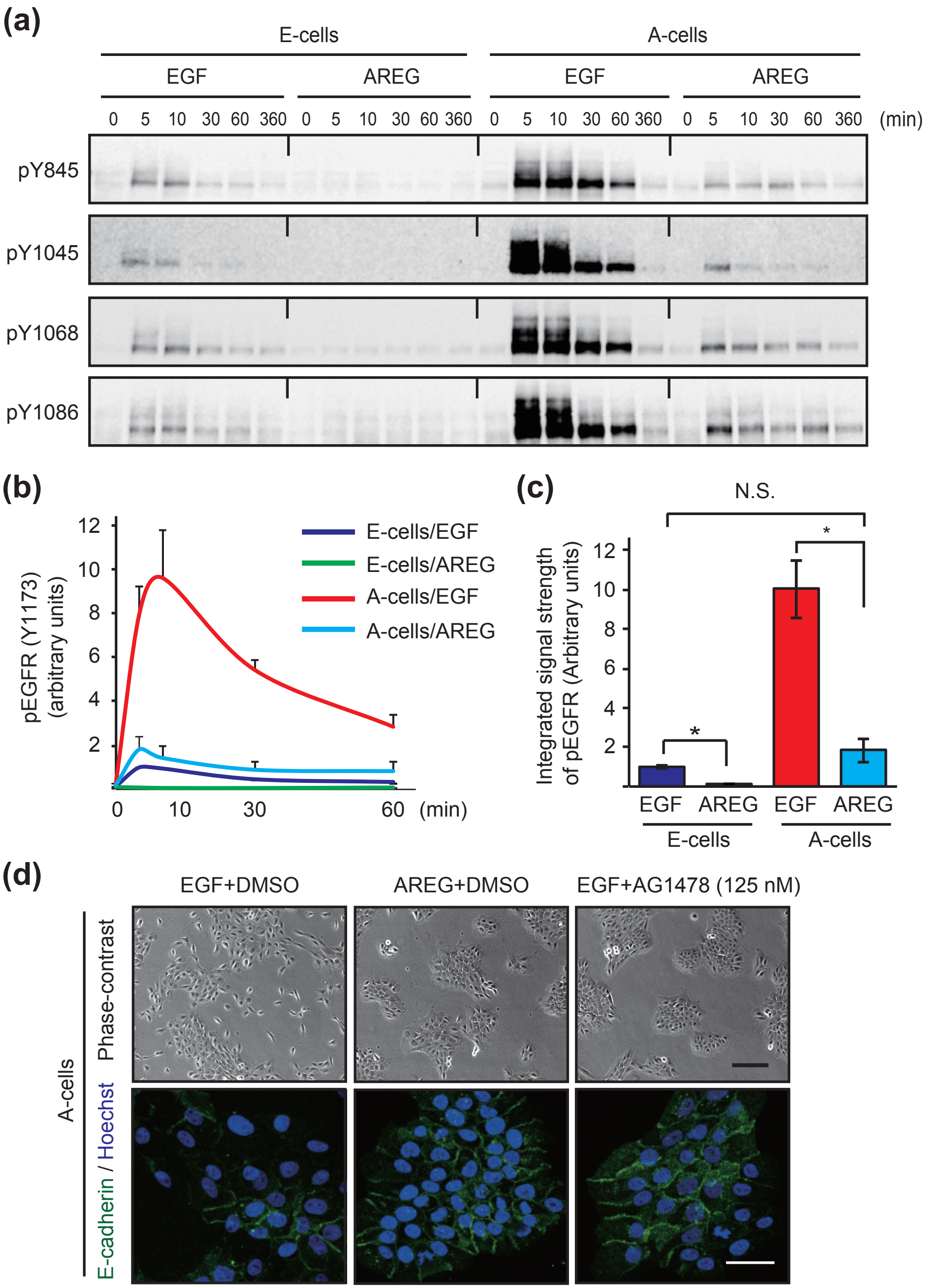

Supplementary Figure 9

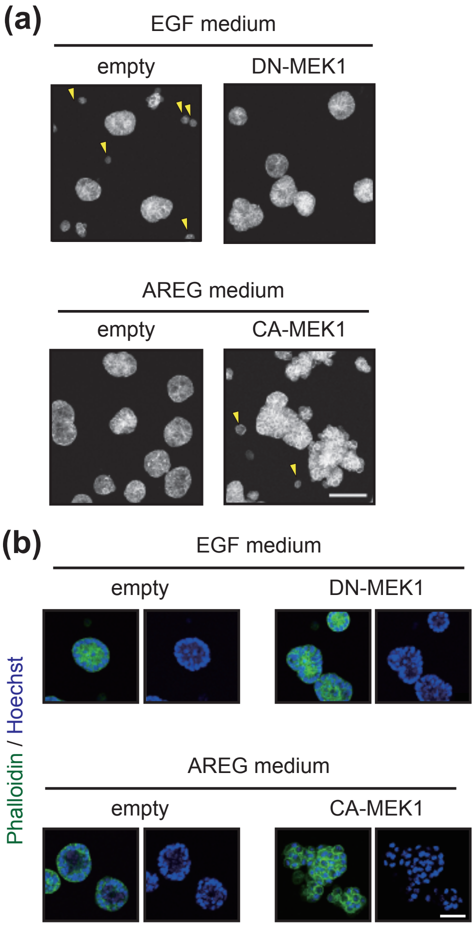

Supplementary Figure 10

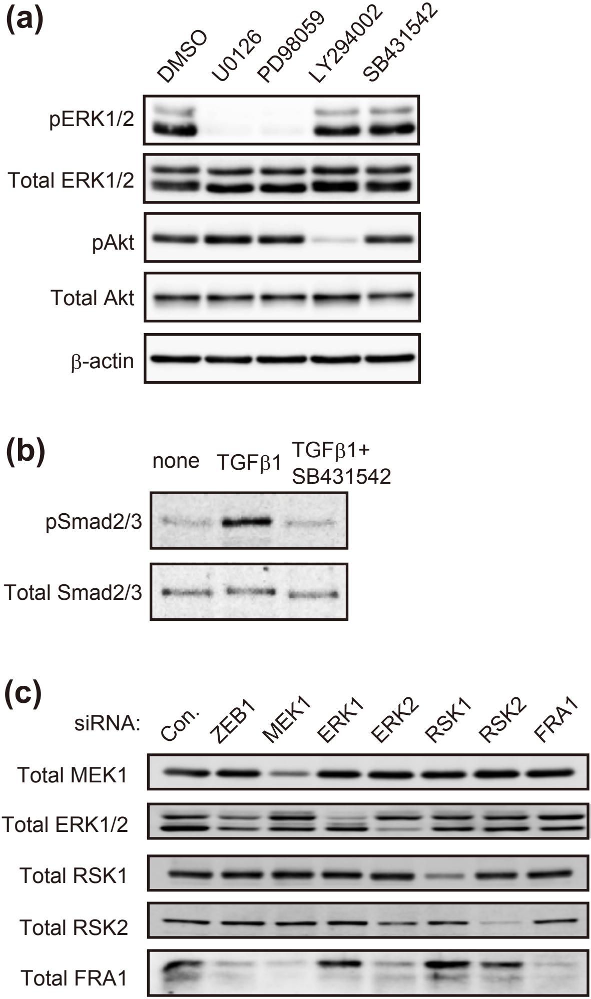

Supplementary Table 1. Differently expressed genes between E-cells and A-cells

Probe Set ID	Entrez Gene	Gene Symbol	Unigene(Avadis)	Gene Title	Fold change (A-cell/E-cell)	A-cell (normalized)	E-cell (normalized)
205064_at	6699	SPRR1B	Hs.1076	small proline-rich protein 1B	113.06023	3.4104738	-3.4104736
204734_at	3866	KRT15	Hs.654570	keratin 15	105.115234	3.357914	-3.357914
202489_s_at	5349	FXYD3	Hs.301350	FXYD domain containing ion transport re	97.27018	3.3019629	-3.3019629
201131_s_at	999	CDH1	Hs.461086	cadherin 1, type 1, E-cadherin (epithelial)	96.99502	3.2999191	-3.2999196
219995_s_at	79755	ZNF750	Hs.653124	zinc finger protein 750	79.05819	3.1524215	-3.1524215
206165_s_at	9635	CLCA2	Hs.241551	chloride channel accessory 2	72.03645	3.085328	-3.0853271
219476_at	79098	C1orf116	Hs.32417	chromosome 1 open reading frame 116	70.63181	3.0711231	-3.0711231
217528_at	9635	CLCA2	Hs.241551	chloride channel accessory 2	70.23337	3.0670424	-3.0670424
204614_at	5055	SERPINB2	Hs.594481	serpin peptidase inhibitor, clade B (ovalbu	68.56806	3.0497322	-3.0497327
235075_at	1830	DSG3	Hs.1925	desmoglein 3	59.988926	2.953312	-2.9533124
209863_s_at	8626	TP63	Hs.137569	tumor protein p63	59.960205	2.9529667	-2.9529667
206164_at	9635	CLCA2	Hs.241551	chloride channel accessory 2	54.79605	2.888	-2.888
226755_at	642587	LOC642587	Hs.510543	NPC-A-5	52.75201	2.860577	-2.860577
209351_at	3861	KRT14	Hs.654380	keratin 14	45.420506	2.752636	-2.752636
206166_s_at	9635	CLCA2	Hs.241551	chloride channel accessory 2	40.97192	2.6782818	-2.6782818
217272_s_at	5275	SERPINB13	Hs.241407	serpin peptidase inhibitor, clade B (ovalbu	37.369175	2.6118884	-2.6118884
225846_at	54845	ESRP1	Hs.487471	epithelial splicing regulatory protein 1	36.235104	2.5896578	-2.5896583
205363_at	8424	BBOX1	Hs.591996	butyrobetaine (gamma), 2-oxoglutarate di	35.89724	2.5829005	-2.5829003
209956_s_at	816	CAMK2B	Hs.351887	calcium/calmodulin-dependent protein kir	34.55651	2.5554428	-2.5554428
213680_at	3854	KRT6B	Hs.708950	keratin 6B	33.229218	2.5271902	-2.5271902
208650_s_at	100133941	CD24	Hs.644105	CD24 molecule	32.85841	2.5190954	-2.5190954
266_s_at	100133941	CD24	Hs.644105	CD24 molecule	32.418415	2.5093708	-2.5093708
211361_s_at	5275	SERPINB13	Hs.241407	serpin peptidase inhibitor, clade B (ovalbu	31.365746	2.485559	-2.4855592
227492_at	4950	OCLN	Hs.592605	occludin	29.598793	2.4437332	-2.4437332
219909_at	79148	MMP28	Hs.380710	matrix metallopeptidase 28	28.982853	2.428564	-2.4285636
239430_at	374918	IGFL1	Hs.546554	IGF-like family member 1	28.296389	2.411273	-2.411273
216379_x_at	100133941	CD24	Hs.644105	CD24 molecule	27.436884	2.3890219	-2.3890228
203780_at	10205	MPZL2	Hs.116651	myelin protein zero-like 2	27.098757	2.3800774	-2.3800774
239381_at	5650	KLK7	Hs.151254	kallikrein-related peptidase 7	27.06459	2.3791676	-2.379167
202935_s_at	6662	SOX9	Hs.647409	SRY (sex determining region Y)-box 9	26.986841	2.3770924	-2.377092
205157_s_at	3872	KRT17	Hs.2785	keratin 17	25.99094	2.349969	-2.349968
226701_at	2702	GJA5	Hs.447968	gap junction protein, alpha 5, 40kDa	25.14329	2.3260508	-2.326051
213796_at	6698	SPRR1A	Hs.46320	small proline-rich protein 1A	24.859955	2.3178759	-2.3178759
208651_x_at	100133941	CD24	Hs.644105	CD24 molecule	24.151915	2.2970324	-2.2970333
228865_at	79098	C1orf116	Hs.32417	chromosome 1 open reading frame 116	23.507147	2.2775135	-2.277514
239273_s_at	79148	MMP28	Hs.380710	matrix metallopeptidase 28	22.750011	2.2538977	-2.2538977
207935_s_at	3860	KRT13	Hs.654550	keratin 13	22.342386	2.2408557	-2.240856
219127_at	79170	PRR15L	Hs.368260	proline rich 15-like	22.327822	2.240385	-2.2403855
41469_at	5266	PI3	Hs.112341	peptidase inhibitor 3, skin-derived	22.283476	2.2389512	-2.2389512
209771_x_at	100133941	CD24	Hs.644105	CD24 molecule	22.17811	2.2355318	-2.2355328

218677_at	57402	S100A14	Hs.288998	S100 calcium binding protein A14	22.04266	2.2311134	-2.231113
209925_at	4950	OCLN	Hs.592605	occludin	21.86333	2.2252207	-2.2252207
205595_at	1830	DSG3	Hs.1925	desmoglein 3	21.691313	2.2195225	-2.219523
1558846_at	119548	PNLIPRP3	Hs.276724	pancreatic lipase-related protein 3	21.54901	2.214775	-2.2147746
204379_s_at	2261	FGFR3	Hs.1420	fibroblast growth factor receptor 3	21.50425	2.213275	-2.213275
209772_s_at	100133941	CD24	Hs.644105	CD24 molecule	20.990026	2.195816	-2.195816
1552477_a_at	3664	IRF6	Hs.724724	interferon regulatory factor 6	20.92995	2.1937485	-2.1937485
202488_s_at	5349	FXYD3	Hs.301350	FXYD domain containing ion transport re	20.775105	2.1883917	-2.1883922
227949_at	116154	PHACTR3	Hs.473218	phosphatase and actin regulator 3	19.265543	2.1339755	-2.1339755
220979_s_at	81849	ST6GALNAC5	Hs.303609	ST6 (alpha-N-acetyl-neuraminyl-2,3-beta-	18.550995	2.1067123	-2.1067126
1555942_a_at	642587	LOC642587	Hs.510543	NPC-A-5	18.321516	2.0977335	-2.0977335
212236_x_at	3872	KRT17	Hs.2785	keratin 17	18.269632	2.0956879	-2.0956879
232056_at	8796	SCEL	Hs.534699	sciellin	18.1791	2.0921044	-2.0921047
202936_s_at	6662	SOX9	Hs.647409	SRY (sex determining region Y)-box 9	18.088142	2.0884862	-2.0884862
215465_at	26154	ABCA12	Hs.134585	ATP-binding cassette, sub-family A (ABC	18.012173	2.0854502	-2.0854502
233011_at	301	ANXA1	Hs.494173	Annexin A1	17.778233	2.0760202	-2.0760198
203691_at	5266	PI3	Hs.112341	peptidase inhibitor 3, skin-derived	17.608765	2.0691109	-2.0691109
212657_s_at	3557	IL1RN	Hs.81134	interleukin 1 receptor antagonist	17.327919	2.0575132	-2.0575132
209301_at	760	CA2	Hs.155097	carbonic anhydrase II	17.013138	2.0442886	-2.0442886
239272_at	79148	MMP28	Hs.380710	matrix metallopeptidase 28	16.978327	2.0428114	-2.042811
229385_s_at	257000	PLAC2	Hs.515575	placenta-specific 2 (non-protein coding)	16.917315	2.0402145	-2.040214
202790_at	1366	CLDN7	Hs.513915	claudin 7	16.283426	2.0126657	-2.0126667
208607_s_at	6288 /// 6289	SAA1 /// SAA2	Hs.1955	serum amyloid A1 /// serum amyloid A2	15.975038	1.9988737	-1.9988737
222830_at	29841	GRHL1	Hs.418493	grainyhead-like 1 (Drosophila)	15.973158	1.9987888	-1.9987888
206884_s_at	8796	SCEL	Hs.534699	sciellin	15.73711	1.9880495	-1.9880493
202481_at	9249	DHRS3	Hs.289347	dehydrogenase/reductase (SDR family) m	15.336386	1.9694433	-1.9694433
202597_at	3664	IRF6	Hs.724724	interferon regulatory factor 6	14.692777	1.9385176	-1.9385176
230356_at			Hs.290255		14.510425	1.9295092	-1.9295087
203779_s_at	10205	MPZL2	Hs.116651	myelin protein zero-like 2	14.385869	1.9232903	-1.9232903
205778_at	5650	KLK7	Hs.151254	kallikrein-related peptidase 7	14.186403	1.9132185	-1.9132185
235146_at	57458	TMCC3	Hs.370410	transmembrane and coiled-coil domain fa	14.132443	1.9104695	-1.9104695
209114_at	10103	TSPAN1	Hs.38972	tetraspanin 1	14.03453	1.9054546	-1.9054542
226847_at	10468	FST	Hs.9914	follistatin	13.957185	1.9014683	-1.9014678
241994_at	7498	XDH	Hs.250	xanthine dehydrogenase	13.932061	1.9001684	-1.9001684
226899_at	219699	UNC5B	Hs.522997	unc-5 homolog B (C. elegans)	13.607411	1.8831606	-1.8831601
1554997_a_at	5743	PTGS2	Hs.196384	prostaglandin-endoperoxide synthase 2 (p	13.452795	1.874917	-1.874917
208190_s_at	51599	LSR	Hs.466507	lipolysis stimulated lipoprotein receptor	13.219918	1.8623209	-1.8623204
211122_s_at	6373	CXCL11	Hs.632592	chemokine (C-X-C motif) ligand 11	13.132793	1.8575511	-1.8575509
209309_at	563	AZGP1	Hs.546239	alpha-2-glycoprotein 1, zinc-binding	12.585568	1.8268495	-1.826849
222168_at			Hs.675520		12.074377	1.7969384	-1.7969384
200953_s_at	894	CCND2	Hs.376071	cyclin D2	12.005017	1.7927828	-1.7927828
238967_at			Hs.686016		11.953605	1.7896872	-1.7896867
239155_at	1525	CXADR	Hs.634837	coxsackie virus and adenovirus receptor	11.814796	1.7812614	-1.7812614

232116_at	57822	GRHL3	Hs.657920	grainyhead-like 3 (Drosophila)	11.80143	1.7804451	-1.7804446
210118_s_at	3552	IL1A	Hs.1722	interleukin 1, alpha	11.713887	1.775074	-1.775074
204748_at	5743	PTGS2	Hs.196384	prostaglandin-endoperoxide synthase 2 (p	11.698767	1.7741423	-1.7741423
204469_at	5803	PTPRZ1	Hs.489824	protein tyrosine phosphatase, receptor-typ	11.224713	1.7443032	-1.7443035
229764_at	285386	TPRG1	Hs.338851	tumor protein p63 regulated 1	11.150573	1.7395229	-1.7395229
224650_at	114569	MAL2	Hs.201083	mal, T-cell differentiation protein 2	11.129673	1.7381697	-1.7381697
219121_s_at	54845	ESRP1	Hs.487471	epithelial splicing regulatory protein 1	11.112258	1.73704	-1.73704
232202_at			Hs.152423		11.009635	1.7303476	-1.7303472
203180_at	220	ALDH1A3	Hs.459538	aldehyde dehydrogenase 1 family, membe	10.962884	1.7272778	-1.7272778
203797_at	7447	VSNL1	Hs.444212	visinin-like 1	10.714766	1.7107639	-1.7107644
239370_at	100505633	LOC100505633		hypothetical LOC100505633	10.6250105	1.7046962	-1.7046962
208153_s_at	2196	FAT2	Hs.591255	FAT tumor suppressor homolog 2 (Droso	10.605037	1.7033386	-1.7033391
203638_s_at	2263	FGFR2	Hs.533683	fibroblast growth factor receptor 2	10.488574	1.695373	-1.6953735
202917_s_at	6279	S100A8	Hs.416073	S100 calcium binding protein A8	10.47337	1.6943274	-1.6943264
229296_at	100506119 /// 10	LOC100506119 /// LOC100508342		hypothetical LOC100506119 /// hypotheti	10.387902	1.6884165	-1.688416
236279_at			Hs.659129		10.310342	1.6830101	-1.6830101
214456_x_at	6288 /// 6289	SAA1 /// SAA2	Hs.1955	serum amyloid A1 /// serum amyloid A2	10.288786	1.6815004	-1.6815004
235957_at	23426	GRIP1	Hs.505946	glutamate receptor interacting protein 1	10.257629	1.6793127	-1.6793127
229147_at	166824	RASSF6	Hs.590920	Ras association (RalGDS/AF-6) domain f	9.813721	1.6473999	-1.6474004
201839_s_at	4072	EPCAM	Hs.542050	epithelial cell adhesion molecule	9.760563	1.6434822	-1.6434822
231928_at	54626	HES2	Hs.118727	hairy and enhancer of split 2 (Drosophila)	9.740566	1.642003	-1.6420026
229616_s_at	196996	GRAMD2	Hs.596332	GRAM domain containing 2	9.712218	1.6399002	-1.6399007
206515_at	4051	CYP4F3	Hs.106242	cytochrome P450, family 4, subfamily F,	9.655054	1.635642	-1.6356423
230518_at	10205	MPZL2	Hs.116651	myelin protein zero-like 2	9.610107	1.6322765	-1.632276
237086_at	3169	FOXA1	Hs.163484	Forkhead box A1	9.553587	1.6280212	-1.6280212
210404_x_at	816	CAMK2B	Hs.351887	calcium/calmodulin-dependent protein kir	9.494279	1.6235294	-1.623529
203798_s_at	7447	VSNL1	Hs.444212	visinin-like 1	9.484126	1.6227574	-1.6227574
212841_s_at	8495	PPFIBP2	Hs.655714	PTPRF interacting protein, binding protei	9.017077	1.5863299	-1.5863299
205916_at	6278	S100A7	Hs.112408	S100 calcium binding protein A7	8.975734	1.583015	-1.583015
207655_s_at	29760	BLNK	Hs.665244	B-cell linker	8.861427	1.5737696	-1.5737696
235182_at	140862	ISM1	Hs.559353	isthmin 1 homolog (zebrafish)	8.472448	1.5413895	-1.5413895
218182_s_at	9076	CLDN1	Hs.439060	claudin 1	8.402713	1.5354276	-1.5354276
202411_at	3429	IFI27	Hs.532634	interferon, alpha-inducible protein 27	8.3442335	1.5303898	-1.5303898
243386_at	54897	CASZ1	Hs.439894	castor zinc finger 1	8.33387	1.5294933	-1.5294933
209800_at	3868	KRT16	Hs.655160	keratin 16	8.314394	1.5278053	-1.5278058
222549_at	9076	CLDN1	Hs.439060	claudin 1	8.051525	1.504631	-1.504631
203535_at	6280	S100A9	Hs.112405	S100 calcium binding protein A9	7.9324694	1.493885	-1.493885
228320_x_at	92558	CCDC64	Hs.369763	coiled-coil domain containing 64	7.925119	1.4932165	-1.493216
204584_at	3897	L1CAM	Hs.522818	L1 cell adhesion molecule	7.8959427	1.4905558	-1.4905558
209792_s_at	5655	KLK10	Hs.275464	kallikrein-related peptidase 10	7.837589	1.4852052	-1.4852047
221872_at	5918	RARRES1	Hs.131269	retinoic acid receptor responder (tazaroter	7.8117967	1.4828272	-1.4828272
1552478_a_at	3664	IRF6	Hs.724724	interferon regulatory factor 6	7.7943363	1.4812131	-1.4812131
232060_at	4919	ROR1	Hs.654491	receptor tyrosine kinase-like orphan recep	7.748477	1.4769564	-1.4769564

231022_at	4950	OCLN	Hs.592605	occludin	7.7286544	1.4751086	-1.4751086
205870_at	624	BDKRB2	Hs.724899	bradykinin receptor B2	7.6752954	1.4701109	-1.4701114
219225_at	79605	PGBD5	Hs.520463	piggyBac transposable element derived 5	7.655703	1.4682674	-1.4682674
219856_at	79098	C1orf116	Hs.32417	chromosome 1 open reading frame 116	7.5649877	1.4596691	-1.4596686
213839_at	79789	CLMN	Hs.301478	calmin (calponin-like, transmembrane)	7.432681	1.4469414	-1.4469414
232164_s_at	83481	EPPK1	Hs.200412	epiplakin 1	7.3545804	1.4393215	-1.4393215
203821_at	1839	HBEGF	Hs.799	heparin-binding EGF-like growth factor	7.307786	1.4347172	-1.4347172
204533_at	3627	CXCL10	Hs.632586	chemokine (C-X-C motif) ligand 10	7.276357	1.4316082	-1.4316082
202712_s_at	1159 /// 548596	CKMT1A /// CK	Hs.425633	creatine kinase, mitochondrial 1A /// creat	7.107085	1.414629	-1.414629
225016_at	147495	APCDD1	Hs.293274	adenomatosis polyposis coli down-regulat	7.1049128	1.4144082	-1.4144087
201147_s_at	7078	TIMP3	Hs.644633	TIMP metallopeptidase inhibitor 3	7.097545	1.41366	-1.41366
223541_at	3038	HAS3	Hs.592069	hyaluronan synthase 3	7.0627804	1.4101181	-1.4101181
225759_x_at	79789	CLMN	Hs.301478	calmin (calponin-like, transmembrane)	7.0459323	1.4083953	-1.4083953
203256_at	1001	CDH3	Hs.191842	cadherin 3, type 1, P-cadherin (placental)	6.967039	1.4002733	-1.4002724
232165_at	83481	EPPK1	Hs.200412	epiplakin 1	6.9609213	1.3996391	-1.3996391
202890_at	9053	MAP7	Hs.486548	microtubule-associated protein 7	6.9006495	1.3933659	-1.3933663
210147_at	419	ART3	Hs.24976	ADP-ribosyltransferase 3	6.899147	1.393209	-1.393209
204971_at	1475	CSTA	Hs.518198	cystatin A (stefin A)	6.8683906	1.389986	-1.389986
218736_s_at	54873	PALMD	Hs.483993	palmdelphin	6.77121	1.3797069	-1.3797069
204455_at	667	DST	Hs.604915	dystonin	6.705918	1.3727179	-1.3727169
206683_at	7718	ZNF165	Hs.535177	zinc finger protein 165	6.6385818	1.3654375	-1.3654375
204351_at	6286	S100P	Hs.2962	S100 calcium binding protein P	6.5557246	1.3563776	-1.3563776
206008_at	7051	TGM1	Hs.508950	transglutaminase 1 (K polypeptide epideri	6.5176826	1.3521795	-1.3521795
205819_at	8685	MARCO	Hs.67726	macrophage receptor with collagenous str	6.4509015	1.3447504	-1.3447504
205014_at	9982	FGFBP1	Hs.1690	fibroblast growth factor binding protein 1	6.4338884	1.342845	-1.3428459
236262_at	79812	MMRN2	Hs.524479	multimerin 2	6.4238195	1.3417158	-1.3417156
203394_s_at	3280	HES1	Hs.250666	hairy and enhancer of split 1, (Drosophila	6.4152446	1.3407526	-1.3407516
203904_x_at	3732	CD82	Hs.527778	CD82 molecule	6.4056463	1.3396721	-1.3396721
239089_at			Hs.714938		6.3928757	1.3382325	-1.3382325
202525_at	5652	PRSS8	Hs.75799	protease, serine, 8	6.3378596	1.3319979	-1.3319979
232082_x_at	6707	SPRR3	Hs.139322	small proline-rich protein 3	6.336984	1.3318982	-1.3318982
232914_s_at	54843	SYTL2	Hs.369520	synaptotagmin-like 2	6.322898	1.3302932	-1.3302927
219836_at	79413	ZBED2	Hs.136912	zinc finger, BED-type containing 2	6.28768	1.3262639	-1.3262639
238439_at	118932	ANKRD22	Hs.217484	ankyrin repeat domain 22	6.269	1.3241177	-1.3241177
228910_at			Hs.634153		6.2167387	1.318079	-1.318079
202037_s_at	6422	SFRP1	Hs.213424	secreted frizzled-related protein 1	6.2030735	1.3164921	-1.3164911
209719_x_at	6317	SERPINB3	Hs.227948	serpin peptidase inhibitor, clade B (ovalbu	6.1424804	1.3094106	-1.3094108
226374_at	1525	CXADR	Hs.634837	coxsackie virus and adenovirus receptor	6.1177487	1.3065004	-1.3065004
220289_s_at	55057	AIM1L	Hs.128738	absent in melanoma 1-like	6.0970097	1.3040509	-1.3040509
223484_at	84419	C15orf48	Hs.112242	chromosome 15 open reading frame 48	6.0464025	1.2980385	-1.2980385
228570_at	121551	BTBD11	Hs.271272	BTB (POZ) domain containing 11	6.011296	1.293838	-1.293838
210163_at	6373	CXCL11	Hs.632592	chemokine (C-X-C motif) ligand 11	5.9699774	1.2888627	-1.2888627
205768_s_at	11001	SLC27A2	Hs.720807	solute carrier family 27 (fatty acid transpc	5.962874	1.2880039	-1.2880039

203642_s_at	22837	COBLL1	Hs.470457	COBL-like 1	5.8788633	1.2777691	-1.2777681
209794_at	9901	SRGAP3	Hs.654743	SLIT-ROBO Rho GTPase activating prot	5.8473015	1.2738857	-1.2738853
1570585_at	196264	MPZL3	Hs.15396	myelin protein zero-like 3	5.827899	1.2714882	-1.2714877
241990_at	171177	RHOV	Hs.447901	ras homolog gene family, member V	5.819176	1.2704077	-1.2704072
203021_at	6590	SLPI	Hs.517070	secretory leukocyte peptidase inhibitor	5.8180933	1.2702732	-1.2702732
239148_at	91862	MARVELD3	Hs.513706	MARVEL domain containing 3	5.7904296	1.2668352	-1.2668352
213050_at	23242	COBL	Hs.99141	cordon-bleu homolog (mouse)	5.772834	1.2646399	-1.2646399
200951_s_at	894	CCND2	Hs.376071	cyclin D2	5.6981373	1.2552452	-1.2552452
1555551_at	5268	SERPINB5	Hs.55279	serpin peptidase inhibitor, clade B (ovalbu	5.667059	1.2512999	-1.2513003
229242_at			Hs.23349		5.6661167	1.2511802	-1.2511802
218261_at	10053	AP1M2	Hs.18894	adaptor-related protein complex 1, mu 2 s	5.665109	1.2510519	-1.2510519
227235_at	2982	GUCY1A3	Hs.24258	guanylate cyclase 1, soluble, alpha 3	5.6512938	1.2492905	-1.2492907
206392_s_at	5918	RARRES1	Hs.131269	retinoic acid receptor responder (tazaroter	5.627615	1.2462621	-1.2462616
204681_s_at	9771	RAPGEF5	Hs.174768	Rap guanine nucleotide exchange factor (	5.5984607	1.2425151	-1.2425151
209735_at	9429	ABCG2	Hs.480218	ATP-binding cassette, sub-family G (WH	5.590615	1.2415032	-1.2415037
219010_at	55765	C1orf106	Hs.518997	chromosome 1 open reading frame 106	5.589634	1.2413769	-1.2413769
224724_at	55959	SULF2	Hs.162016	sulfatase 2	5.5789657	1.2399988	-1.2399988
235955_at	153562	MARVELD2	Hs.724697	MARVEL domain containing 2	5.556535	1.2370925	-1.237093
203726_s_at	3909	LAMA3	Hs.436367	laminin, alpha 3	5.49175	1.2286329	-1.2286329
203917_at	1525	CXADR	Hs.634837	coxsackie virus and adenovirus receptor	5.485703	1.2278385	-1.227838
202036_s_at	6422	SFRP1	Hs.213424	secreted frizzled-related protein 1	5.4833627	1.2275305	-1.2275305
228933_at	4810	NHS	Hs.201623	Nance-Horan syndrome (congenital catara	5.4745293	1.2263675	-1.2263675
226907_at	81706	PPP1R14C	Hs.486798	protein phosphatase 1, regulatory (inhibito	5.469787	1.2257423	-1.2257423
201150_s_at	7078	TIMP3	Hs.644633	TIMP metallopeptidase inhibitor 3	5.459283	1.2243557	-1.2243557
226225_at	4163	MCC	Hs.593171	mutated in colorectal cancers	5.4297132	1.220438	-1.220438
209260_at	2810	SFN	Hs.523718	stratifin	5.4000826	1.2164907	-1.2164907
235937_at	4950	OCLN	Hs.592605	occludin	5.3680882	1.212204	-1.2122045
65517_at	10053	AP1M2	Hs.18894	adaptor-related protein complex 1, mu 2 s	5.3230457	1.2061262	-1.2061257
222859_s_at	27071	DAPP1	Hs.436271	dual adaptor of phosphotyrosine and 3-ph	5.2842793	1.2008533	-1.2008533
202035_s_at	6422	SFRP1	Hs.213424	secreted frizzled-related protein 1	5.2513757	1.1963482	-1.1963472
205769_at	11001	SLC27A2	Hs.720807	solute carrier family 27 (fatty acid transpo	5.2378635	1.1944895	-1.194489
203395_s_at	3280	HES1	Hs.250666	hairy and enhancer of split 1, (Drosophila	5.2310834	1.1935549	-1.1935549
204836_at	2731	GLDC	Hs.584238	glycine dehydrogenase (decarboxylating)	5.2166214	1.1915579	-1.1915579
213478_at	23254	KAZ	Hs.368823	kazrin	5.211658	1.1908712	-1.1908712
215125_s_at	54575 /// 54576 /	UGT1A1 /// UGT1A10 /// UGT1A3 //		UDP glucuronosyltransferase 1 family, po	5.1853733	1.1872239	-1.1872239
231033_at			Hs.557559		5.1789093	1.1863241	-1.1863241
211483_x_at	816	CAMK2B	Hs.351887	calcium/calmodulin-dependent protein kin	5.176356	1.1859684	-1.1859684
1562440_at	9175	MAP3K13	Hs.656069	Mitogen-activated protein kinase kinase k	5.1673355	1.1847105	-1.18471
219181_at	9388	LIPG	Hs.465102	lipase, endothelial	5.1239247	1.1786251	-1.1786242
224762_at	347735	SERINC2	Hs.270655	serine incorporator 2	5.116157	1.1775303	-1.1775303
231932_at	80342	TRAF3IP3	Hs.147434	TRAF3 interacting protein 3	5.101413	1.1754484	-1.1754484
220030_at	55359	STYK1	Hs.24979	serine/threonine/tyrosine kinase 1	5.0809107	1.1725435	-1.1725435
226535_at	3694	ITGB6	Hs.470399	integrin, beta 6	5.0740247	1.171565	-1.1715655

206125_s_at	11202	KLK8	Hs.104570	kallikrein-related peptidase 8	5.067373	1.170619	-1.170619
219987_at	100288413	LOC100288413	Hs.363087	Similar to Uncharacterized protein LP905	5.057904	1.1692696	-1.16927
216905_s_at	6768	ST14	Hs.504315	suppression of tumorigenicity 14 (colon ca	5.045608	1.1675138	-1.1675143
204636_at	1308	COL17A1	Hs.117938	collagen, type XVII, alpha 1	5.018475	1.1636248	-1.1636243
229030_at	388743	CAPN8	Hs.291487	calpain 8	5.016968	1.1634078	-1.1634078
205220_at	8843	GPR109B	Hs.458425	G protein-coupled receptor 109B	4.9844136	1.1587119	-1.1587119
227184_at	5724	PTAFR	Hs.433540	platelet-activating factor receptor	4.9439497	1.152832	-1.152832
236009_at	64065	PERP	Hs.201446	PERP, TP53 apoptosis effector	4.9234734	1.1498384	-1.149838
222877_at			Hs.660596		4.910191	1.1478896	-1.1478896
209758_s_at	8076	MFAP5	Hs.512842	microfibrillar associated protein 5	4.899699	1.1463466	-1.1463466
201148_s_at	7078	TIMP3	Hs.644633	TIMP metallopeptidase inhibitor 3	4.880301	1.1434851	-1.1434851
211778_s_at	58495	OVOL2	Hs.710157	ovo-like 2 (Drosophila)	4.848451	1.138762	-1.138762
91826_at	54869	EPS8L1	Hs.438862	EPS8-like 1	4.8453627	1.1383023	-1.1383023
205709_s_at	1040	CDS1	Hs.654899	CDP-diacylglycerol synthase (phosphatida	4.840167	1.1375284	-1.1375284
230828_at	196996	GRAMD2	Hs.596332	GRAM domain containing 2	4.8377647	1.1371703	-1.1371703
210845_s_at	5329	PLAUR	Hs.466871	plasminogen activator, urokinase receptor	4.827311	1.1356096	-1.1356101
213056_at	23150	FRMD4B	Hs.709671	FERM domain containing 4B	4.824955	1.1352577	-1.1352577
212070_at	9289	GPR56	Hs.513633	G protein-coupled receptor 56	4.81524	1.1338043	-1.1338034
222746_s_at	54836	BSPRY	Hs.614517	B-box and SPRY domain containing	4.80452	1.1321964	-1.132196
225864_at	157638	FAM84B	Hs.724597	family with sequence similarity 84, memb	4.7839856	1.1291065	-1.1291065
1553982_a_at	338382	RAB7B	Hs.534612	RAB7B, member RAS oncogene family	4.7483745	1.1237168	-1.1237168
202286_s_at	4070	TACSTD2	Hs.23582	tumor-associated calcium signal transduce	4.7386947	1.1222448	-1.1222448
201149_s_at	7078	TIMP3	Hs.644633	TIMP metallopeptidase inhibitor 3	4.734017	1.1215324	-1.1215324
222858_s_at	27071	DAPP1	Hs.436271	dual adaptor of phosphotyrosine and 3-ph	4.7155585	1.1187143	-1.1187143
38037_at	1839	HBEGF	Hs.799	heparin-binding EGF-like growth factor	4.703284	1.1168342	-1.1168342
201195_s_at	8140	SLC7A5	Hs.513797	solute carrier family 7 (cationic amino aci	4.666237	1.1111298	-1.1111298
205067_at	3553	IL1B	Hs.126256	interleukin 1, beta	4.65095	1.1087627	-1.1087627
235141_at	153562	MARVELD2	Hs.724697	MARVEL domain containing 2	4.635101	1.1063004	-1.1063004
202283_at	5176	SERPINF1	Hs.532768	serpin peptidase inhibitor, clade F (alpha-	4.633777	1.1060944	-1.1060944
201428_at	1364	CLDN4	Hs.699253	claudin 4	4.6112814	1.1025839	-1.1025839
216918_s_at	667	DST	Hs.604915	dystonin	4.5665855	1.0955582	-1.0955577
220468_at	80117	ARL14	Hs.287702	ADP-ribosylation factor-like 14	4.5493107	1.092824	-1.092824
206204_at	2888	GRB14	Hs.411881	growth factor receptor-bound protein 14	4.5385785	1.0911202	-1.0911202
241482_at					4.5235095	1.0887213	-1.0887213
211002_s_at	23650	TRIM29	Hs.504115	tripartite motif-containing 29	4.508609	1.0863409	-1.0863414
219489_s_at	64359	NXN	Hs.527989	nucleoredoxin	4.503734	1.0855608	-1.0855608
219938_s_at	9050	PSTPIP2	Hs.567384	proline-serine-threonine phosphatase inter	4.503222	1.0854788	-1.0854788
221042_s_at	79789	CLMN	Hs.301478	calmin (calponin-like, transmembrane)	4.4940643	1.0840101	-1.0840106
207102_at	6718	AKR1D1	Hs.201667	aldo-keto reductase family 1, member D1	4.455738	1.0778322	-1.0778322
218807_at	10451	VAV3	Hs.267659	vav 3 guanine nucleotide exchange factor	4.4062843	1.0697813	-1.0697813
203037_s_at	9788	MTSS1	Hs.336994	metastasis suppressor 1	4.3775887	1.0650682	-1.0650682
228977_at	729680	LOC729680	Hs.130652	hypothetical protein LOC729680	4.3745055	1.0645599	-1.0645599
210827_s_at	1999	ELF3	Hs.67928	E74-like factor 3 (ets domain transcriptio	4.3744125	1.0645447	-1.0645447

243276_at	259173	ALS2CL	Hs.517937	ALS2 C-terminal like	4.3711376	1.0640044	-1.0640044
202005_at	6768	ST14	Hs.504315	suppression of tumorigenicity 14 (colon ca	4.3688874	1.063633	-1.063633
206393_at	7136	TNNI2	Hs.523403	troponin I type 2 (skeletal, fast)	4.3584557	1.0619087	-1.0619082
241470_x_at			Hs.247150		4.3409834	1.059011	-1.059011
206109_at	2523	FUT1	Hs.69747	fucosyltransferase 1 (galactoside 2-alpha-	4.339642	1.0587878	-1.0587883
201286_at	6382	SDC1	Hs.224607	syndecan 1	4.3359103	1.0581675	-1.0581675
241355_at	55806	HR	Hs.272367	hairless homolog (mouse)	4.2926717	1.0509377	-1.0509381
224901_at	79966	SCD5	Hs.379191	stearoyl-CoA desaturase 5	4.2741976	1.0478268	-1.0478268
203287_at	3898	LAD1	Hs.519035	ladinin 1	4.2446504	1.0428228	-1.0428228
229144_at	23254	KAZ	Hs.368823	kazrin	4.238175	1.0417213	-1.0417218
228153_at	255488	RNF144B	Hs.148741	ring finger protein 144B	4.2269764	1.039813	-1.039813
235924_at			Hs.561511		4.221307	1.0388451	-1.0388446
202504_at	23650	TRIM29	Hs.504115	tripartite motif-containing 29	4.213307	1.0374765	-1.0374765
240633_at	285489	DOK7	Hs.122110	docking protein 7	4.1974006	1.0347481	-1.0347481
230266_at	338382	RAB7B	Hs.534612	RAB7B, member RAS oncogene family	4.1791	1.0315962	-1.0315962
201998_at	6480	ST6GAL1	Hs.207459	ST6 beta-galactosamide alpha-2,6-sialyltr	4.1682754	1.0297251	-1.0297256
244107_at					4.14847	1.0262899	-1.0262895
232181_at	133522	PPARGC1B	Hs.483816	peroxisome proliferator-activated receptor	4.1451097	1.0257053	-1.0257049
203571_s_at	10974	C10orf116	Hs.642660	chromosome 10 open reading frame 116	4.1310782	1.0232592	-1.0232592
226185_at	1040	CDS1	Hs.654899	CDP-diacylglycerol synthase (phosphatida	4.0949774	1.0169277	-1.0169277
226069_at	144165	PRICKLE1	Hs.720221	prickle homolog 1 (Drosophila)	4.094785	1.0168939	-1.0168939
205780_at	638	BIK	Hs.475055	BCL2-interacting killer (apoptosis-inducin	4.087978	1.0156937	-1.0156937
244829_at	221718	C6orf218	Hs.173337	chromosome 6 open reading frame 218	4.081557	1.0145597	-1.0145597
232277_at	64078	SLC28A3	Hs.591877	solute carrier family 28 (sodium-coupled n	4.06561	1.0117359	-1.0117359
204542_at	10610	ST6GALNAC2	Hs.592105	ST6 (alpha-N-acetyl-neuraminyl-2,3-beta-	4.0593786	1.0106292	-1.0106297
236470_at			Hs.606038		4.048271	1.0086527	-1.0086532
238778_at	143098	MPP7	Hs.499159	membrane protein, palmitoylated 7 (MAG	4.037175	1.0066733	-1.0066729
213230_at	30850	CDR2L	Hs.78358	cerebellar degeneration-related protein 2-l	4.0296764	1.005332	-1.005332
218806_s_at	10451	VAV3	Hs.267659	vav 3 guanine nucleotide exchange factor	3.9808824	0.9965439	-0.99654436
201510_at	1999	ELF3	Hs.67928	E74-like factor 3 (ets domain transcription	3.9692445	0.994432	-0.99443245
212365_at	4430	MYO1B	Hs.439620	myosin IB	3.958283	0.99243736	-0.99243736
207345_at	10468	FST	Hs.9914	follistatin	3.9470842	0.99039364	-0.99039364
39248_at	360	AQP3	Hs.234642	aquaporin 3 (Gill blood group)	3.9418797	0.9894419	-0.9894419
201416_at	6659	SOX4	Hs.643910	SRY (sex determining region Y)-box 4	3.9276521	0.9868336	-0.9868336
214586_at	2861	GPR37	Hs.723816	G protein-coupled receptor 37 (endothelin	3.9208815	0.985589	-0.985589
225613_at	375449	MAST4	Hs.595458	microtubule associated serine/threonine ki	3.9142778	0.9843731	-0.9843731
242127_at			Hs.659830		3.8959165	0.98098135	-0.98098135
220540_at	60598	KCNK15	Hs.528664	potassium channel, subfamily K, member	3.888229	0.97955656	-0.97955656
238750_at	56477	CCL28	Hs.656904	chemokine (C-C motif) ligand 28	3.888144	0.9795408	-0.9795408
206170_at	154	ADRB2	Hs.2551	adrenergic, beta-2-, receptor, surface	3.8876145	0.9794426	-0.9794426
213802_at	8492	PRSS12	Hs.654823	protease, serine, 12 (neurotrypsin, motops	3.8632953	0.974916	-0.974916
229530_at	2982	GUCY1A3	Hs.24258	guanylate cyclase 1, soluble, alpha 3	3.8538425	0.9731488	-0.9731488
214549_x_at	6698	SPRR1A	Hs.46320	small proline-rich protein 1A	3.853025	0.97299576	-0.97299576

229842_at	1999	ELF3	Hs.67928	E74-like factor 3 (ets domain transcriptior	3.827524	0.96820545	-0.9682059
202644_s_at	7128	TNFAIP3	Hs.211600	tumor necrosis factor, alpha-induced prote	3.819753	0.96673965	-0.96673965
219517_at	619189 /// 80237	ELL3 /// SERIN(	Hs.604985	elongation factor RNA polymerase II-like	3.819022	0.9666014	-0.96660185
33323_r_at	2810	SFN	Hs.523718	stratifin	3.817466	0.96630764	-0.96630764
205251_at	8864	PER2	Hs.58756	period homolog 2 (Drosophila)	3.804326	0.96382046	-0.96382046
233555_s_at	55959	SULF2	Hs.162016	sulfatase 2	3.7991717	0.96284246	-0.96284246
238498_at			Hs.349207		3.7677207	0.95684576	-0.95684624
221655_x_at	54869	EPS8L1	Hs.438862	EPS8-like 1	3.7648463	0.9562955	-0.9562955
204751_x_at	1824	DSC2	Hs.95612	desmocollin 2	3.7632017	0.9559803	-0.9559803
225301_s_at	4645	MYO5B	Hs.720076	myosin VB	3.7599294	0.9553528	-0.9553528
205977_s_at	2041	EPHA1	Hs.89839	EPH receptor A1	3.7594435	0.9552598	-0.9552593
219209_at	64135	IFIH1	Hs.163173	interferon induced with helicase C domair	3.7459733	0.9526701	-0.9526706
239835_at	84541	KBTBD8	Hs.116665	kelch repeat and BTB (POZ) domain cont	3.7456763	0.95261335	-0.9526129
219932_at	28965	SLC27A6	Hs.49765	solute carrier family 27 (fatty acid transpo	3.7451787	0.95251703	-0.9525175
1564786_at	338667	LOC338667		hypothetical protein LOC338667	3.7428684	0.95207214	-0.95207214
39402_at	3553	IL1B	Hs.126256	interleukin 1, beta	3.7208822	0.9478226	-0.9478221
202207_at	10123	ARL4C	Hs.723194	ADP-ribosylation factor-like 4C	3.7012484	0.94400597	-0.94400597
237690_at	221393	GPR115	Hs.150131	G protein-coupled receptor 115	3.7010844	0.943974	-0.943974
233045_at	286126	LOC286126	Hs.214040	hypothetical protein LOC286126	3.680611	0.93997264	-0.93997264
206032_at	1825	DSC3	Hs.41690	desmocollin 3	3.6770375	0.9392719	-0.9392719
201976_s_at	4651	MYO10	Hs.481720	myosin X	3.6747532	0.9388237	-0.9388237
242509_at					3.6641376	0.9367366	-0.93673706
1569607_s_at	100132733 /// 28	ANKRD20A1 //,	Hs.632663	ankyrin repeat domain 20 family, member	3.656725	0.93527603	-0.93527603
223278_at	2706	GJB2	Hs.524894	gap junction protein, beta 2, 26kDa	3.6442237	0.932806	-0.93280554
229228_at	9586	CREB5	Hs.437075	cAMP responsive element binding proteir	3.6391072	0.93179226	-0.93179226
235638_at	166824	RASSF6	Hs.590920	Ras association (RalGDS/AF-6) domain f	3.6359608	0.93116856	-0.9311681
1553132_a_at	123036	TC2N	Hs.510262	tandem C2 domains, nuclear	3.6343389	0.9308462	-0.9308467
231460_at	100507511	LOC100507511		hypothetical LOC100507511	3.6306636	0.93011665	-0.93011665
1555310_a_at	56924	PAK6		p21 protein (Cdc42/Rac)-activated kinase	3.6281686	0.92962074	-0.92962074
224435_at	84293	C10orf58	Hs.500333	chromosome 10 open reading frame 58	3.6088607	0.9257717	-0.9257717
231785_at	4909	NTF4	Hs.266902	neurotrophin 4	3.6074605	0.9254918	-0.9254918
238622_at	5912	RAP2B	Hs.98643	RAP2B, member of RAS oncogene family	3.606433	0.9252863	-0.9252863
244780_at	130367	SGPP2	Hs.591604	sphingosine-1-phosphate phosphatase 2	3.6050708	0.925014	-0.92501354
236489_at	266977	GPR110	Hs.256897	G protein-coupled receptor 110	3.5929344	0.9225812	-0.92258143
217523_at	960	CD44	Hs.502328	CD44 molecule (Indian blood group)	3.5876012	0.92150974	-0.92150974
217497_at	1890	TYMP	Hs.592212	thymidine phosphorylase	3.5872536	0.9214401	-0.92143965
210335_at	9182	RASSF9	Hs.527881	Ras association (RalGDS/AF-6) domain f	3.5856428	0.9211159	-0.9211159
239278_at			Hs.444721		3.583368	0.9206581	-0.9206581
224013_s_at	83595	SOX7	Hs.709543	SRY (sex determining region Y)-box 7	3.5757287	0.9191189	-0.9191184
208228_s_at	2263	FGFR2	Hs.533683	fibroblast growth factor receptor 2	3.573745	0.91871834	-0.91871834
242426_at	145957	NRG4	Hs.696574	neuregulin 4	3.5610762	0.91615677	-0.91615653
209324_s_at	6004	RGS16	Hs.413297	regulator of G-protein signaling 16	3.5459528	0.9130864	-0.9130869
211924_s_at	5329	PLAUR	Hs.466871	plasminogen activator, urokinase receptor	3.5419574	0.9122734	-0.9122734

226403_at	147798	TMC4	Hs.355126	transmembrane channel-like 4	3.541016	0.9120817	-0.9120817
232678_at			Hs.677363		3.5392985	0.9117317	-0.9117317
222572_at	54704	PDP1	Hs.22265	pyruvate dehyrogenase phosphatase cataly	3.5383067	0.91152954	-0.91152954
214370_at	6279	S100A8	Hs.416073	S100 calcium binding protein A8	3.5264785	0.90911436	-0.9091139
212364_at	4430	MYO1B	Hs.439620	myosin IB	3.522256	0.90824986	-0.90824986
231776_at	8320	EOMES	Hs.591663	eomesodermin	3.5148258	0.90672636	-0.90672684
236718_at	4651	MYO10	Hs.481720	myosin X	3.5115607	0.9060559	-0.9060564
244536_at			Hs.624309		3.5097864	0.9056916	-0.9056916
201743_at	929	CD14	Hs.163867	CD14 molecule	3.5067692	0.90507126	-0.90507126
234608_at	3909	LAMA3	Hs.436367	laminin, alpha 3	3.503917	0.9044843	-0.9044843
219518_s_at	619189 /// 80237	ELL3 /// SERIN	Hs.604985	elongation factor RNA polymerase II-like	3.4994345	0.90356064	-0.9035611
224027_at	56477	CCL28	Hs.656904	chemokine (C-C motif) ligand 28	3.4930592	0.9022455	-0.9022455
1554079_at	374378	GALNTL4	Hs.655152	UDP-N-acetyl-alpha-D-galactosamine:po	3.4918633	0.9019985	-0.9019985
218717_s_at	55214	LEPREL1	Hs.374191	leprecan-like 1	3.4735699	0.8982096	-0.8982096
218032_at	8303	SNN	Hs.618526	stannin	3.473463	0.89818764	-0.89818716
240284_x_at	100507644	LOC100507644		hypothetical LOC100507644	3.464734	0.8963723	-0.8963723
229552_at	283454	LOC283454	Hs.26605	hypothetical protein LOC283454	3.4541907	0.8941736	-0.8941741
202889_x_at	9053	MAP7	Hs.486548	microtubule-associated protein 7	3.4538927	0.89411163	-0.89411163
202897_at	140885	SIRPA	Hs.724417	signal-regulatory protein alpha	3.4501116	0.8933215	-0.8933215
228698_at	83595	SOX7	Hs.709543	SRY (sex determining region Y)-box 7	3.4268117	0.88843346	-0.88843346
224325_at	8325	FZD8	Hs.302634	frizzled homolog 8 (Drosophila)	3.4212234	0.88725615	-0.88725615
225792_at	51361	HOOK1	Hs.378836	hook homolog 1 (Drosophila)	3.4192903	0.88684845	-0.88684845
205531_s_at	27165	GLS2	Hs.212606	glutaminase 2 (liver, mitochondrial)	3.4192598	0.8868418	-0.88684225
33322_i_at	2810	SFN	Hs.523718	stratifin	3.415723	0.886096	-0.88609505
227266_s_at	2533	FYB	Hs.370503	FYN binding protein	3.4047549	0.88377523	-0.8837757
205931_s_at	9586	CREB5	Hs.437075	cAMP responsive element binding protein	3.4007387	0.8829241	-0.8829241
47560_at	22859	LPHN1	Hs.94229	latrophilin 1	3.3874128	0.88009167	-0.88009214
240180_at			Hs.547712		3.3650823	0.8753209	-0.8753209
206033_s_at	1825	DSC3	Hs.41690	desmocollin 3	3.3573568	0.87366295	-0.87366295
201565_s_at	3398	ID2	Hs.180919	inhibitor of DNA binding 2, dominant neg	3.3559468	0.8733597	-0.87336016
206277_at	5029	P2RY2	Hs.339	purinergic receptor P2Y, G-protein couple	3.3555996	0.8732853	-0.8732853
206517_at	1014	CDH16	Hs.513660	cadherin 16, KSP-cadherin	3.354616	0.8730736	-0.87307405
218779_x_at	54869	EPS8L1	Hs.438862	EPS8-like 1	3.3510077	0.87229776	-0.8722973
208539_x_at	6701	SPRR2B	Hs.568239	small proline-rich protein 2B	3.3391325	0.8697367	-0.8697367
227410_at	131583	FAM43A	Hs.435080	family with sequence similarity 43, memb	3.3290296	0.86755085	-0.86755085
219461_at	56924	PAK6		p21 protein (Cdc42/Rac)-activated kinase	3.3224585	0.8661256	-0.8661256
206122_at	6665	SOX15	Hs.95582	SRY (sex determining region Y)-box 15	3.318207	0.86520195	-0.86520195
233488_at	84659	RNASE7	Hs.525206	ribonuclease, RNase A family, 7	3.3161755	0.8647604	-0.8647599
202643_s_at	7128	TNFAIP3	Hs.211600	tumor necrosis factor, alpha-induced prote	3.3054218	0.8624172	-0.8624172
219976_at	51361	HOOK1	Hs.378836	hook homolog 1 (Drosophila)	3.2977614	0.8607435	-0.8607435
1552928_s_at	257397	TAB3	Hs.188256	TGF-beta activated kinase 1/MAP3K7 bi	3.2875707	0.858511	-0.858511
203397_s_at	2591	GALNT3	Hs.170986	UDP-N-acetyl-alpha-D-galactosamine:po	3.2863019	0.8582325	-0.8582325
218273_s_at	54704	PDP1	Hs.22265	pyruvate dehyrogenase phosphatase cataly	3.283657	0.8576517	-0.8576517

206156_at	2709	GJB5	Hs.198249	gap junction protein, beta 5, 31.1kDa	3.2805057	0.85695934	-0.85695887
244350_at	4651	MYO10	Hs.481720	myosin X	3.2787766	0.8565788	-0.8565788
202800_at	6507	SLC1A3	Hs.481918	solute carrier family 1 (glial high affinity	3.2720127	0.8550892	-0.8550892
201015_s_at	3728	JUP	Hs.514174	junction plakoglobin	3.2602632	0.85249424	-0.85249424
201417_at	6659	SOX4	Hs.643910	SRY (sex determining region Y)-box 4	3.259522	0.8523302	-0.8523302
227897_at			Hs.700713		3.2554264	0.85142326	-0.85142326
1554246_at	149466	C1orf210	Hs.158963	chromosome 1 open reading frame 210	3.2471051	0.84957695	-0.8495772
219528_s_at	64919	BCL11B	Hs.709690	B-cell CLL/lymphoma 11B (zinc finger p	3.2469373	0.84953976	-0.84953976
204794_at	1844	DUSP2	Hs.1183	dual specificity phosphatase 2	3.2456348	0.8492508	-0.84924984
219991_at	56606	SLC2A9	Hs.656895	solute carrier family 2 (facilitated glucose	3.2443895	0.8489733	-0.84897375
227393_at	338440	ANO9	Hs.501622	anoctamin 9	3.226708	0.84503174	-0.84503126
210059_s_at	5603	MAPK13	Hs.178695	mitogen-activated protein kinase 13	3.221811	0.84393597	-0.84393597
226817_at	1824	DSC2	Hs.95612	desmocollin 2	3.2165124	0.84274864	-0.84274864
213923_at	5912	RAP2B	Hs.98643	RAP2B, member of RAS oncogene family	3.2163508	0.8427124	-0.8427124
203713_s_at	3993	LLGL2	Hs.514477	lethal giant larvae homolog 2 (Drosophila	3.2134871	0.8420701	-0.8420696
225611_at	375449	MAST4	Hs.595458	microtubule associated serine/threonine k	3.2107842	0.8414631	-0.8414626
213492_at	1280	COL2A1	Hs.408182	collagen, type II, alpha 1	3.2069893	0.84061	-0.84060955
241455_at	647024	C6orf132	Hs.575337	chromosome 6 open reading frame 132	3.1937366	0.83762264	-0.83762264
210409_at	653483	C6orf124	Hs.520556	chromosome 6 open reading frame 124	3.1853998	0.8357372	-0.8357372
206385_s_at	288	ANK3	Hs.499725	ankyrin 3, node of Ranvier (ankyrin G)	3.1847093	0.8355808	-0.8355808
227717_at	389337	ARHGEF37	Hs.256206	Rho guanine nucleotide exchange factor (	3.1845207	0.8355379	-0.8355384
203074_at	244 /// 653145 ///	ANXA8 /// ANX	Hs.723897	annexin A8 /// annexin A8-like 1 /// annex	3.1693614	0.8320961	-0.8320961
226302_at	5205	ATP8B1	Hs.216623	ATPase, aminophospholipid transporter, c	3.168183	0.83182764	-0.8318281
226870_at	118881	COMTD1	Hs.355333	catechol-O-methyltransferase domain con	3.1628566	0.8306141	-0.8306141
223218_s_at	64332	NFKBIZ	Hs.319171	nuclear factor of kappa light polypeptide g	3.1531496	0.8283968	-0.8283968
220638_s_at	23624	CBLC	Hs.466907	Cas-Br-M (murine) ecotropic retroviral tra	3.1468456	0.82695293	-0.8269534
206864_s_at	8739	HRK	Hs.87247	harakiri, BCL2 interacting protein (contai	3.1443772	0.8263869	-0.8263874
218309_at	55450	CAMK2N1	Hs.197922	calcium/calmodulin-dependent protein kir	3.1381946	0.8249674	-0.8249674
238575_at	114880	OSBPL6	Hs.318775	oxysterol binding protein-like 6	3.130069	0.8230972	-0.8230972
219513_s_at	10045	SH2D3A	Hs.439645	SH2 domain containing 3A	3.1236107	0.8216076	-0.8216071
1552496_a_at	23242	COBL	Hs.99141	cordon-bleu homolog (mouse)	3.1206748	0.82092905	-0.82092905
213764_s_at	8076	MFAP5	Hs.512842	microfibrillar associated protein 5	3.117814	0.82026744	-0.82026744
217014_s_at	563	AZGP1	Hs.546239	alpha-2-glycoprotein 1, zinc-binding	3.1060514	0.81754065	-0.8175411
229221_at	960	CD44	Hs.502328	CD44 molecule (Indian blood group)	3.1037292	0.81700134	-0.81700134
209955_s_at	2191	FAP	Hs.654370	fibroblast activation protein, alpha	3.0960817	0.8152218	-0.8152218
207540_s_at	6850	SYK	Hs.371720	spleen tyrosine kinase	3.0918522	0.8142357	-0.8142357
235199_at	54941	RNF125	Hs.633703	ring finger protein 125	3.0889482	0.8135576	-0.8135581
206132_at	4163	MCC	Hs.593171	mutated in colorectal cancers	3.0825882	0.81207085	-0.8120713
237252_at	7056	THBD	Hs.2030	thrombomodulin	3.0800707	0.81148195	-0.8114815
201130_s_at	999	CDH1	Hs.461086	cadherin 1, type 1, E-cadherin (epithelial)	3.0746758	0.8102174	-0.8102169
209774_x_at	2920	CXCL2	Hs.75765	chemokine (C-X-C motif) ligand 2	3.0691595	0.8089218	-0.8089218
1559034_at	284759	SIRPB2	Hs.721685	signal-regulatory protein beta 2	3.0611582	0.8070388	-0.8070388
209683_at	81553	FAM49A	Hs.467769	family with sequence similarity 49, memb	3.0568414	0.80602074	-0.806021

204798_at	4602	MYB	Hs.606320	v-myb myeloblastosis viral oncogene hom	3.0532663	0.80517673	-0.80517673
232291_at	407975	MIR17HG	Hs.24115	MIR17 host gene (non-protein coding)	3.0400538	0.8020487	-0.8020482
223423_at	26996	GPR160	Hs.231320	G protein-coupled receptor 160	3.033266	0.800436	-0.800436
204675_at	6715	SRD5A1	Hs.552	steroid-5-alpha-reductase, alpha polypepti	3.0244083	0.7983265	-0.7983265
205515_at	8492	PRSS12	Hs.654823	protease, serine, 12 (neurotrypsin, motops	3.0237846	0.7981777	-0.7981777
1569203_at	2920	CXCL2	Hs.75765	chemokine (C-X-C motif) ligand 2	3.0209577	0.797503	-0.797503
238567_at	130367	SGPP2	Hs.591604	sphingosine-1-phosphate phosphatase 2	3.0197167	0.7972069	-0.7972064
232802_at	90019	SYT8	Hs.161031	synaptotagmin VIII	3.0157201	0.7962513	-0.7962513
225344_at	135112	NCOA7	Hs.171426	nuclear receptor coactivator 7	3.0144627	0.79594994	-0.7959509
242354_at			Hs.569458		3.0116403	0.79527473	-0.79527473
212338_at	4642	MYO1D	Hs.602063	myosin ID	3.0036578	0.79336023	-0.79336023
205676_at	1594	CYP27B1	Hs.524528	cytochrome P450, family 27, subfamily B	2.999744	0.79241943	-0.7924199
219412_at	23682	RAB38	Hs.591975	RAB38, member RAS oncogene family	2.9937682	0.7909813	-0.7909813
225987_at	79689	STEAP4	Hs.521008	STEAP family member 4	2.9892654	0.78989553	-0.78989553
225822_at	128218	TMEM125	Hs.104476	transmembrane protein 125	2.9870286	0.7893553	-0.78935575
221942_s_at	2982	GUCY1A3	Hs.24258	guanylate cyclase 1, soluble, alpha 3	2.9866703	0.789269	-0.789269
218559_s_at	9935	MAFB	Hs.169487	v-maf musculoaponeurotic fibrosarcoma o	2.9786732	0.7873354	-0.78733444
221646_s_at	79844	ZDHHC11	Hs.723221	zinc finger, DHHC-type containing 11	2.9702713	0.7852974	-0.7852974
204430_s_at	6518	SLC2A5	Hs.530003	solute carrier family 2 (facilitated glucose	2.9651022	0.7840409	-0.7840409
229598_at	22837	COBLL1	Hs.470457	COBL-like 1	2.9648225	0.78397274	-0.783973
211795_s_at	2533	FYB	Hs.370503	FYN binding protein	2.9642057	0.78382254	-0.783823
201287_s_at	6382	SDC1	Hs.224607	syndecan 1	2.9628196	0.7834854	-0.7834854
225299_at	4645	MYO5B	Hs.720076	myosin VB	2.9587085	0.7824836	-0.78248405
204130_at	3291	HSD11B2	Hs.1376	hydroxysteroid (11-beta) dehydrogenase 2	2.9572158	0.78211975	-0.78211975
202357_s_at	629	CFB	Hs.69771	complement factor B	2.9564495	0.78193283	-0.78193283
223075_s_at	83543	AIF1L	Hs.4944	allograft inflammatory factor 1-like	2.9563909	0.7819185	-0.7819185
213279_at	115817	DHRS1	Hs.723166	dehydrogenase/reductase (SDR family) m	2.9551053	0.78160477	-0.78160477
219630_at	10158	PDZK1IP1	Hs.431099	PDZK1 interacting protein 1	2.9541366	0.78136826	-0.78136826
214071_at	2774	GNAL	Hs.136295	guanine nucleotide binding protein (G pro	2.9508216	0.7805581	-0.7805586
235549_at	255488	RNF144B	Hs.148741	ring finger protein 144B	2.9481494	0.77990484	-0.77990484
210959_s_at	6715	SRD5A1	Hs.552	steroid-5-alpha-reductase, alpha polypepti	2.9449697	0.77912617	-0.77912664
205490_x_at	2707	GJB3	Hs.620718	gap junction protein, beta 3, 31kDa	2.941739	0.7783346	-0.7783346
210749_x_at	780	DDR1	Hs.631988	discoidin domain receptor tyrosine kinase	2.9407706	0.77809715	-0.77809715
238028_at	647024	C6orf132	Hs.575337	chromosome 6 open reading frame 132	2.9374883	0.7772918	-0.7772913
235046_at	8821	INPP4B	Hs.658245	inositol polyphosphate-4-phosphatase, typ	2.9365048	0.77705	-0.77705
207178_s_at	2444	FRK	Hs.89426	fyn-related kinase	2.9352784	0.77674866	-0.77674866
229720_at	573	BAG1	Hs.377484	BCL2-associated athanogene	2.9337537	0.77637386	-0.77637386
244623_at	56479	KCNQ5	Hs.445324	potassium voltage-gated channel, KQT-lil	2.9333494	0.7762747	-0.7762742
228155_at	84293	C10orf58	Hs.500333	chromosome 10 open reading frame 58	2.9329684	0.77618027	-0.7761812
236172_at	1241	LTB4R	Hs.567248	leukotriene B4 receptor	2.9318383	0.77590275	-0.77590275
220262_s_at	65989	DLK2	Hs.337251	delta-like 2 homolog (Drosophila)	2.9283712	0.7750492	-0.7750492
205376_at	8821	INPP4B	Hs.658245	inositol polyphosphate-4-phosphatase, typ	2.9279075	0.77493477	-0.77493525
1559190_s_at	112724	RDH13	Hs.327631	retinol dehydrogenase 13 (all-trans/9-cis)	2.9243746	0.77406406	-0.77406406

228256_s_at	64097	EPB41L4A	Hs.584954	erythrocyte membrane protein band 4.1 lil	2.922987	0.7737217	-0.7737217
237063_at			Hs.180902		2.9158716	0.7719636	-0.7719636
201465_s_at	3725	JUN	Hs.723911	jun proto-oncogene	2.9124715	0.771122	-0.771122
202688_at	8743	TNFSF10	Hs.478275	tumor necrosis factor (ligand) superfamily	2.9101803	0.77055454	-0.77055407
229276_at	57549	IGSF9	Hs.591472	immunoglobulin superfamily, member 9	2.9036443	0.76893234	-0.76893234
214329_x_at	8743	TNFSF10	Hs.478275	tumor necrosis factor (ligand) superfamily	2.9026866	0.7686944	-0.7686944
1554887_at					2.9007483	0.7682123	-0.7682128
209442_x_at	288	ANK3	Hs.499725	ankyrin 3, node of Ranvier (ankyrin G)	2.896623	0.76718616	-0.7671857
224215_s_at	28514	DLL1	Hs.379912	delta-like 1 (Drosophila)	2.8957882	0.766978	-0.766978
218704_at	54894	RNF43	Hs.584916	ring finger protein 43	2.8915493	0.7659216	-0.7659211
238742_x_at			Hs.569458		2.8908346	0.76574326	-0.7657428
205185_at	11005	SPINK5	Hs.331555	serine peptidase inhibitor, Kazal type 5	2.8889558	0.76527405	-0.76527405
215471_s_at	9053	MAP7	Hs.486548	microtubule-associated protein 7	2.8885977	0.7651844	-0.7651849
200606_at	1832	DSP	Hs.519873	desmoplakin	2.8844123	0.7641382	-0.7641392
232701_at			Hs.663547		2.8781707	0.7625761	-0.7625761
228302_x_at	55450	CAMK2N1	Hs.197922	calcium/calmodulin-dependent protein kir	2.8772728	0.76235104	-0.76235104
205805_s_at	4919	ROR1	Hs.654491	receptor tyrosine kinase-like orphan recep	2.8757803	0.7619767	-0.7619767
213107_at	23043	TNIK	Hs.34024	TRAF2 and NCK interacting kinase	2.8679483	0.7600093	-0.76000977
200952_s_at	894	CCND2	Hs.376071	cyclin D2	2.8674157	0.7598758	-0.7598753
215243_s_at	2707	GJB3	Hs.620718	gap junction protein, beta 3, 31kDa	2.8672972	0.75984573	-0.75984573
207169_x_at	780	DDR1	Hs.631988	discoidin domain receptor tyrosine kinase	2.8592494	0.7578182	-0.7578182
1557389_at			Hs.655149		2.856698	0.757174	-0.7571745
203887_s_at	7056	THBD	Hs.2030	thrombomodulin	2.85403	0.75650024	-0.75650024
225955_at	284207	METRNL	Hs.378821	meteorin, glial cell differentiation regulato	2.8507266	0.7556648	-0.7556648
221211_s_at	56911	C21orf7	Hs.222802	chromosome 21 open reading frame 7	2.8490489	0.75524044	-0.75523996
240304_s_at	79838	TMC5	Hs.115838	transmembrane channel-like 5	2.8455076	0.75434303	-0.75434303
226187_at	1040	CDS1	Hs.654899	CDP-diacylglycerol synthase (phosphatida	2.8400543	0.75295925	-0.75295925
90265_at	11033	ADAP1	Hs.644629	ArfGAP with dual PH domains 1	2.8189087	0.74756813	-0.7475686
201466_s_at	3725	JUN	Hs.723911	jun proto-oncogene	2.8148038	0.7465172	-0.7465172
213668_s_at	6659	SOX4	Hs.643910	SRY (sex determining region Y)-box 4	2.8106055	0.7454405	-0.7454405
208083_s_at	3694	ITGB6	Hs.470399	integrin, beta 6	2.8095114	0.7451596	-0.7451596
222217_s_at	11000	SLC27A3	Hs.438723	solute carrier family 27 (fatty acid transpo	2.809206	0.74508095	-0.7450814
230482_at	81849	ST6GALNAC5	Hs.303609	ST6 (alpha-N-acetyl-neuraminyl-2,3-beta-	2.8089988	0.745028	-0.745028
201464_x_at	3725	JUN	Hs.723911	jun proto-oncogene	2.8084993	0.74489975	-0.74489975
222670_s_at	9935	MAFB	Hs.169487	v-maf musculoaponeurotic fibrosarcoma o	2.8083434	0.7448597	-0.7448597
205249_at	1959	EGR2	Hs.1395	early growth response 2	2.8080947	0.7447958	-0.7447958
218902_at	4851	NOTCH1	Hs.495473	notch 1	2.7995727	0.7426033	-0.7426033
232609_at	92359	CRB3	Hs.150319	crumbs homolog 3 (Drosophila)	2.7960966	0.74170685	-0.7417073
205774_at	2161	F12	Hs.1321	coagulation factor XII (Hageman factor)	2.7947466	0.74135876	-0.74135876
228260_at	1993	ELAVL2	Hs.166109	ELAV (embryonic lethal, abnormal vision	2.7926848	0.7408266	-0.74082613
235144_at	158158	RASEF	Hs.657750	RAS and EF-hand domain containing	2.7919943	0.74064827	-0.7406478
223454_at	58191	CXCL16	Hs.724659	chemokine (C-X-C motif) ligand 16	2.7874072	0.7394619	-0.7394619
1553639_a_at	133522	PPARGC1B	Hs.483816	peroxisome proliferator-activated receptor	2.7872396	0.7394185	-0.7394185

204519_s_at	51090	PLLP	Hs.632215	plasmolipin	2.7869983	0.73935604	-0.73935604
208779_x_at	780	DDR1	Hs.631988	discoidin domain receptor tyrosine kinase	2.7857568	0.73903465	-0.73903465
201329_s_at	2114	ETS2	Hs.644231	v-ets erythroblastosis virus E26 oncogene	2.779804	0.7374916	-0.7374916
239350_at	91862	MARVELD3	Hs.513706	MARVEL domain containing 3	2.7755222	0.7363796	-0.7363796
223895_s_at	55040	EPN3	Hs.670090	epsin 3	2.770855	0.7351656	-0.7351656
222892_s_at	55287	TMEM40	Hs.475502	transmembrane protein 40	2.7683825	0.73452187	-0.7345214
204224_s_at	2643	GCH1	Hs.86724	GTP cyclohydrolase 1	2.7669892	0.7341585	-0.7341585
226489_at	57458	TMCC3	Hs.370410	transmembrane and coiled-coil domain fa	2.7633188	0.733201	-0.733201
233317_at	928	CD9	Hs.114286	CD9 molecule	2.7628512	0.73307896	-0.73307896
201939_at	10769	PLK2	Hs.398157	polo-like kinase 2	2.7613487	0.732687	-0.73268604
203854_at	3426	CFI	Hs.312485	complement factor I	2.758108	0.7318392	-0.73183966
220588_at	55653	BCAS4	Hs.381178	breast carcinoma amplified sequence 4	2.7555192	0.7311621	-0.7311621
1563900_at	222584	FAM83B	Hs.657974	family with sequence similarity 83, memb	2.7515476	0.7301216	-0.7301216
206698_at	7504	XK	Hs.78919	X-linked Kx blood group (McLeod syndro	2.7390857	0.7268472	-0.7268472
226666_at	23002	DAAM1	Hs.654934	dishevelled associated activator of morph	2.7367744	0.72623825	-0.72623825
219352_at	55008	HERC6	Hs.529317	hect domain and RLD 6	2.7267847	0.7236004	-0.7236004
206400_at	3963 /// 653499	LGALS7 /// LGA	Hs.707031	lectin, galactoside-binding, soluble, 7 /// l	2.714994	0.7204747	-0.72047424
227405_s_at	8325	FZD8	Hs.302634	frizzled homolog 8 (Drosophila)	2.7140932	0.7202349	-0.72023535
1560531_at	353132	LCE1B	Hs.375103	late cornified envelope 1B	2.7138464	0.72016954	-0.72016954
222354_at	50848	F11R	Hs.517293	F11 receptor	2.7128375	0.7199011	-0.71990156
203828_s_at	9235	IL32	Hs.943	interleukin 32	2.7115395	0.71955633	-0.71955585
242874_at			Hs.606505		2.7107177	0.71933746	-0.71933746
232290_at			Hs.655775		2.7063777	0.7181816	-0.7181816
208165_s_at	10279	PRSS16	Hs.274407	protease, serine, 16 (thymus)	2.7010205	0.71675205	-0.7167525
215074_at	4430	MYO1B	Hs.439620	myosin IB	2.700941	0.7167311	-0.7167311
219454_at	25975	EGFL6	Hs.12844	EGF-like-domain, multiple 6	2.699877	0.7164469	-0.7164469
225283_at	91947	ARRDC4	Hs.6093	arrestin domain containing 4	2.6949308	0.71512413	-0.71512413
206865_at	8739	HRK	Hs.87247	harakiri, BCL2 interacting protein (contai	2.6936076	0.71476984	-0.71476984
207126_x_at	54575 /// 54576 /	UGT1A1 /// UGT1A10 /// UGT1A4 //		UDP glucuronosyltransferase 1 family, po	2.6877186	0.71319103	-0.71319103
232151_at	346389	MACC1	Hs.598388	metastasis associated in colon cancer 1	2.6866171	0.7128954	-0.7128954
218815_s_at	55092	TMEM51	Hs.465305	transmembrane protein 51	2.6864786	0.7128582	-0.7128582
223217_s_at	64332	NFKBIZ	Hs.319171	nuclear factor of kappa light polypeptide g	2.684604	0.71235466	-0.71235466
231926_at	58513	EPS15L1	Hs.654639	epidermal growth factor receptor pathway	2.6809108	0.7113619	-0.7113614
230430_at	954	ENTPD2	Hs.123036	ectonucleoside triphosphate diphosphohyd	2.680777	0.71132565	-0.71132565
227021_at	221656	KDM1B	Hs.709336	lysine (K)-specific demethylase 1B	2.6786206	0.71074533	-0.71074486
203888_at	7056	THBD	Hs.2030	thrombomodulin	2.6731927	0.7092819	-0.7092819
1552797_s_at	150696	PROM2	Hs.469313	prominin 2	2.6730902	0.70925426	-0.70925426
217744_s_at	64065	PERP	Hs.201446	PERP, TP53 apoptosis effector	2.6724718	0.7090874	-0.7090874
210058_at	5603	MAPK13	Hs.178695	mitogen-activated protein kinase 13	2.672138	0.70899725	-0.70899725
40016_g_at	375449	MAST4	Hs.595458	microtubule associated serine/threonine k	2.6713626	0.7087879	-0.7087879
204714_s_at	2153	F5	Hs.30054	coagulation factor V (proaccelerin, labile	2.6690838	0.7081723	-0.7081723
202687_s_at	8743	TNFSF10	Hs.478275	tumor necrosis factor (ligand) superfamily	2.6609933	0.7059827	-0.7059822
209126_x_at	3854	KRT6B	Hs.708950	keratin 6B	2.6564844	0.70475864	-0.7047596

241021_at			Hs.667718		2.6529818	0.70380735	-0.70380735
226068_at	6850	SYK	Hs.371720	spleen tyrosine kinase	2.652167	0.7035861	-0.7035856
200924_s_at	6520	SLC3A2	Hs.502769	solute carrier family 3 (activators of dibas	2.6518893	0.7035103	-0.7035103
218792_s_at	54836	BSPRY	Hs.614517	B-box and SPRY domain containing	2.648812	0.70267296	-0.7026725
218990_s_at	6707	SPRR3	Hs.139322	small proline-rich protein 3	2.6478002	0.70239735	-0.70239687
213085_s_at	23286	WWC1	Hs.484047	WW and C2 domain containing 1	2.6477275	0.7023773	-0.7023773
223805_at	114880	OSBPL6	Hs.318775	oxysterol binding protein-like 6	2.6475945	0.7023411	-0.7023411
242053_at			Hs.596071		2.6455922	0.7017951	-0.7017956
242873_at			Hs.638425		2.6441393	0.7013993	-0.70139885
227461_at	85439	STON2	Hs.14248	stonin 2	2.6430376	0.70109844	-0.70109844
209569_x_at	27065	D4S234E	Hs.518595	DNA segment on chromosome 4 (unique)	2.6407218	0.70046616	-0.70046616
206115_at	1960	EGR3	Hs.534313	early growth response 3	2.6385336	0.6998682	-0.6998682
230708_at	144165	PRICKLE1	Hs.720221	prickle homolog 1 (Drosophila)	2.6382258	0.6997838	-0.6997843
205109_s_at	50649	ARHGEF4	Hs.469935	Rho guanine nucleotide exchange factor (	2.6378458	0.6996803	-0.69967985
208123_at	9312	KCNB2	Hs.661102	potassium voltage-gated channel, Shab-re	2.6376147	0.6996169	-0.6996169
202575_at	1382	CRABP2	Hs.405662	cellular retinoic acid binding protein 2	2.6260297	0.69644165	-0.69644165
209125_at	3853	KRT6A	Hs.700779	keratin 6A	2.6197119	0.69470406	-0.69470406
231102_at	54677	CROT	Hs.125039	carnitine O-octanoyltransferase	2.6186478	0.6944108	-0.6944113
209720_s_at	6317	SERPINB3	Hs.227948	serpin peptidase inhibitor, clade B (ovalbu	2.6176095	0.69412494	-0.69412494
201328_at	2114	ETS2	Hs.644231	v-ets erythroblastosis virus E26 oncogene	2.6125798	0.6927376	-0.6927376
236261_at	114880	OSBPL6	Hs.318775	oxysterol binding protein-like 6	2.6090531	0.6917629	-0.6917634
204713_s_at	2153	F5	Hs.30054	coagulation factor V (proaccelerin, labile	2.6089764	0.69174194	-0.69174194
227204_at	84552	PARD6G	Hs.654920	par-6 partitioning defective 6 homolog ga	2.607721	0.6913948	-0.6913948
239775_at					2.6054485	0.69076586	-0.69076586
1564796_at	2012	EMP1	Hs.436298	epithelial membrane protein 1	2.6015692	0.68969107	-0.68969107
225496_s_at	54843	SYTL2	Hs.369520	synaptotagmin-like 2	2.599216	0.6890383	-0.6890383
1553589_a_at	10158	PDZK1IP1	Hs.431099	PDZK1 interacting protein 1	2.59854	0.6888504	-0.6888509
230179_at	285812	LOC285812	Hs.593631	hypothetical protein LOC285812	2.5902352	0.68654156	-0.68654156
1554921_a_at	8796	SCEL	Hs.534699	sciellin	2.5859525	0.68534803	-0.6853478
244665_at			Hs.668855		2.5826082	0.6844144	-0.6844144
226912_at	254887	ZDHHC23	Hs.21902	zinc finger, DHHC-type containing 23	2.5806816	0.68387604	-0.68387604
225020_at	153090	DAB2IP	Hs.522378	DAB2 interacting protein	2.578751	0.68333626	-0.68333626
212558_at	10252	SPRY1	Hs.436944	sprouty homolog 1, antagonist of FGF sig	2.5774848	0.68298197	-0.68298197
239196_at	118932	ANKRD22	Hs.217484	ankyrin repeat domain 22	2.577311	0.68293333	-0.68293333
1555421_at	130340	AP1S3	Hs.632555	adaptor-related protein complex 1, sigma	2.5765429	0.6827183	-0.6827183
213267_at	100509911 /// 23	DOPEY1 /// LO(	Hs.520246	dopey family member 1 /// hypothetical L(	2.576542	0.6827178	-0.6827183
212560_at	6653	SORL1	Hs.368592	sortilin-related receptor, L(DLR class) A r	2.5747304	0.6822109	-0.68221045
1553986_at	158158	RASEF	Hs.657750	RAS and EF-hand domain containing	2.5736575	0.68191004	-0.68191004
201656_at	3655	ITGA6	Hs.133397	integrin, alpha 6	2.573547	0.68187904	-0.68187904
214580_x_at	286887 /// 3853 /	KRT6A /// KRT(	Hs.708950	keratin 6A /// keratin 6B /// keratin 6C	2.573506	0.6818676	-0.6818676
202206_at	10123	ARL4C	Hs.723194	ADP-ribosylation factor-like 4C	2.5689187	0.6805806	-0.6805806
223784_at	57393	TMEM27	Hs.129614	transmembrane protein 27	2.5689187	0.6805806	-0.6805806
225467_s_at	112724	RDH13	Hs.327631	retinol dehydrogenase 13 (all-trans/9-cis)	2.5676336	0.68021965	-0.68021965

1007_s_at	780	DDR1	Hs.631988	discoidin domain receptor tyrosine kinase	2.5657501	0.67969036	-0.67969036
204019_s_at	26751	SH3YL1	Hs.515951	SH3 domain containing, Ysc84-like 1 (S.	2.5581276	0.67754364	-0.6775446
228975_at	80320	SP6	Hs.253603	Sp6 transcription factor	2.5580795	0.67753077	-0.6775303
209173_at	10551	AGR2	Hs.530009	anterior gradient homolog 2 (Xenopus lae	2.5575907	0.6773925	-0.67739296
226200_at	57176	VARS2	Hs.597526	valyl-tRNA synthetase 2, mitochondrial (p	2.5520184	0.6758194	-0.6758194
233634_at	91862	MARVELD3	Hs.513706	MARVEL domain containing 3	2.5512679	0.6756072	-0.6756072
235310_at	257144	GCET2	Hs.49614	germinal center expressed transcript 2	2.5466814	0.67430925	-0.67430925
244353_s_at	154091	SLC2A12	Hs.486508	solute carrier family 2 (facilitated glucose	2.5442753	0.6736274	-0.6736274
219648_at	55686	MREG	Hs.707104	melanoregulin	2.5431032	0.673295	-0.673295
236595_at	100507307	LOC100507307		hypothetical LOC100507307	2.5381029	0.6718755	-0.671875
238513_at	79056	PRRG4	Hs.471695	Proline rich Gla (G-carboxyglutamic acid)	2.5380952	0.6718731	-0.6718731
210026_s_at	29775	CARD10	Hs.57973	caspase recruitment domain family, memb	2.5325377	0.6702919	-0.6702919
205309_at	27293	SMPDL3B	Hs.123659	sphingomyelin phosphodiesterase, acid-lil	2.5252516	0.66821384	-0.66821337
231858_x_at	91056	DKFZp761E198	Hs.591957	DKFZp761E198 protein	2.5241094	0.6678872	-0.6678872
223322_at	83593	RASSF5	Hs.497579	Ras association (RalGDS/AF-6) domain f	2.522815	0.6675172	-0.6675172
216641_s_at	3898	LAD1	Hs.519035	ladinin 1	2.517766	0.6660719	-0.66607237
203765_at	25801	GCA	Hs.377894	grancalcin, EF-hand calcium binding prot	2.5166724	0.6657586	-0.66575885
209631_s_at	2861	GPR37	Hs.723816	G protein-coupled receptor 37 (endothelin	2.513239	0.66477394	-0.66477394
203586_s_at	379	ARL4D	Hs.183153	ADP-ribosylation factor-like 4D	2.5118861	0.6643858	-0.6643853
226065_at	144165	PRICKLE1	Hs.524348	prickle homolog 1 (Drosophila)	2.5102396	0.6639123	-0.6639128
231311_at			Hs.634674		2.5094945	0.6636982	-0.6636987
228221_at	126969	SLC44A3	Hs.483423	solute carrier family 44, member 3	2.5078065	0.66321325	-0.6632128
200635_s_at	5792	PTPRF	Hs.272062	protein tyrosine phosphatase, receptor typ	2.503711	0.66203403	-0.66203403
219395_at	80004	ESRP2	Hs.592053	epithelial splicing regulatory protein 2	2.503165	0.6618767	-0.6618767
227806_at	404550	C16orf74	Hs.461655	chromosome 16 open reading frame 74	2.498593	0.66055775	-0.6605582
219115_s_at	53832	IL20RA	Hs.445868	interleukin 20 receptor, alpha	2.4953308	0.6596155	-0.6596155
229952_at			Hs.660389		2.495127	0.6595564	-0.65955687
225566_at	8828	NRP2	Hs.471200	neuropilin 2	2.4936702	0.65913534	-0.65913534
201566_x_at	3398	ID2	Hs.180919	inhibitor of DNA binding 2, dominant neg	2.4906604	0.65826416	-0.65826416
224240_s_at	56477	CCL28	Hs.656904	chemokine (C-C motif) ligand 28	2.4904406	0.65820074	-0.65820026
203332_s_at	3635	INPP5D	Hs.262886	inositol polyphosphate-5-phosphatase, 145	2.4840407	0.6563444	-0.6563444
237187_at			Hs.610726		2.4797177	0.65508795	-0.65508795
233565_s_at	27111	SDCBP2	Hs.657015	syndecan binding protein (syntenin) 2	2.4759784	0.6539993	-0.6539993
228787_s_at	55653	BCAS4	Hs.381178	breast carcinoma amplified sequence 4	2.473706	0.653337	-0.653337
228885_at	256691	MAMDC2	Hs.547172	MAM domain containing 2	2.4730911	0.6531577	-0.6531577
202193_at	3985	LIMK2	Hs.474596	LIM domain kinase 2	2.4661448	0.65112877	-0.65112877
1552487_a_at	646	BNC1	Hs.459153	basonuclin 1	2.463844	0.6504555	-0.6504555
219684_at	64108	RTP4	Hs.43388	receptor (chemosensory) transporter prote	2.4602425	0.64940023	-0.64940023
1555829_at	57488	ESYT2	Hs.490795	extended synaptotagmin-like protein 2	2.4592202	0.6491003	-0.64910054
229225_at	8828	NRP2	Hs.471200	neuropilin 2	2.4585922	0.64891624	-0.64891624
216268_s_at	182	JAG1	Hs.724464	jagged 1	2.4541626	0.64761543	-0.64761543
222642_s_at	55161	TMEM33	Hs.31082	transmembrane protein 33	2.4525132	0.64713	-0.64713097
204667_at	3169	FOXA1	Hs.163484	forkhead box A1	2.4495938	0.6462712	-0.6462712

218844_at	80221	ACSF2	Hs.288959	acyl-CoA synthetase family member 2	2.4445612	0.6447878	-0.6447878
236543_at			Hs.130203		2.4432478	0.6444001	-0.6444001
210145_at	5321	PLA2G4A	Hs.497200	phospholipase A2, group IVA (cytosolic,	2.4413993	0.64385414	-0.64385414
235605_at	54897	CASZ1	Hs.439894	castor zinc finger 1	2.4330337	0.6413779	-0.6413784
216060_s_at	23002	DAAM1	Hs.654934	dishevelled associated activator of morph	2.4278772	0.63984776	-0.63984776
1555772_a_at	993	CDC25A	Hs.437705	cell division cycle 25 homolog A (S. pomb	2.4229417	0.63838005	-0.6383796
232001_at	439949	LOC439949	Hs.590987	hypothetical LOC439949	2.420826	0.6377497	-0.6377497
222870_s_at	10678	B3GNT2	Hs.173203	UDP-GlcNAc:betaGal beta-1,3-N-acetylg	2.419722	0.63742065	-0.63742065
203144_s_at	9674	KIAA0040	Hs.518138	KIAA0040	2.4189544	0.6371918	-0.6371918
218780_at	29911	HOOK2	Hs.30792	hook homolog 2 (Drosophila)	2.414574	0.6358843	-0.6358843
218465_at	55161	TMEM33	Hs.31082	transmembrane protein 33	2.4128156	0.6353588	-0.6353588
1555724_s_at	6876	TAGLN	Hs.410977	transgelin	2.411799	0.6350546	-0.63505507
201005_at	928	CD9	Hs.114286	CD9 molecule	2.407106	0.6336498	-0.6336498
208596_s_at	54575 /// 54576 /	UGT1A1 /// UGT1A10 /// UGT1A3 //		UDP glucuronosyltransferase 1 family, pc	2.40309	0.63244534	-0.63244534
1564706_s_at	27165	GLS2	Hs.212606	glutaminase 2 (liver, mitochondrial)	2.3993528	0.6313224	-0.63132286
214734_at	23086	EXPH5	Hs.28540	exophilin 5	2.393587	0.6295872	-0.6295872
223233_s_at	57530	CGN	Hs.591464	cingulin	2.3923113	0.62920284	-0.62920237
200636_s_at	5792	PTPRF	Hs.272062	protein tyrosine phosphatase, receptor typ	2.3905292	0.628665	-0.628665
207557_s_at	6262	RYR2	Hs.109514	ryanodine receptor 2 (cardiac)	2.389434	0.6283345	-0.6283345
219142_at	65997	RASL11B	Hs.8035	RAS-like, family 11, member B	2.3894105	0.62832737	-0.62832737
223748_at	83959	SLC4A11	Hs.105607	solute carrier family 4, sodium borate tran	2.3879673	0.62789154	-0.62789154
206581_at	646	BNC1	Hs.459153	basonuclin 1	2.3874242	0.6277275	-0.6277275
227195_at	84858	ZNF503	Hs.195710	zinc finger protein 503	2.3869445	0.62758255	-0.62758255
222891_s_at	53335	BCL11A	Hs.370549	B-cell CLL/lymphoma 11A (zinc finger p	2.384828	0.62694263	-0.62694263
232662_x_at	84293	C10orf58	Hs.718589	chromosome 10 open reading frame 58	2.3842826	0.62677765	-0.62677765
240861_at					2.384007	0.6266942	-0.6266942
222348_at					2.3834412	0.626523	-0.626523
231192_at	23566	LPAR3	Hs.674915	lysophosphatidic acid receptor 3	2.3831904	0.6264472	-0.62644696
226560_at			Hs.210043		2.3825622	0.62625694	-0.62625694
229933_at	148304	C1orf74	Hs.497642	chromosome 1 open reading frame 74	2.3822606	0.6261654	-0.62616587
205266_at	3976	LIF	Hs.2250	leukemia inhibitory factor (cholinergic dif	2.3810823	0.6258087	-0.6258087
45633_at	64785	GINS3	Hs.47125	GINS complex subunit 3 (Psf3 homolog)	2.3800342	0.62549114	-0.62549114
234970_at	123036	TC2N	Hs.510262	tandem C2 domains, nuclear	2.379696	0.6253886	-0.6253886
228918_at	100288617	LOC100288617	Hs.720040	Hypothetical protein LOC100288617	2.3790975	0.6252074	-0.62520695
219497_s_at	53335	BCL11A	Hs.370549	B-cell CLL/lymphoma 11A (zinc finger p	2.3787878	0.6251135	-0.625113
206391_at	5918	RARRES1	Hs.131269	retinoic acid receptor responder (tazaroter	2.3753238	0.62406206	-0.62406206
211661_x_at	5724	PTAFR	Hs.433540	platelet-activating factor receptor	2.374662	0.62386084	-0.6238613
235269_at	113828	FAM83F	Hs.197680	family with sequence similarity 83, memb	2.3742726	0.6237426	-0.62374306
1555801_s_at	151126	ZNF385B	Hs.655005	zinc finger protein 385B	2.3713768	0.62286234	-0.6228626
205466_s_at	9957	HS3ST1	Hs.507348	heparan sulfate (glucosamine) 3-O-sulfotr	2.3693552	0.6222472	-0.6222472
213191_at	148022	TICAM1	Hs.29344	toll-like receptor adaptor molecule 1	2.3653762	0.6210346	-0.6210351
220052_s_at	26277	TINF2	Hs.496191	TERF1 (TRF1)-interacting nuclear factor	2.365019	0.6209259	-0.6209259
219429_at	79152	FA2H	Hs.461329	fatty acid 2-hydroxylase	2.3644845	0.6207628	-0.6207628

1559132_at	283232	TMEM80	Hs.448664	transmembrane protein 80	2.3595498	0.619256	-0.61925554
204268_at	6273	S100A2	Hs.516484	S100 calcium binding protein A2	2.3589203	0.6190634	-0.6190634
230769_at	163259	DENND2C	Hs.654928	DENN/MADD domain containing 2C	2.3584495	0.6189194	-0.6189194
231183_s_at	182	JAG1	Hs.724464	Jagged 1 (Alagille syndrome)	2.3576202	0.6186657	-0.6186657
208156_x_at	83481	EPPK1	Hs.200412	epiplakin 1	2.3559813	0.61816406	-0.61816406
201422_at	10437	IFI30	Hs.14623	interferon, gamma-inducible protein 30	2.35271	0.61716175	-0.61716175
231726_at	56122	PCDHB14	Hs.658497	protocadherin beta 14	2.3504732	0.6164756	-0.6164756
202934_at	3099	HK2	Hs.406266	hexokinase 2	2.3489618	0.6160116	-0.6160116
1566232_at			Hs.652960		2.348236	0.61578846	-0.61578894
200637_s_at	5792	PTPRF	Hs.272062	protein tyrosine phosphatase, receptor typ	2.3461103	0.61513567	-0.6151352
240135_x_at			Hs.716603		2.345753	0.6150255	-0.6150255
1556499_s_at	1277	COL1A1	Hs.172928	collagen, type I, alpha 1	2.3394566	0.6130867	-0.6130867
204029_at	1952	CELSR2	Hs.57652	cadherin, EGF LAG seven-pass G-type re	2.338936	0.6129265	-0.612926
1564651_at	221710	LOC221710	Hs.656600	hypothetical protein LOC221710	2.3382328	0.61270905	-0.6127095
213913_s_at	23329	TBC1D30	Hs.192492	TBC1 domain family, member 30	2.3361526	0.6120672	-0.6120672
225645_at	26298	EHF	Hs.653859	ets homologous factor	2.3353403	0.6118164	-0.6118164
36499_at	1952	CELSR2	Hs.57652	cadherin, EGF LAG seven-pass G-type re	2.33426	0.6114826	-0.6114826
222895_s_at	64919	BCL11B	Hs.709690	B-cell CLL/lymphoma 11B (zinc finger p	2.3305438	0.61033344	-0.6103332
209212_s_at	688	KLF5	Hs.508234	Kruppel-like factor 5 (intestinal)	2.32502	0.6086216	-0.6086216
209098_s_at	182	JAG1	Hs.724464	jagged 1	2.3237348	0.6082225	-0.60822296
209782_s_at	1628	DBP	Hs.528006	D site of albumin promoter (albumin D-bo	2.3223467	0.6077914	-0.6077919
221232_s_at	26287	ANKRD2	Hs.73708	ankyrin repeat domain 2 (stretch responsiv	2.3207207	0.60728645	-0.60728645
205547_s_at	6876	TAGLN	Hs.410977	transgelin	2.3201983	0.6071243	-0.60712385
219168_s_at	55615	PRR5	Hs.102336	proline rich 5 (renal)	2.3194048	0.6068773	-0.6068773
223059_s_at	83641	FAM107B	Hs.446315	family with sequence similarity 107, mem	2.3193197	0.6068506	-0.6068511
238673_at			Hs.701196		2.3190162	0.6067567	-0.6067562
227379_at	154141	MBOAT1	Hs.377830	membrane bound O-acyltransferase doma	2.3163443	0.6059246	-0.6059251
227347_x_at	57801	HES4	Hs.154029	hairy and enhancer of split 4 (Drosophila)	2.3147807	0.60543776	-0.60543776
203143_s_at	9674	KIAA0040	Hs.518138	KIAA0040	2.314122	0.60523224	-0.6052327
1552580_at	205860	TRIML2	Hs.276429	tripartite motif family-like 2	2.3131883	0.60494137	-0.60494137
240877_x_at			Hs.664374		2.3116148	0.6044507	-0.6044502
1557522_x_at			Hs.595233		2.3114893	0.6044111	-0.6044116
218501_at	50650	ARHGEF3	Hs.476402	Rho guanine nucleotide exchange factor (	2.3109448	0.6042414	-0.6042414
243856_at	347404	LANCL3	Hs.521932	LanC lantibiotic synthetase component C-	2.3096633	0.6038413	-0.6038413
227498_at	55553	SOX6	Hs.368226	SRY (sex determining region Y)-box 6	2.309425	0.6037669	-0.6037669
220513_at	100129128	KHDC1L	Hs.663639	KH homology domain containing 1-like	2.3069108	0.6029811	-0.6029811
238909_at					2.303852	0.6020241	-0.6020241
218062_x_at	23580	CDC42EP4	Hs.3903	CDC42 effector protein (Rho GTPase bin	2.3036726	0.6019678	-0.6019678
232682_at	55686	MREG	Hs.707104	melanoregulin	2.3025086	0.60160303	-0.6016035
1552304_at	84920	ALG10	Hs.102971	asparagine-linked glycosylation 10, alpha-	2.3022673	0.6015277	-0.6015277
204201_s_at	5783	PTPN13	Hs.436142	protein tyrosine phosphatase, non-recepto	2.3009696	0.60112095	-0.60112095
239012_at	255488	RNF144B	Hs.148741	Ring finger protein 144B	2.3008661	0.6010885	-0.6010885
227072_at	25914	RTTN	Hs.654809	rotatin	2.3006198	0.6010113	-0.6010113

222937_s_at	79148	MMP28	Hs.380710	matrix metallopeptidase 28	2.2997599	0.6007414	-0.60074186
235667_at	643783	LOC643783	Hs.631514	hypothetical LOC643783	2.2996664	0.6007123	-0.6007123
204750_s_at	1824	DSC2	Hs.95612	desmocollin 2	2.298424	0.60032225	-0.6003227
223000_s_at	50848	F11R	Hs.517293	F11 receptor	2.2923615	0.5984173	-0.5984173
226246_at	284252	KCTD1	Hs.526630	potassium channel tetramerisation domair	2.2870913	0.59675694	-0.59675694
207735_at	54941	RNF125	Hs.633703	ring finger protein 125	2.2866302	0.5966115	-0.5966115
239412_at	3663	IRF5	Hs.521181	interferon regulatory factor 5	2.2864585	0.59655714	-0.5965576
219411_at	79767	ELMO3	Hs.377416	engulfment and cell motility 3	2.2848184	0.5960398	-0.5960398
219503_s_at	55287	TMEM40	Hs.475502	transmembrane protein 40	2.278746	0.59412	-0.59412
219091_s_at	79812	MMRN2	Hs.524479	multimerin 2	2.2765958	0.5934391	-0.5934391
242627_at			Hs.682433		2.275854	0.593204	-0.593204
230005_at	258010	SVIP	Hs.349096	small VCP/p97-interacting protein	2.2721186	0.5920191	-0.5920191
228707_at	137075	CLDN23	Hs.183617	claudin 23	2.2673826	0.5905142	-0.5905137
231365_at					2.2661576	0.59012413	-0.59012413
212812_at	256987	SERINC5	Hs.655558	serine incorporator 5	2.2645254	0.5896044	-0.5896044
214763_at	26027	ACOT11	Hs.724769	acyl-CoA thioesterase 11	2.2624853	0.588954	-0.58895445
221701_s_at	64220	STRA6	Hs.24553	stimulated by retinoic acid gene 6 homolo	2.2571821	0.5872617	-0.5872612
201693_s_at	1958	EGR1	Hs.326035	early growth response 1	2.256886	0.5871668	-0.5871668
225459_at	154810	AMOTL1	Hs.503594	angiomotin like 1	2.2553246	0.58666754	-0.58666754
227599_at	151963	C3orf59	Hs.151443	chromosome 3 open reading frame 59	2.2550547	0.58658123	-0.58658123
239660_at	57186	RALGAPA2	Hs.472285	Ral GTPase activating protein, alpha subu	2.2543087	0.58634233	-0.5863428
242450_at	285704	RGMB	Hs.526902	RGM domain family, member B	2.2528946	0.5858898	-0.58589005
1565868_at	960	CD44	Hs.502328	CD44 molecule (Indian blood group)	2.2526338	0.58580637	-0.58580637
227802_at	22902	RUFY3	Hs.724391	RUN and FYVE domain containing 3	2.2497334	0.584877	-0.584877
226245_at	284252	KCTD1	Hs.526630	potassium channel tetramerisation domair	2.2463393	0.5837879	-0.5837879
209283_at	1410	CRYAB	Hs.53454	crystallin, alpha B	2.2460623	0.58369875	-0.5836992
202965_s_at	827	CAPN6	Hs.496593	calpain 6	2.2458522	0.5836315	-0.5836315
213194_at	6091	ROBO1	Hs.13640	roundabout, axon guidance receptor, hom	2.2446442	0.58324337	-0.58324337
225757_s_at	79789	CLMN	Hs.301478	calmin (calponin-like, transmembrane)	2.2427294	0.5826278	-0.5826278
1552257_a_at	23170	TTLL12	Hs.517670	tubulin tyrosine ligase-like family, membe	2.2419808	0.582387	-0.582387
1553413_at	80078	FLJ13744		hypothetical FLJ13744	2.2419217	0.5823679	-0.5823679
215129_at	5288	PIK3C2G	Hs.22500	phosphoinositide-3-kinase, class 2, gamm	2.2409332	0.58204985	-0.58204985
203153_at	3434	IFIT1	Hs.20315	interferon-induced protein with tetratricop	2.2402904	0.5818429	-0.5818429
225667_s_at	151354	FAM84A	Hs.260855	family with sequence similarity 84, memb	2.2401645	0.58180237	-0.58180237
205626_s_at	793	CALB1	Hs.65425	calbindin 1, 28kDa	2.2355847	0.5803261	-0.5803261
201170_s_at	8553	BHLHE40	Hs.171825	basic helix-loop-helix family, member e40	2.2350438	0.58015156	-0.58015156
209211_at	688	KLF5	Hs.508234	Kruppel-like factor 5 (intestinal)	2.2346435	0.58002186	-0.5800228
204653_at	7020	TFAP2A	Hs.519880	transcription factor AP-2 alpha (activating	2.234453	0.5799608	-0.5799608
235775_at	160335	TMTC2	Hs.577775	transmembrane and tetratricopeptide repea	2.2342002	0.5798793	-0.5798793
230205_at	93134	ZNF561	Hs.720081	zinc finger protein 561	2.233611	0.579689	-0.579689
226122_at	57480	PLEKHG1	Hs.189781	pleckstrin homology domain containing, f	2.2332716	0.57957935	-0.57957935
205016_at	7039	TGFA	Hs.170009	transforming growth factor, alpha	2.2319698	0.5791588	-0.5791588
226021_at	157506	RDH10	Hs.724648	retinol dehydrogenase 10 (all-trans)	2.231891	0.57913303	-0.5791335

205777_at	1852	DUSP9	Hs.605100	dual specificity phosphatase 9	2.2315936	0.5790372	-0.5790372
200666_s_at	3337	DNAJB1	Hs.515210	DnaJ (Hsp40) homolog, subfamily B, mer	2.2308075	0.57878304	-0.57878304
204717_s_at	3177	SLC29A2	Hs.569017	solute carrier family 29 (nucleoside transp	2.2306106	0.5787196	-0.57871914
209193_at	5292	PIM1	Hs.81170	pim-1 oncogene	2.229996	0.5785208	-0.5785203
238805_at	91894	C11orf52	Hs.97013	chromosome 11 open reading frame 52	2.2255876	0.5770931	-0.5770931
220520_s_at	54830	NUP62CL	Hs.163629	nucleoporin 62kDa C-terminal like	2.224264	0.576664	-0.576664
206561_s_at	57016	AKR1B10	Hs.116724	aldo-keto reductase family 1, member B1(	2.2228527	0.5762062	-0.5762062
209684_at	54453	RIN2	Hs.472270	Ras and Rab interactor 2	2.2221756	0.57598686	-0.5759859
222457_s_at	51474	LIMA1	Hs.525419	LIM domain and actin binding 1	2.2208612	0.5755596	-0.5755596
218532_s_at	54463	FAM134B	Hs.481704	family with sequence similarity 134, mem	2.2204208	0.57541656	-0.57541656
1562247_at	286058	LOC286058		hypothetical protein LOC286058	2.219803	0.5752158	-0.5752158
223058_at	83641	FAM107B	Hs.446315	family with sequence similarity 107, mem	2.2188473	0.5749054	-0.5749049
216243_s_at	3557	IL1RN	Hs.81134	interleukin 1 receptor antagonist	2.2126348	0.57288265	-0.57288265
213929_at	23086	EXPH5	Hs.28540	exophilin 5	2.2118874	0.572639	-0.572639
227088_at	8654	PDE5A	Hs.647971	phosphodiesterase 5A, cGMP-specific	2.211063	0.57237005	-0.57237005
225436_at	58489	FAM108C1	Hs.459072	family with sequence similarity 108, mem	2.2109942	0.57234764	-0.57234764
221664_s_at	50848	F11R	Hs.517293	F11 receptor	2.2077024	0.57127285	-0.57127285
224097_s_at	50848	F11R	Hs.517293	F11 receptor	2.2073083	0.5711441	-0.5711441
205660_at	8638	OASL	Hs.118633	2'-5'-oligoadenylate synthetase-like	2.207153	0.57109356	-0.5710931
227920_at	57673	BEND3	Hs.418045	BEN domain containing 3	2.206816	0.57098293	-0.5709834
1555800_at	151126	ZNF385B	Hs.655005	zinc finger protein 385B	2.2056572	0.5706043	-0.5706043
209873_s_at	11187	PKP3	Hs.534395	plakophilin 3	2.205354	0.57050514	-0.57050514
229887_at	259173	ALS2CL	Hs.517937	ALS2 C-terminal like	2.2047703	0.5703144	-0.57031393
225095_at	9517	SPTLC2	Hs.435661	Serine palmitoyltransferase, long chain ba	2.2032318	0.56981087	-0.5698104
205455_at	4486	MST1R	Hs.517973	macrophage stimulating 1 receptor (c-met	2.1977806	0.5680237	-0.5680237
226908_at	121227	LRIG3	Hs.724654	leucine-rich repeats and immunoglobulin-	2.1961648	0.56749344	-0.56749296
207096_at	6291	SAA4	Hs.654493	serum amyloid A4, constitutive	2.195268	0.5671983	-0.56719875
232666_at	4940	OAS3	Hs.528634	2'-5'-oligoadenylate synthetase 3, 100kDa	2.194163	0.5668354	-0.5668354
230006_s_at	258010	SVIP	Hs.349096	small VCP/p97-interacting protein	2.1922798	0.566216	-0.566216
1569044_at	55561	CDC42BPG	Hs.293590	CDC42 binding protein kinase gamma (D	2.1893082	0.5652375	-0.5652375
202016_at	4232	MEST	Hs.270978	mesoderm specific transcript homolog (m	2.1881318	0.56484985	-0.56484985
223949_at	64699	TMPRSS3	Hs.208600	transmembrane protease, serine 3	2.1859343	0.56412506	-0.56412506
218931_at	64284	RAB17	Hs.44278	RAB17, member RAS oncogene family	2.1842508	0.56356907	-0.56356955
229415_at	54205	CYCS	Hs.437060	cytochrome c, somatic	2.184116	0.5635247	-0.5635247
205375_at	4188	MDFI	Hs.520119	MyoD family inhibitor	2.1803164	0.56226873	-0.56226873
213506_at	2150	F2RL1	Hs.154299	coagulation factor II (thrombin) receptor-l	2.1791103	0.5618696	-0.5618696
1555827_at	57018	CCNL1	Hs.4859	Cyclin L1	2.1782355	0.5615797	-0.5615802
214519_s_at	6019	RLN2	Hs.127032	relaxin 2	2.1770103	0.5611739	-0.5611744
207768_at	1961	EGR4	Hs.3052	early growth response 4	2.176516	0.56101036	-0.56101036
1569114_at	100506847	LOC100506847		hypothetical LOC100506847	2.175968	0.5608287	-0.5608287
227970_at	80045	GPR157	Hs.632367	G protein-coupled receptor 157	2.1756647	0.5607281	-0.5607281
227642_at	29842	TFCP2L1	Hs.156471	transcription factor CP2-like 1	2.1752043	0.5605755	-0.5605755
213912_at	23329	TBC1D30	Hs.192492	TBC1 domain family, member 30	2.1750994	0.5605407	-0.5605407

235688_s_at	9618	TRAF4	Hs.8375	TNF receptor-associated factor 4	2.1744626	0.56032944	-0.56032944
229823_at	9699	RIMS2	Hs.655271	regulating synaptic membrane exocytosis	2.1686962	0.558414	-0.558414
226029_at	57216	VANGL2	Hs.99477	vang-like 2 (van gogh, Drosophila)	2.16668	0.5577431	-0.5577431
211056_s_at	6715	SRD5A1	Hs.552	steroid-5-alpha-reductase, alpha polypepti	2.1654315	0.5573273	-0.5573273
1554026_a_at	4651	MYO10	Hs.481720	myosin X	2.1654315	0.5573273	-0.5573273
204348_s_at	205	AK4	Hs.10862	adenylate kinase 4	2.164298	0.5569496	-0.5569496
210004_at	4973	OLR1	Hs.412484	oxidized low density lipoprotein (lectin-lil	2.1624944	0.5563483	-0.5563481
222946_s_at	79000	C1orf135	Hs.149305	chromosome 1 open reading frame 135	2.1612637	0.55593777	-0.5559373
236058_at	126695	C1orf172	Hs.188881	chromosome 1 open reading frame 172	2.1609528	0.5558338	-0.5558338
211756_at	5744	PTHLH	Hs.591159	parathyroid hormone-like hormone	2.1609104	0.5558195	-0.55581975
1555372_at	10018	BCL2L11	Hs.469658	BCL2-like 11 (apoptosis facilitator)	2.1606102	0.5557194	-0.5557194
225407_at	4155	MBP	Hs.551713	myelin basic protein	2.159283	0.5552759	-0.5552764
228462_at	153572	IRX2	Hs.282089	iroquois homeobox 2	2.1585944	0.5550461	-0.5550461
222940_at	6783	SULT1E1	Hs.479898	sulfotransferase family 1E, estrogen-prefe	2.1580951	0.5548792	-0.5548792
221485_at	9334	B4GALT5	Hs.370487	UDP-Gal:betaGlcNAc beta 1,4- galactosy	2.1558535	0.5541296	-0.5541296
240000_at			Hs.599720		2.1552436	0.5539255	-0.5539255
212510_at	23171	GPD1L	Hs.82432	glycerol-3-phosphate dehydrogenase 1-lik	2.149257	0.551919	-0.551919
203641_s_at	22837	COBLL1	Hs.470457	COBL-like 1	2.143342	0.54993105	-0.54993105
1558256_at	148189	LOC148189	Hs.565253	hypothetical LOC148189	2.1431394	0.54986286	-0.54986286
204347_at	205	AK4	Hs.10862	adenylate kinase 4	2.142553	0.54966545	-0.54966545
218647_s_at	79693	YRDC	Hs.301564	yrdC domain containing (E. coli)	2.141004	0.5491438	-0.5491438
213540_at	7923	HSD17B8	Hs.415058	hydroxysteroid (17-beta) dehydrogenase 8	2.1403985	0.5489397	-0.5489397
210582_s_at	3985	LIMK2	Hs.474596	LIM domain kinase 2	2.1397166	0.54870987	-0.54870987
35666_at	6405	SEMA3F	Hs.32981	sema domain, immunoglobulin domain (Ig	2.1393886	0.54859924	-0.54859924
202081_at	9592	IER2	Hs.501629	immediate early response 2	2.1357162	0.5473604	-0.54735947
207847_s_at	4582	MUC1	Hs.89603	mucin 1, cell surface associated	2.133254	0.54652786	-0.54652786
209099_x_at	182	JAG1	Hs.724464	jagged 1	2.1326506	0.5463238	-0.5463238
231399_at	117177	RAB3IP	Hs.258209	RAB3A interacting protein (rabin3)	2.1324024	0.54623985	-0.54623985
213237_at	400506	C16orf88	Hs.585209	chromosome 16 open reading frame 88	2.1313236	0.5458746	-0.5458751
221665_s_at	54869	EPS8L1	Hs.438862	EPS8-like 1	2.1312122	0.5458369	-0.5458374
232244_at	57462	KIAA1161	Hs.522083	KIAA1161	2.1311116	0.54580307	-0.54580307
238635_at	64417	C5orf28	Hs.558531	chromosome 5 open reading frame 28	2.1301613	0.5454812	-0.54548144
207147_at	1746	DLX2	Hs.419	distal-less homeobox 2	2.1296265	0.5453	-0.5453005
235907_at	55161	TMEM33	Hs.31082	transmembrane protein 33	2.1280935	0.54478073	-0.54478073
203108_at	9052	GPRC5A	Hs.631733	G protein-coupled receptor, family C, grou	2.127677	0.5446396	-0.5446396
213281_at	3725	JUN	Hs.723911	Jun oncogene	2.1273403	0.5445256	-0.54452515
203233_at	3566	IL4R	Hs.513457	interleukin 4 receptor	2.1215284	0.542552	-0.542552
223681_s_at	10207	INADL	Hs.478125	InaD-like (Drosophila)	2.1212506	0.5424576	-0.5424576
238692_at	121551	BTBD11	Hs.271272	BTB (POZ) domain containing 11	2.1191845	0.5417547	-0.5417545
218746_at	55080	TAPBPL	Hs.504597	TAP binding protein-like	2.1141849	0.540051	-0.5400505
213276_at	816	CAMK2B	Hs.351887	calcium/calmodulin-dependent protein kin	2.1120336	0.53931665	-0.5393162
213240_s_at	3851	KRT4	Hs.654610	keratin 4	2.1112108	0.5390353	-0.5390353
219250_s_at	23767	FLRT3	Hs.41296	fibronectin leucine rich transmembrane pr	2.110668	0.53884983	-0.53884983

205264_at	10849	CD3EAP	Hs.710495	CD3e molecule, epsilon associated protei	2.1101267	0.5386648	-0.5386648
219735_s_at	29842	TFCP2L1	Hs.156471	transcription factor CP2-like 1	2.1093094	0.5383854	-0.5383854
213394_at	23005	MAPKBP1	Hs.513661	mitogen-activated protein kinase binding	2.108659	0.5381632	-0.5381627
208092_s_at	81553	FAM49A	Hs.467769	family with sequence similarity 49, memb	2.1082222	0.53801346	-0.53801346
201032_at	10904	BLCAP	Hs.724405	bladder cancer associated protein	2.1075702	0.5377903	-0.5377903
242281_at	2752	GLUL	Hs.518525	glutamate-ammonia ligase	2.104711	0.5368109	-0.53681135
204060_s_at	5613 /// 5616	PRKX /// PRKY	Hs.721406	protein kinase, X-linked /// protein kinase	2.104535	0.5367508	-0.5367508
59705_at	51540	SCLY	Hs.709612	selenocysteine lyase	2.1040852	0.5365963	-0.5365968
230314_at			Hs.663929		2.1006997	0.5354347	-0.5354352
227756_at	145773	FAM81A	Hs.531168	family with sequence similarity 81, memb	2.100424	0.5353403	-0.5353403
229019_at	151126	ZNF385B	Hs.655005	zinc finger protein 385B	2.0988278	0.53479195	-0.53479195
204664_at	250	ALPP	Hs.284255	alkaline phosphatase, placental	2.098058	0.5345273	-0.5345273
203573_s_at	5875	RABGGTA	Hs.377992	Rab geranylgeranyltransferase, alpha subu	2.0974464	0.534317	-0.534317
200664_s_at	3337	DNAJB1	Hs.515210	DnaJ (Hsp40) homolog, subfamily B, mer	2.0972524	0.53425026	-0.53425026
224801_at	54602	NDFIP2	Hs.525093	Nedd4 family interacting protein 2	2.0954065	0.5336151	-0.5336151
229292_at	57669	EPB41L5	Hs.654802	erythrocyte membrane protein band 4.1 lil	2.095257	0.5335636	-0.5335636
212096_s_at	57509	MTUS1	Hs.7946	microtubule associated tumor suppressor	2.0950353	0.5334873	-0.5334873
1558154_at			Hs.670451		2.0943487	0.5332508	-0.5332508
230015_at	768206	PRCD	Hs.634380	progressive rod-cone degeneration	2.0935874	0.53298855	-0.53298855
201418_s_at	6659	SOX4	Hs.643910	SRY (sex determining region Y)-box 4	2.0921147	0.5324812	-0.5324807
1558703_at	113235	SLC46A1	Hs.724517	solute carrier family 46 (folate transporter	2.0919142	0.5324116	-0.53241205
1554006_a_at	3993	LLGL2	Hs.514477	lethal giant larvae homolog 2 (Drosophila	2.0916078	0.5323062	-0.5323062
205832_at	51200	CPA4	Hs.93764	carboxypeptidase A4	2.0904634	0.5319114	-0.5319114
235952_at			Hs.622213		2.0903265	0.53186417	-0.53186417
219623_at	79913	ACTR5	Hs.371585	ARP5 actin-related protein 5 homolog (ye	2.0886366	0.5312805	-0.531281
233463_at	166824	RASSF6	Hs.590920	Ras association (RalGDS/AF-6) domain f	2.087562	0.53090954	-0.53090954
237159_x_at	130340	AP1S3	Hs.632555	adaptor-related protein complex 1, sigma	2.08677	0.53063583	-0.53063583
229163_at	55450	CAMK2N1	Hs.197922	calcium/calmodulin-dependent protein kir	2.0839622	0.5296645	-0.5296645
227399_at	389136	VGLL3	Hs.724577	vestigial like 3 (Drosophila)	2.0837817	0.52960205	-0.52960205
219496_at	65124	ANKRD57	Hs.355455	ankyrin repeat domain 57	2.0813587	0.5287628	-0.5287628
209441_at	23221	RHOBTB2	Hs.372688	Rho-related BTB domain containing 2	2.0799723	0.52828217	-0.52828217
204654_s_at	7020	TFAP2A	Hs.519880	transcription factor AP-2 alpha (activating	2.0795956	0.5281515	-0.5281515
226926_at	93099	DMKN	Hs.417795	dermokine	2.0795805	0.52814627	-0.52814627
214193_s_at	27042	C1orf107	Hs.194754	chromosome 1 open reading frame 107	2.0794086	0.52808666	-0.52808666
207981_s_at	2104	ESRRG	Hs.444225	estrogen-related receptor gamma	2.078921	0.5279174	-0.5279176
228706_s_at	137075	CLDN23	Hs.183617	claudin 23	2.0786138	0.52781105	-0.5278106
211828_s_at	23043	TNIK	Hs.34024	TRAF2 and NCK interacting kinase	2.0755985	0.52676344	-0.5267639
210020_x_at	810	CALML3	Hs.239600	calmodulin-like 3	2.075221	0.5266323	-0.5266328
243754_at			Hs.561094		2.074255	0.5262966	-0.5262966
201847_at	3988	LIPA	Hs.643030	lipase A, lysosomal acid, cholesterol ester	2.0737736	0.5261297	-0.52612877
219821_s_at	54438	GFOD1	Hs.484686	glucose-fructose oxidoreductase domain c	2.072813	0.525795	-0.525795
215808_at	5655	KLK10	Hs.275464	kallikrein-related peptidase 10	2.0724034	0.5256524	-0.5256524
222994_at	25824	PRDX5	Hs.502823	peroxiredoxin 5	2.0699553	0.5248003	-0.52479935

228988_at	7552	ZNF711	Hs.326801	zinc finger protein 711	2.0684655	0.52428055	-0.5242803
217080_s_at	9455	HOMER2	Hs.578443	homer homolog 2 (Drosophila)	2.0673053	0.5238757	-0.5238757
242473_at	9618	TRAF4	Hs.8375	TNF receptor-associated factor 4	2.0665483	0.52361155	-0.52361155
228846_at	4084	MXD1	Hs.468908	MAX dimerization protein 1	2.0657542	0.5233345	-0.523334
224802_at	54602	NDFIP2	Hs.525093	Nedd4 family interacting protein 2	2.064537	0.52290916	-0.52290916
1555564_a_at	3426	CFI	Hs.312485	complement factor I	2.0638998	0.5226865	-0.5226865
218942_at	79837	PIP4K2C	Hs.144502	phosphatidylinositol-5-phosphate 4-kinase	2.0635002	0.52254677	-0.52254677
1556134_a_at	84002	B3GNT5	Hs.718506	UDP-GlcNAc:betaGal beta-1,3-N-acetylg	2.062656	0.5222516	-0.5222516
241950_at	23286	WWC1	Hs.484047	WW and C2 domain containing 1	2.0583231	0.5207348	-0.5207348
218511_s_at	55163	PNPO	Hs.631742	pyridoxamine 5'-phosphate oxidase	2.058258	0.5207119	-0.5207119
206653_at	10622	POLR3G	Hs.282387	polymerase (RNA) III (DNA directed) po	2.0557668	0.51983833	-0.51983833
1557521_a_at			Hs.595233		2.0502508	0.5179	-0.51790047
224458_at	84302	C9orf125	Hs.655738	chromosome 9 open reading frame 125	2.0502136	0.5178871	-0.5178871
219185_at	23408	SIRT5	Hs.567431	sirtuin 5	2.0472126	0.51683044	-0.51683044
243869_at			Hs.709930		2.0444016	0.5158396	-0.5158391
35820_at	2760	GM2A	Hs.483873	GM2 ganglioside activator	2.043943	0.51567745	-0.51567745
1559776_at			Hs.595177		2.0439322	0.51567364	-0.51567364
232533_at	79828	METTL8	Hs.135146	methyltransferase like 8	2.041959	0.514977	-0.514977
236101_at			Hs.444612		2.041486	0.5148096	-0.5148101
229139_at	56704	JPH1	Hs.657367	junctophilin 1	2.0410805	0.51466656	-0.51466656
213109_at	23043	TNIK	Hs.34024	TRAF2 and NCK interacting kinase	2.038363	0.51370525	-0.51370573
1568932_at			Hs.582552		2.03784	0.51352024	-0.5135205
220144_s_at	63926	ANKRD5	Hs.70903	ankyrin repeat domain 5	2.0377507	0.51348877	-0.51348877
210136_at	4155	MBP	Hs.551713	myelin basic protein	2.037045	0.5132389	-0.5132389
1557078_at	162394	SLFN5	Hs.709347	schlafen family member 5	2.0369387	0.51320124	-0.51320124
230361_at	727957	HEATR7A	Hs.443139	HEAT repeat containing 7A	2.036272	0.5129652	-0.5129652
37512_at	8630	HSD17B6	Hs.524513	hydroxysteroid (17-beta) dehydrogenase 6	2.03367	0.5120425	-0.512043
213693_s_at	4582	MUC1	Hs.89603	mucin 1, cell surface associated	2.0299985	0.5107393	-0.5107393
206919_at	2005	ELK4	Hs.497520	ELK4, ETS-domain protein (SRF accesso	2.0295584	0.5105829	-0.5105829
235200_at	93134	ZNF561	Hs.720081	zinc finger protein 561	2.0294015	0.51052713	-0.51052713
216251_s_at	23170	TTLL12	Hs.517670	tubulin tyrosine ligase-like family, membe	2.0285096	0.51021004	-0.51021004
212737_at	2760	GM2A	Hs.483873	GM2 ganglioside activator	2.0283165	0.5101414	-0.5101414
222392_x_at	64065	PERP	Hs.201446	PERP, TP53 apoptosis effector	2.0281918	0.5100975	-0.51009655
207045_at	55610	CCDC132	Hs.222282	coiled-coil domain containing 132	2.0278337	0.5099697	-0.5099697
242100_at	337876	CHSY3	Hs.213137	chondroitin sulfate synthase 3	2.0269465	0.50965405	-0.50965405
221291_at	80328	ULBP2	Hs.656778	UL16 binding protein 2	2.0243268	0.5087209	-0.50872135
206284_x_at	1212	CLTB	Hs.484241	clathrin, light chain B	2.0217934	0.50781727	-0.5078182
225450_at	154810	AMOTL1	Hs.503594	angiomotin like 1	2.0212467	0.5076227	-0.5076227
209667_at	8824	CES2	Hs.282975	carboxylesterase 2	2.0211346	0.50758266	-0.50758266
207291_at	79056	PRRG4	Hs.471695	proline rich Gla (G-carboxyglutamic acid)	2.0206056	0.50739384	-0.50739384
218816_at	55227	LRRC1	Hs.606493	leucine rich repeat containing 1	2.0205066	0.50735855	-0.50735855
227228_s_at	440193	CCDC88C	Hs.525536	coiled-coil domain containing 88C	2.020213	0.50725365	-0.50725365
225316_at	84879	MFSD2A	Hs.655177	major facilitator superfamily domain conta	2.0181468	0.5065155	-0.5065155

230792_at	158584	FAAH2	Hs.724714	fatty acid amide hydrolase 2	2.0180845	0.5064931	-0.50649357
219300_s_at	26047	CNTNAP2	Hs.655684	contactin associated protein-like 2	2.0174484	0.50626564	-0.5062661
205227_at	3556	IL1RAP	Hs.478673	interleukin 1 receptor accessory protein	2.0144014	0.5051756	-0.5051756
207826_s_at	3399	ID3	Hs.76884	inhibitor of DNA binding 3, dominant neg	2.012458	0.5044794	-0.5044794
241751_at	8481	OFD1	Hs.6483	oral-facial-digital syndrome 1	2.0118756	0.50427055	-0.50427055
210347_s_at	53335	BCL11A	Hs.370549	B-cell CLL/lymphoma 11A (zinc finger p	2.011676	0.504199	-0.504199
240331_at			Hs.658892		2.0113623	0.5040865	-0.5040865
243087_at	126820	WDR63	Hs.97933	WD repeat domain 63	2.0107243	0.5038576	-0.5038576
215425_at	10950	BTG3	Hs.473420	BTG family, member 3	2.0087075	0.5031338	-0.5031338
225140_at	51274	KLF3	Hs.298658	Kruppel-like factor 3 (basic)	2.0080497	0.50289726	-0.50289774
225726_s_at	57475	PLEKHH1	Hs.594236	pleckstrin homology domain containing, f	2.0076084	0.50273895	-0.50273895
226725_at			Hs.14691		2.007534	0.50271225	-0.50271225
225606_at	10018	BCL2L11	Hs.469658	BCL2-like 11 (apoptosis facilitator)	2.00742	0.50267124	-0.50267124
200965_s_at	3983	ABLIM1	Hs.438236	actin binding LIM protein 1	2.0060616	0.50218296	-0.50218296
218966_at	55930	MYO5C	Hs.487036	myosin VC	2.005595	0.5020151	-0.5020151
244546_at	54205	CYCS	Hs.437060	cytochrome c, somatic	2.0054598	0.5019665	-0.5019665
242417_at	283278	LOC283278		hypothetical protein LOC283278	2.0048075	0.5017319	-0.5017319
231001_at	387758	FIBIN	Hs.712718	fin bud initiation factor homolog (zebrafis	2.0029955	0.50107956	-0.50107956
220865_s_at	23590	PDSS1	Hs.558468	prenyl (decaprenyl) diphosphate synthase,	2.0024023	0.50086594	-0.50086594
214866_at	5329	PLAUR	Hs.466871	plasminogen activator, urokinase receptor	2.0020616	0.5007429	-0.5007434
227357_at	257397	TAB3	Hs.188256	TGF-beta activated kinase 1/MAP3K7 bi	2.0006347	0.5002289	-0.5002289
222449_at	56937	PMEPA1	Hs.517155	prostate transmembrane protein, androgen	-2.0013702	-0.500494	0.500494
212223_at	3423	IDS	Hs.460960	iduronate 2-sulfatase	-2.002299	-0.50082874	0.50082874
205756_s_at	2157	F8	Hs.654450	coagulation factor VIII, procoagulant com	-2.0024288	-0.5008755	0.5008755
211354_s_at	3953	LEPR	Hs.723178	leptin receptor	-2.0026035	-0.5009384	0.5009384
1553194_at	257194	NEGR1	Hs.146542	neuronal growth regulator 1	-2.0027986	-0.50100875	0.5010085
221031_s_at	81575	APOLD1	Hs.23388	apolipoprotein L domain containing 1	-2.0033107	-0.50119305	0.50119305
242186_x_at	23284	LPHN3	Hs.28391	latrophilin 3	-2.0036745	-0.50132394	0.5013242
212977_at	57007	CXCR7	Hs.471751	chemokine (C-X-C motif) receptor 7	-2.0038764	-0.5013969	0.50139666
232481_s_at	84189	SLITRK6	Hs.525105	SLIT and NTRK-like family, member 6	-2.003978	-0.5014334	0.5014334
201849_at	664	BNIP3	Hs.144873	BCL2/adenovirus E1B 19kDa interacting	-2.0042562	-0.5015335	0.5015335
235518_at	6546	SLC8A1	Hs.31961	solute carrier family 8 (sodium/calcium ex	-2.0046446	-0.5016732	0.5016732
235931_at	151194	FAM119A	Hs.664764	family with sequence similarity 119, mem	-2.0048552	-0.50174904	0.50174904
214830_at	145389	SLC38A6	Hs.200738	solute carrier family 38, member 6	-2.004903	-0.5017662	0.5017662
242918_at	4678	NASP	Hs.319334	Nuclear autoantigenic sperm protein (hist	-2.0073059	-0.50263023	0.50263023
227425_at	9185	REPS2	Hs.186810	RALBP1 associated Eps domain containi	-2.0080452	-0.50289583	0.50289583
233599_at	728061	LOC728061	Hs.655858	hCG2003663	-2.008219	-0.5029583	0.5029583
243041_s_at			Hs.705969		-2.0085456	-0.5030756	0.5030756
214890_s_at	25854	FAM149A	Hs.357025	family with sequence similarity 149, mem	-2.0092998	-0.50334644	0.50334644
227061_at	100506621	LOC100506621		hypothetical LOC100506621	-2.0093756	-0.5033736	0.5033736
201506_at	7045	TGFBI	Hs.369397	transforming growth factor, beta-induced,	-2.009547	-0.50343513	0.50343513
241739_at	55239	OGFOD1	Hs.231883	2-oxoglutarate and iron-dependent oxyger	-2.010251	-0.50368786	0.50368786
232451_at			Hs.216701		-2.0124428	-0.50447416	0.5044737

201367_s_at	678	ZFP36L2	Hs.503093	zinc finger protein 36, C3H type-like 2	-2.0126045	-0.50453186	0.50453186
201108_s_at	7057	THBS1	Hs.164226	thrombospondin 1	-2.013861	-0.504982	0.504982
1558569_at	100131541	LOC100131541	Hs.661972	Hypothetical LOC100131541	-2.0140438	-0.5050478	0.5050473
215369_at			Hs.649599		-2.0141451	-0.50508356	0.50508404
239571_at			Hs.674250		-2.0153604	-0.5055189	0.5055189
1556827_at	339929	LOC339929	Hs.596639	hypothetical LOC339929	-2.0155962	-0.5056033	0.5056033
236495_at			Hs.666584		-2.0157108	-0.5056443	0.5056443
244128_x_at	148979	GLIS1	Hs.306691	GLIS family zinc finger 1	-2.015726	-0.50564957	0.50565004
201125_s_at	3693	ITGB5	Hs.536663	integrin, beta 5	-2.0160053	-0.5057497	0.5057497
212221_x_at	3423	IDS	Hs.460960	iduronate 2-sulfatase	-2.0166345	-0.50597477	0.50597477
206002_at	10149	GPR64	Hs.146978	G protein-coupled receptor 64	-2.0175865	-0.50631523	0.50631523
201539_s_at	2273	FHL1	Hs.435369	four and a half LIM domains 1	-2.0180225	-0.50647116	0.50647116
239952_at	6935	ZEB1	Hs.124503	zinc finger E-box binding homeobox 1	-2.0182998	-0.5065701	0.50657034
224996_at	444	ASPH	Hs.332422	aspartate beta-hydroxylase	-2.0187204	-0.50672054	0.50672054
236962_at			Hs.598907		-2.021359	-0.5076628	0.5076628
204719_at	10351	ABCA8	Hs.58351	ATP-binding cassette, sub-family A (ABC	-2.0214057	-0.50767946	0.50767946
227892_at	5563	PRKAA2	Hs.437039	protein kinase, AMP-activated, alpha 2 ca	-2.0214057	-0.50767946	0.50767946
235172_at			Hs.655009		-2.0219703	-0.50788116	0.5078807
202378_s_at	54741	LEPROT	Hs.23581	leptin receptor overlapping transcript	-2.0222757	-0.5079899	0.5079899
1555892_s_at	253039	LOC253039	Hs.594170	hypothetical LOC253039	-2.0226755	-0.50813246	0.50813246
205730_s_at	22885	ABLIM3	Hs.49688	actin binding LIM protein family, membe	-2.0246098	-0.50882244	0.5088215
201279_s_at	1601	DAB2	Hs.481980	disabled homolog 2, mitogen-responsive p	-2.025235	-0.50904465	0.50904465
228986_at	114882	OSBPL8	Hs.430849	oxysterol binding protein-like 8	-2.0252376	-0.5090456	0.5090456
227179_at	27067	STAU2	Hs.561815	staufen, RNA binding protein, homolog 2	-2.0257144	-0.50921535	0.50921535
206652_at	9205	ZMYM5	Hs.530988	zinc finger, MYM-type 5	-2.0267081	-0.50956917	0.50956917
236521_at			Hs.95034		-2.0271623	-0.5097308	0.5097308
233543_s_at	84142	FAM175A	Hs.334772	family with sequence similarity 175, mem	-2.027492	-0.5098481	0.5098481
221478_at	665	BNIP3L	Hs.131226	BCL2/adenovirus E1B 19kDa interacting	-2.0275927	-0.5098839	0.5098839
208025_s_at	8091	HMGA2	Hs.505924	high mobility group AT-hook 2	-2.028539	-0.5102205	0.5102205
240728_at	5332	PLCB4	Hs.472101	Phospholipase C, beta 4	-2.0304682	-0.5109062	0.5109062
239121_at			Hs.687708		-2.0306675	-0.5109768	0.51097727
1558426_x_at	80228	ORAI2	Hs.363308	ORAI calcium release-activated calcium r	-2.0315719	-0.5112982	0.5112982
206042_x_at	6638 /// 8926	SNRPN /// SNU	Hs.555970	small nuclear ribonucleoprotein polypepti	-2.0316658	-0.51133156	0.51133156
212312_at	598	BCL2L1	Hs.516966	BCL2-like 1	-2.0325174	-0.5116339	0.5116339
209392_at	5168	ENPP2	Hs.190977	ectonucleotide pyrophosphatase/phosphod	-2.033441	-0.5119617	0.51196146
214438_at	3142	HLX	Hs.74870	H2.0-like homeobox	-2.0339153	-0.5121298	0.5121298
205198_s_at	538	ATP7A	Hs.496414	ATPase, Cu++ transporting, alpha polype	-2.0348613	-0.5124655	0.512465
210875_s_at	6935	ZEB1	Hs.124503	zinc finger E-box binding homeobox 1	-2.0350733	-0.51254034	0.51254034
1556339_a_at			Hs.662144		-2.0354607	-0.51267767	0.51267767
204392_at	8536	CAMK1	Hs.434875	calcium/calmodulin-dependent protein kir	-2.0357203	-0.5127697	0.5127697
223690_at	4053	LTBP2	Hs.512776	latent transforming growth factor beta bin	-2.036482	-0.5130396	0.5130396
238536_at			Hs.231861		-2.0369399	-0.5132017	0.5132017
242443_at	161436	EML5	Hs.724508	Echinoderm microtubule associated protei	-2.039233	-0.5140133	0.5140133

221556_at	8555	CDC14B	Hs.40582	CDC14 cell division cycle 14 homolog B	-2.039427	-0.51408195	0.51408195
207574_s_at	4616	GADD45B	Hs.110571	growth arrest and DNA-damage-inducible	-2.0409226	-0.5146103	0.51461124
212636_at	9444	QKI	Hs.510324	quaking homolog, KH domain RNA bindi	-2.0416818	-0.51487875	0.5148792
209135_at	444	ASPH	Hs.332422	aspartate beta-hydroxylase	-2.0418875	-0.5149517	0.5149517
205579_at	3269	HRH1	Hs.1570	histamine receptor H1	-2.0424383	-0.51514626	0.51514626
205447_s_at	7786	MAP3K12	Hs.713539	mitogen-activated protein kinase kinase k	-2.0426085	-0.51520634	0.51520634
216218_s_at	23228	PLCL2	Hs.723937	phospholipase C-like 2	-2.0453255	-0.51616526	0.51616526
242068_at			Hs.603603		-2.0459936	-0.5164008	0.5164008
230472_at	79192	IRX1	Hs.424156	iroquois homeobox 1	-2.0462277	-0.5164833	0.5164833
212043_at	10618	TGOLN2	Hs.593382	trans-golgi network protein 2	-2.0479665	-0.51709557	0.5170965
203827_at	55062	WIPI1	Hs.463964	WD repeat domain, phosphoinositide inte	-2.0486963	-0.51735306	0.51735306
1569583_at	2069	EREG	Hs.115263	epiregulin	-2.0488365	-0.5174022	0.51740265
223568_s_at	84513	PPAPDC1B	Hs.567619	phosphatidic acid phosphatase type 2 dom	-2.0502515	-0.51790047	0.51790047
238456_at	100289230	LOC100289230	Hs.552095	hypothetical LOC100289230	-2.050417	-0.51795864	0.51795864
244263_at					-2.050913	-0.51813316	0.51813316
214036_at	1946	EFNA5	Hs.288741	ephrin-A5	-2.051686	-0.51840496	0.51840496
240139_at			Hs.601985		-2.052746	-0.5187774	0.51877785
222444_at	51566	ARMCX3	Hs.592225	armadillo repeat containing, X-linked 3	-2.0542238	-0.51929665	0.51929665
233262_at			Hs.560364		-2.0542238	-0.51929665	0.51929665
227261_at	11278	KLF12	Hs.373857	Kruppel-like factor 12	-2.0542727	-0.5193138	0.5193138
242320_at			Hs.680761		-2.0548458	-0.51951504	0.51951504
226204_at	79680	C22orf29		chromosome 22 open reading frame 29	-2.0549407	-0.5195484	0.5195484
213836_s_at	55062	WIPI1	Hs.463964	WD repeat domain, phosphoinositide inte	-2.0549707	-0.5195589	0.5195589
210282_at	7750	ZMYM2	Hs.644041	zinc finger, MYM-type 2	-2.0589485	-0.52095413	0.52095366
238317_x_at					-2.0595896	-0.5211787	0.52117825
225602_at	152007	GLIPR2	Hs.493819	GLI pathogenesis-related 2	-2.0609033	-0.5216384	0.5216384
238774_at					-2.0610456	-0.52168846	0.521688
1556416_s_at			Hs.684703		-2.061086	-0.5217023	0.5217023
238751_at			Hs.481342		-2.0624583	-0.52218246	0.52218246
238712_at			Hs.656379		-2.062639	-0.5222459	0.5222454
227826_s_at			Hs.481342		-2.0638998	-0.5226865	0.5226865
224444_s_at	84791	C1orf97		chromosome 1 open reading frame 97	-2.0651622	-0.52312756	0.52312756
229576_s_at	6926	TBX3	Hs.129895	T-box 3	-2.0652707	-0.5231657	0.5231652
201468_s_at	1728	NQO1	Hs.406515	NAD(P)H dehydrogenase, quinone 1	-2.0656838	-0.5233097	0.5233097
1316_at	7067	THRA	Hs.724	thyroid hormone receptor, alpha (erythrob	-2.0659711	-0.5234103	0.52340984
239065_at			Hs.596765		-2.0665455	-0.5236106	0.5236106
238129_s_at			Hs.597264		-2.067327	-0.52388334	0.52388334
228568_at	145781	GCOM1	Hs.437256	GRINL1A complex locus	-2.0673456	-0.52388954	0.52389
226043_at	26086	GPSM1	Hs.239370	G-protein signaling modulator 1	-2.0674405	-0.5239229	0.5239229
219316_s_at	55640	FLVCR2	Hs.509966	feline leukemia virus subgroup C cellular	-2.067858	-0.52406836	0.52406883
240018_at			Hs.513672		-2.069506	-0.5246434	0.52464294
236545_at			Hs.662933		-2.0709805	-0.525157	0.525157
240788_at			Hs.656597		-2.0714748	-0.5253291	0.5253291

225562_at	22821	RASA3	Hs.593075	RAS p21 protein activator 3	-2.0724623	-0.5256729	0.5256729
238736_at	5980	REV3L	Hs.232021	REV3-like, catalytic subunit of DNA poly	-2.077584	-0.5274534	0.5274534
214505_s_at	2273	FHL1	Hs.435369	four and a half LIM domains 1	-2.0779433	-0.52757835	0.5275779
244407_at	51302	CYP39A1	Hs.387367	cytochrome P450, family 39, subfamily A	-2.0784454	-0.5277524	0.5277524
241685_x_at			Hs.200938		-2.0793605	-0.52807	0.52807
211432_s_at	7301	TYRO3	Hs.381282	TYRO3 protein tyrosine kinase	-2.080789	-0.5285654	0.5285654
213030_s_at	5362	PLXNA2	Hs.497626	plexin A2	-2.081144	-0.52868843	0.52868843
225747_at	93058	COQ10A	Hs.4096	coenzyme Q10 homolog A (S. cerevisiae)	-2.0812554	-0.52872705	0.52872705
229497_at	348094	ANKDD1A	Hs.207157	ankyrin repeat and death domain containi	-2.0814753	-0.5288031	0.52880335
229254_at	148808	MFSD4	Hs.567714	major facilitator superfamily domain conta	-2.0815845	-0.528841	0.528841
241782_at	10529	NEBL	Hs.5025	nebulette	-2.0823627	-0.52911043	0.5291109
202620_s_at	5352	PLOD2	Hs.477866	procollagen-lysine, 2-oxoglutarate 5-dioxy	-2.0843492	-0.5297985	0.5297985
236322_at			Hs.661081		-2.0845408	-0.5298648	0.5298648
213309_at	23228	PLCL2	Hs.723937	phospholipase C-like 2	-2.0854304	-0.53017235	0.5301728
242908_x_at			Hs.23187		-2.0859978	-0.5303688	0.5303688
221011_s_at	81606	LBH	Hs.567598	limb bud and heart development homolog	-2.0867453	-0.53062725	0.53062725
221004_s_at	81618	ITM2C	Hs.111577	integral membrane protein 2C	-2.087035	-0.5307274	0.5307274
236520_at			Hs.655785		-2.087562	-0.53090954	0.53090954
226665_at	130872	AHSA2	Hs.655602	AHA1, activator of heat shock 90kDa pro	-2.0894341	-0.5315561	0.5315561
213555_at	112611	RWDD2A	Hs.709686	RWD domain containing 2A	-2.090268	-0.53184414	0.53184366
242879_x_at					-2.091202	-0.5321665	0.532166
244117_at			Hs.657231		-2.0937002	-0.53302765	0.5330272
224549_x_at					-2.0951352	-0.53352165	0.53352165
1553768_a_at	285761	DCBLD1	Hs.658304	discoidin, CUB and LCCL domain contai	-2.0960267	-0.53382826	0.53382874
225685_at	10602	CDC42EP3	Hs.369574	CDC42 effector protein (Rho GTPase bin	-2.097341	-0.5342808	0.5342808
215322_at			Hs.671978		-2.0977995	-0.5344386	0.53443813
209539_at	9459	ARHGEF6	Hs.522795	Rac/Cdc42 guanine nucleotide exchange f	-2.0996919	-0.53508854	0.535089
220750_s_at	64175	LEPRE1	Hs.720014	leucine proline-enriched proteoglycan (lep	-2.100383	-0.5353265	0.535326
220609_at	202181	LOC202181		hypothetical LOC202181	-2.1008308	-0.53548	0.53548
215469_at			Hs.137567		-2.1018727	-0.53583765	0.53583765
241710_at	728819	LOC728819	Hs.602756	hCG1645220	-2.1020145	-0.5358863	0.5358863
202125_s_at	66008	TRAK2	Hs.152774	trafficking protein, kinesin binding 2	-2.1031666	-0.5362816	0.5362816
226267_at	122953	JDP2	Hs.196482	Jun dimerization protein 2	-2.104483	-0.53673315	0.5367327
1558683_a_at	8091	HMGA2	Hs.505924	high mobility group AT-hook 2	-2.1047103	-0.5368109	0.5368109
223438_s_at	5465	PPARA	Hs.103110	peroxisome proliferator-activated receptor	-2.1047251	-0.53681564	0.5368161
227080_at	90874	ZNF697	Hs.381105	zinc finger protein 697	-2.1049635	-0.53689766	0.53689766
237387_at			Hs.673912		-2.1050847	-0.53693914	0.53693914
219610_at	64283	RGNEF	Hs.482521	190 kDa guanine nucleotide exchange fac	-2.1056428	-0.53713036	0.53713036
205803_s_at	7220	TRPC1	Hs.250687	transient receptor potential cation channel	-2.1062171	-0.5373273	0.5373268
1563745_a_at	283050	LOC283050	Hs.309176	hypothetical LOC283050	-2.1076133	-0.5378051	0.5378051
242438_at	171023	ASXL1	Hs.374043	additional sex combs like 1 (Drosophila)	-2.1089303	-0.5382557	0.5382557
221078_s_at	55704	CCDC88A	Hs.292925	coiled-coil domain containing 88A	-2.1100905	-0.5386524	0.5386524
1559910_at			Hs.655648		-2.1101093	-0.5386586	0.5386591

237577_at	57092	PCNP	Hs.275865	PEST proteolytic signal containing nuclea	-2.1105843	-0.5388212	0.5388212
228160_at	339290	LOC339290		hypothetical LOC339290	-2.1106575	-0.5388465	0.538846
235729_at	84874	ZNF514	Hs.655109	zinc finger protein 514	-2.110924	-0.5389371	0.53893757
1562775_at	83594	NUDT12	Hs.434289	nudix (nucleoside diphosphate linked moi	-2.1109865	-0.5389588	0.53895855
213012_at	4734	NEDD4	Hs.1565	neural precursor cell expressed, developm	-2.1113489	-0.5390825	0.5390825
220744_s_at	55764	IFT122	Hs.655284	intraflagellar transport 122 homolog (Chla	-2.1138291	-0.5399294	0.5399294
213763_at	28996	HIPK2	Hs.724392	homeodomain interacting protein kinase 2	-2.1144552	-0.540143	0.540143
211352_s_at	8202	NCOA3	Hs.592142	nuclear receptor coactivator 3	-2.1149669	-0.54031754	0.54031754
235537_at	54940	OCIAD1	Hs.518750	OCIA domain containing 1	-2.1151786	-0.54039	0.54038954
205379_at	874	CBR3	Hs.154510	carbonyl reductase 3	-2.1153276	-0.54044056	0.54044056
236795_at			Hs.634209		-2.1160276	-0.54067945	0.540679
215407_s_at	23245	ASTN2	Hs.601562	astrotactin 2	-2.1162732	-0.5407629	0.5407629
237189_at	100506360	LOC100506360		hypothetical LOC100506360	-2.1162739	-0.5407634	0.5407629
214321_at	4856	NOV	Hs.235935	nephroblastoma overexpressed gene	-2.116772	-0.54093313	0.54093266
218546_at	79762	C1orf115	Hs.519839	chromosome 1 open reading frame 115	-2.1187506	-0.5416069	0.5416069
219654_at	9200	PTPLA	Hs.114062	protein tyrosine phosphatase-like (proline	-2.1189916	-0.5416889	0.5416889
1568605_at	8629	JRK	Hs.535903	jerky homolog (mouse)	-2.120205	-0.54210186	0.54210186
33148_at	51663	ZFR	Hs.435231	zinc finger RNA binding protein	-2.121405	-0.54251003	0.54251003
223870_at	10517	FBXW10	Hs.592128	F-box and WD repeat domain containing	-2.122227	-0.54278946	0.54278946
224771_at	89796	NAV1	Hs.585374	neuron navigator 1	-2.122934	-0.5430298	0.5430298
224410_s_at	64327	LMBR1	Hs.209989	limb region 1 homolog (mouse)	-2.123588	-0.543252	0.543252
1559528_at	100129917	LOC100129917	Hs.711523	hypothetical LOC100129917	-2.1248238	-0.5436716	0.5436716
214464_at	8476	CDC42BPA	Hs.35433	CDC42 binding protein kinase alpha (DM	-2.125723	-0.5439768	0.5439768
223041_at	83692	CD99L2	Hs.522805	CD99 molecule-like 2	-2.126673	-0.5442991	0.5442991
205234_at	9122	SLC16A4	Hs.351306	solute carrier family 16, member 4 (mono	-2.1269796	-0.5444031	0.5444031
209465_x_at	5764	PTN	Hs.371249	pleiotrophin	-2.127244	-0.5444927	0.5444927
214999_s_at	9727	RAB11FIP3	Hs.531642	RAB11 family interacting protein 3 (class	-2.12731	-0.54451513	0.54451513
205500_at	727	C5	Hs.494997	complement component 5	-2.1282833	-0.5448451	0.5448451
220825_s_at	55243	KIRREL	Hs.609291	kin of IRRE like (Drosophila)	-2.128289	-0.544847	0.544847
202701_at	649	BMP1	Hs.1274	bone morphogenetic protein 1	-2.12898	-0.54508114	0.54508114
236610_at			Hs.591763		-2.1322014	-0.54617167	0.54617214
219682_s_at	6926	TBX3	Hs.129895	T-box 3	-2.1322114	-0.546175	0.5461755
200756_x_at	813	CALU	Hs.724446	calumenin	-2.1324306	-0.5462494	0.5462494
243149_at			Hs.669156		-2.132931	-0.54641867	0.54641867
229831_at	5067	CNTN3	Hs.12723	contactin 3 (plasmacytoma associated)	-2.133835	-0.5467243	0.5467243
244181_at					-2.1357303	-0.5473647	0.5473647
214068_at	146227	BEAN1	Hs.97805	brain expressed, associated with NEDD4,	-2.1385741	-0.5483246	0.5483246
238716_at	619423	FAM85A		family with sequence similarity 85, memb	-2.1386766	-0.5483589	0.5483594
227828_s_at	84141	FAM176A	Hs.302346	family with sequence similarity 176, mem	-2.1388624	-0.54842186	0.54842186
205876_at	3977	LIFR	Hs.133421	leukemia inhibitory factor receptor alpha	-2.1400447	-0.5488205	0.5488205
202832_at	9648	GCC2	Hs.436505	GRIP and coiled-coil domain containing 2	-2.1402485	-0.54888916	0.54888916
239649_at					-2.1406822	-0.5490351	0.54903555
239830_at					-2.1438253	-0.55009365	0.55009365

1564139_at	144571	LOC144571	Hs.592432	hypothetical LOC144571	-2.1439216	-0.5501261	0.5501261
225224_at	140688	C20orf112	Hs.516978	chromosome 20 open reading frame 112	-2.1442902	-0.55025005	0.55025005
230364_at	56994	CHPT1	Hs.293077	choline phosphotransferase 1	-2.1453288	-0.5505996	0.5505991
225045_at	55704	CCDC88A	Hs.292925	coiled-coil domain containing 88A	-2.1454082	-0.5506263	0.5506258
204642_at	1901	S1PR1	Hs.154210	sphingosine-1-phosphate receptor 1	-2.1463954	-0.55095816	0.5509577
211966_at	1284	COL4A2	Hs.508716	collagen, type IV, alpha 2	-2.146726	-0.5510688	0.55106926
242024_at			Hs.656132		-2.1468132	-0.55109835	0.55109835
230860_at	84984	C3orf34	Hs.282800	chromosome 3 open reading frame 34	-2.1468651	-0.5511155	0.551116
214048_at	8930	MBD4	Hs.35947	methyl-CpG binding domain protein 4	-2.147195	-0.5512266	0.5512266
1555349_a_at	3689	ITGB2	Hs.375957	integrin, beta 2 (complement component 3	-2.1488798	-0.55179214	0.5517926
223441_at	26503	SLC17A5	Hs.597422	solute carrier family 17 (anion/sugar trans	-2.1514516	-0.5526552	0.5526552
208763_s_at	1831	TSC22D3	Hs.522074	TSC22 domain family, member 3	-2.152934	-0.5531521	0.5531521
213103_at	90627	STARD13	Hs.507704	StAR-related lipid transfer (START) dom	-2.1531646	-0.55322933	0.55322933
202718_at	3485	IGFBP2	Hs.438102	insulin-like growth factor binding protein	-2.1544397	-0.5536561	0.5536566
228618_at	375033	PEAR1	Hs.142003	platelet endothelial aggregation receptor 1	-2.1552436	-0.5539255	0.5539255
202923_s_at	2729	GCLC	Hs.654465	glutamate-cysteine ligase, catalytic subun	-2.1552522	-0.5539284	0.5539284
230503_at			Hs.656734		-2.1559477	-0.5541611	0.5541611
222866_s_at	55640	FLVCR2	Hs.509966	feline leukemia virus subgroup C cellular	-2.1567338	-0.5544238	0.5544243
222771_s_at	50804	MYEF2	Hs.6638	myelin expression factor 2	-2.1568265	-0.5544553	0.5544548
232021_at	283464	GXYLT1	Hs.259347	glucoside xylosyltransferase 1	-2.1571772	-0.5545726	0.5545721
227341_at	222389	BEND7	Hs.498740	BEN domain containing 7	-2.1571915	-0.55457735	0.5545769
1562288_at			Hs.535143		-2.1573348	-0.55462503	0.55462503
224002_s_at	51661	FKBP7	Hs.410378	FK506 binding protein 7	-2.1577163	-0.5547528	0.55475235
212107_s_at	1660	DHX9	Hs.191518	DEAH (Asp-Glu-Ala-His) box polypeptic	-2.157769	-0.55477	0.55477047
238419_at	90102	PHLDB2	Hs.477114	pleckstrin homology-like domain, family I	-2.1581962	-0.55491304	0.55491304
243541_at	133396	IL31RA	Hs.55378	interleukin 31 receptor A	-2.1601946	-0.5555806	0.5555806
216048_s_at	22836	RHOBTB3	Hs.445030	Rho-related BTB domain containing 3	-2.1609116	-0.55582	0.55582
226995_at	642852	LOC642852	Hs.11637	hypothetical LOC642852	-2.1616344	-0.55606127	0.55606127
225290_at	55500	ETNK1	Hs.29464	ethanolamine kinase 1	-2.161713	-0.5560875	0.5560875
1554250_s_at	375593	TRIM73	Hs.661254	tripartite motif-containing 73	-2.1618345	-0.556128	0.556128
227148_at	130271	PLEKHH2	Hs.164162	pleckstrin homology domain containing, f	-2.1625175	-0.55635595	0.55635595
202761_s_at	23224	SYNE2	Hs.525392	spectrin repeat containing, nuclear envelo	-2.1633098	-0.55661964	0.5566206
212335_at	2799	GNS	Hs.334534	glucosamine (N-acetyl)-6-sulfatase	-2.164092	-0.55688095	0.55688095
235959_at			Hs.709640		-2.166491	-0.55768013	0.55768013
238206_at	59350	RXFP1	Hs.591686	relaxin/insulin-like family peptide recepto	-2.167281	-0.55794334	0.55794287
241395_at	4817	NIT1	Hs.517342	nitrilase 1	-2.1691062	-0.55855036	0.55855036
204646_at	1806	DPYD	Hs.335034	dihydropyrimidine dehydrogenase	-2.1692224	-0.558589	0.558589
228643_at			Hs.503429		-2.1693695	-0.5586376	0.5586381
217878_s_at	996	CDC27	Hs.463295	cell division cycle 27 homolog (S. cerevis	-2.1701174	-0.5588865	0.5588865
1553158_at	84984	C3orf34	Hs.282800	chromosome 3 open reading frame 34	-2.1703584	-0.55896664	0.55896664
241844_x_at	80008	TMEM156	Hs.374147	transmembrane protein 156	-2.1724842	-0.55967283	0.55967283
224229_s_at	10000	AKT3	Hs.498292	v-akt murine thymoma viral oncogene hor	-2.1727147	-0.5597491	0.5597496
209094_at	23576	DDAH1	Hs.724433	dimethylarginine dimethylaminohydrolase	-2.173267	-0.5599327	0.5599327

242865_at					-2.1742384	-0.56025505	0.56025505
231136_at	407032	MIR30C2		microRNA 30c-2	-2.1773932	-0.56130123	0.56130075
240485_at					-2.1782348	-0.5615797	0.5615797
203066_at	51363	CHST15	Hs.287537	carbohydrate (N-acetylgalactosamine 4-su	-2.1784334	-0.5616455	0.5616455
203300_x_at	8905	AP1S2	Hs.653504	adaptor-related protein complex 1, sigma	-2.1785572	-0.5616865	0.5616865
244697_at					-2.1785572	-0.5616865	0.5616865
217641_at	64582	GPR135	Hs.647573	G protein-coupled receptor 135	-2.1788108	-0.56177044	0.56177044
242028_at	163051	ZNF709	Hs.631623	zinc finger protein 709	-2.180087	-0.5621929	0.5621929
232293_at	254251	LCORL	Hs.446201	ligand dependent nuclear receptor corepre	-2.1808858	-0.5624571	0.5624571
202391_at	10409	BASP1	Hs.201641	brain abundant, membrane attached signal	-2.1809738	-0.56248665	0.5624857
1560703_at	201229	C17orf108	Hs.724502	Chromosome 17 open reading frame 108	-2.1829326	-0.56313396	0.5631337
225420_at	57678	GPAM	Hs.42586	glycerol-3-phosphate acyltransferase, mito	-2.1836755	-0.5633793	0.5633793
231130_at	51661	FKBP7	Hs.410378	FK506 binding protein 7	-2.1851752	-0.5638747	0.56387424
1556619_at	729993	SHISA9	Hs.724830	shisa homolog 9 (Xenopus laevis)	-2.185423	-0.56395626	0.56395626
231067_s_at	9590	AKAP12	Hs.371240	A kinase (PRKA) anchor protein 12	-2.1859314	-0.5641241	0.5641241
206819_at	29774	POM121L9P	Hs.534980	POM121 membrane glycoprotein-like 9, p	-2.1874506	-0.56462526	0.56462526
203758_at	1519	CTSO	Hs.75262	cathepsin O	-2.1885087	-0.5649743	0.56497383
223888_s_at	51520	LARS	Hs.432674	leucyl-tRNA synthetase	-2.189283	-0.56522894	0.5652294
234282_at			Hs.543773		-2.189692	-0.5653641	0.5653639
239519_at			Hs.687190		-2.1912048	-0.5658622	0.5658622
225656_at	114327	EFHC1	Hs.403171	EF-hand domain (C-terminal) containing	-2.19132	-0.5659003	0.56589985
223286_at	23587	C17orf81	Hs.417029	chromosome 17 open reading frame 81	-2.192719	-0.5663605	0.5663605
241400_at					-2.1928554	-0.5664053	0.5664053
215242_at	5279	PIGC	Hs.188456	phosphatidylinositol glycan anchor biosyn	-2.1937468	-0.56669855	0.56669855
212609_s_at	10000	AKT3	Hs.498292	V-akt murine thymoma viral oncogene ho	-2.1944127	-0.5669174	0.5669174
228345_at	53344	CHIC1	Hs.496323	cysteine-rich hydrophobic domain 1	-2.1949625	-0.56709814	0.56709814
238447_at	27303	RBMS3	Hs.696468	RNA binding motif, single stranded intera	-2.1964908	-0.56760025	0.56760025
206665_s_at	598	BCL2L1	Hs.516966	BCL2-like 1	-2.1973505	-0.56788254	0.56788254
236649_at	56986	DTWD1	Hs.127432	DTW domain containing 1	-2.198693	-0.56832314	0.56832314
210191_s_at	10745	PHTF1	Hs.655824	putative homeodomain transcription facto	-2.1991343	-0.5684676	0.5684681
217599_s_at	29969	MDFIC	Hs.427236	MyoD family inhibitor domain containing	-2.1996796	-0.56864643	0.5686469
201626_at	3638	INSIG1	Hs.520819	insulin induced gene 1	-2.2003682	-0.56887245	0.56887245
237813_at	5094	PCBP2	Hs.546271	poly(rC) binding protein 2	-2.2009747	-0.5690713	0.5690713
206675_s_at	6498	SKIL	Hs.581632	SKI-like oncogene	-2.202158	-0.56945896	0.56945896
237716_at					-2.2022066	-0.5694747	0.5694752
233109_at	1303	COL12A1	Hs.101302	Collagen, type XII, alpha 1	-2.202324	-0.5695133	0.5695133
206135_at	9705	ST18	Hs.655499	suppression of tumorigenicity 18 (breast c	-2.2054298	-0.57052994	0.57052994
207290_at	5362	PLXNA2	Hs.497626	plexin A2	-2.2056456	-0.5706005	0.5706005
210194_at	22925	PLA2R1	Hs.410477	phospholipase A2 receptor 1, 180kDa	-2.2056708	-0.57060885	0.5706086
207020_at	11077	HSF2BP	Hs.406157	heat shock transcription factor 2 binding p	-2.2059257	-0.57069206	0.57069206
43511_s_at			Hs.568928		-2.2078922	-0.57133484	0.57133484
213416_at	3676	ITGA4	Hs.440955	integrin, alpha 4 (antigen CD49D, alpha 4	-2.2089272	-0.5716729	0.5716729
206746_at	631	BFSP1	Hs.129702	beaded filament structural protein 1, filens	-2.2091548	-0.5717473	0.5717473

220512_at	10395	DLC1	Hs.134296	deleted in liver cancer 1	-2.209999	-0.5720229	0.5720229
204796_at	2009	EML1	Hs.12451	echinoderm microtubule associated protei	-2.2105982	-0.5722184	0.5722184
220441_at	79962	DNAJC22	Hs.659300	DnaJ (Hsp40) homolog, subfamily C, mer	-2.2110527	-0.5723667	0.5723667
239058_at	2303	FOXC2	Hs.436448	Forkhead box C2 (MFH-1, mesenchyme f	-2.2120965	-0.5727072	0.5727072
243368_at			Hs.598905		-2.2128499	-0.57295275	0.57295275
204818_at	3294	HSD17B2	Hs.162795	hydroxysteroid (17-beta) dehydrogenase 2	-2.2139845	-0.5733228	0.5733223
229907_at			Hs.369009		-2.214944	-0.5736351	0.5736351
219387_at	55704	CCDC88A	Hs.292925	coiled-coil domain containing 88A	-2.216822	-0.5742464	0.5742464
212788_x_at	2512	FTL	Hs.433670	ferritin, light polypeptide	-2.2170563	-0.5743227	0.5743227
203188_at	11041	B3GNT1	Hs.8526	UDP-GlcNAc:betaGal beta-1,3-N-acetylg	-2.2181382	-0.5746746	0.5746746
212336_at	2036	EPB41L1	Hs.724416	erythrocyte membrane protein band 4.1-lil	-2.2192624	-0.57503986	0.57504034
220326_s_at	55701	ARHGEF40	Hs.35125	Rho guanine nucleotide exchange factor (	-2.2204657	-0.57543135	0.57543087
1566557_at	440465	FLJ90757	Hs.448889	hypothetical LOC440465	-2.2207203	-0.57551384	0.57551384
1569472_s_at	7267	TTC3	Hs.368214	tetratricopeptide repeat domain 3	-2.2248461	-0.5768528	0.5768528
202479_s_at	28951	TRIB2	Hs.467751	tribbles homolog 2 (Drosophila)	-2.2250211	-0.57690954	0.57690954
243827_at			Hs.601123		-2.22582	-0.57716846	0.57716846
212311_at	23231	SEL1L3	Hs.479384	sel-1 suppressor of lin-12-like 3 (C. elega	-2.2296576	-0.5784111	0.5784111
209867_s_at	23284	LPHN3	Hs.570770	latrophilin 3	-2.23247	-0.57932043	0.57932043
1554199_at	5800	PTPRO	Hs.160871	protein tyrosine phosphatase, receptor typ	-2.233059	-0.5795107	0.5795107
227115_at	100506870 /// 10	LOC100506870 /// LOC100509475		hypothetical LOC100506870 /// hypotheti	-2.2339692	-0.5798044	0.5798049
221911_at	2115	ETV1	Hs.22634	ets variant 1	-2.2342846	-0.57990646	0.57990646
236712_at			Hs.55185		-2.2346807	-0.5800345	0.58003426
233825_s_at	83692	CD99L2	Hs.522805	CD99 molecule-like 2	-2.2365158	-0.5806265	0.5806265
212624_s_at	1123	CHN1	Hs.380138	chimerin (chimaerin) 1	-2.2372255	-0.58085537	0.58085537
216813_at					-2.2389596	-0.5814142	0.5814142
217858_s_at	51566	ARMCX3	Hs.592225	armadillo repeat containing, X-linked 3	-2.2404919	-0.58190775	0.58190775
203441_s_at	1000	CDH2	Hs.464829	cadherin 2, type 1, N-cadherin (neuronal)	-2.2426434	-0.5826001	0.5826001
217989_at	51170	HSD17B11	Hs.594923	hydroxysteroid (17-beta) dehydrogenase 1	-2.2428198	-0.58265686	0.58265686
244433_at					-2.2457669	-0.58360386	0.58360434
228628_at	653464	SRGAP2P1	Hs.523529	SLIT-ROBO Rho GTPase activating prot	-2.2475393	-0.5841732	0.5841732
203184_at	2201	FBN2	Hs.519294	fibrillin 2	-2.2479496	-0.5843048	0.5843048
236852_at	286151	FBXO43	Hs.339577	F-box protein 43	-2.248232	-0.5843954	0.5843954
231182_at	7456	WIPF1	Hs.128067	WAS/WASL interacting protein family, n	-2.2510931	-0.58531284	0.58531284
203402_at	8514	KCNAB2	Hs.440497	potassium voltage-gated channel, shaker-r	-2.2519927	-0.58560085	0.58560133
238918_at			Hs.276976		-2.2520187	-0.58560944	0.58560944
1562472_at					-2.2528954	-0.58589005	0.5858903
1557118_a_at			Hs.594897		-2.2529376	-0.58590364	0.58590364
1552799_at	203062	TSNARE1	Hs.370931	t-SNARE domain containing 1	-2.2542915	-0.5863371	0.5863371
220197_at	50617	ATP6V0A4	Hs.98967	ATPase, H+ transporting, lysosomal V0 s	-2.2545874	-0.586432	0.5864315
210519_s_at	1728	NQO1	Hs.406515	NAD(P)H dehydrogenase, quinone 1	-2.255511	-0.58672714	0.58672714
228392_at	55900	ZNF302	Hs.436350	Zinc finger protein 302	-2.2564795	-0.5870371	0.5870366
208747_s_at	716	C1S	Hs.458355	complement component 1, s subcomponen	-2.258851	-0.5877948	0.5877943
238939_at	118	ADD1	Hs.183706	adducin 1 (alpha)	-2.259371	-0.5879605	0.5879607

242871_at	54852	PAQR5	Hs.591096	progestin and adipoQ receptor family mer	-2.2624457	-0.5889416	0.5889416
230064_at			Hs.650577		-2.2629378	-0.58909845	0.58909845
1558280_s_at	9411	ARHGAP29	Hs.483238	Rho GTPase activating protein 29	-2.2635078	-0.5892801	0.5892801
1555765_a_at	2786	GNG4	Hs.159711	guanine nucleotide binding protein (G pro	-2.2661576	-0.59012413	0.59012413
205535_s_at	5099	PCDH7	Hs.479439	protocadherin 7	-2.2668123	-0.5903325	0.5903325
236408_at			Hs.674006		-2.268142	-0.59075546	0.59075546
221765_at	7357	UGCG	Hs.304249	UDP-glucose ceramide glucosyltransferas	-2.2689188	-0.59100246	0.59100246
209732_at	9976	CLEC2B	Hs.85201	C-type lectin domain family 2, member B	-2.2696657	-0.5912399	0.5912399
210281_s_at	7750	ZMYM2	Hs.644041	zinc finger, MYM-type 2	-2.2699838	-0.591341	0.591341
230712_at	55672	NBPF1	Hs.467587	neuroblastoma breakpoint family, membe	-2.2722297	-0.59205437	0.59205437
222453_at	79901	CYBRD1	Hs.723152	cytochrome b reductase 1	-2.272455	-0.5921259	0.5921259
238289_at					-2.2725017	-0.5921407	0.5921407
239540_at					-2.2750673	-0.59295464	0.59295464
241853_at			Hs.596355		-2.2768157	-0.5935087	0.5935087
203348_s_at	2119	ETV5	Hs.43697	ets variant 5	-2.2783482	-0.59399414	0.59399414
235216_at	114799	ESCO1	Hs.464733	establishment of cohesion 1 homolog 1 (S	-2.2789433	-0.5941825	0.5941825
1557507_at			Hs.638073		-2.2790985	-0.5942316	0.5942316
230657_at			Hs.668532		-2.281039	-0.5948453	0.5948458
244271_at			Hs.270314		-2.2813647	-0.5949483	0.59494877
205383_s_at	26137	ZBTB20	Hs.655108	zinc finger and BTB domain containing 2	-2.2814596	-0.5949788	0.59497833
243102_at			Hs.655836		-2.284879	-0.59605885	0.59605885
203088_at	10516	FBLN5	Hs.723859	fibulin 5	-2.2849417	-0.5960789	0.5960784
229354_at	57491	AHRR		aryl-hydrocarbon receptor repressor	-2.2863164	-0.5965128	0.5965123
222184_at			Hs.652769		-2.2866302	-0.5966115	0.5966115
212909_at	116372	LYPD1	Hs.432395	LY6/PLAUR domain containing 1	-2.2883797	-0.5971632	0.5971632
229150_at			Hs.659093		-2.2896385	-0.5975599	0.5975599
222881_at	10855	HPSE	Hs.44227	heparanase	-2.2897096	-0.59758234	0.59758234
227868_at	154761	LOC154761	Hs.716689	hypothetical LOC154761	-2.290256	-0.5977545	0.5977545
1553994_at	4907	NT5E	Hs.153952	5'-nucleotidase, ecto (CD73)	-2.2904136	-0.59780407	0.59780407
208510_s_at	5468	PPARG	Hs.162646	peroxisome proliferator-activated recepto	-2.2905476	-0.5978465	0.59784603
205633_s_at	211	ALAS1	Hs.476308	aminolevulinate, delta-, synthase 1	-2.2919068	-0.59827423	0.59827423
232238_at	259266	ASPM	Hs.121028	asp (abnormal spindle) homolog, microce	-2.2924495	-0.59844494	0.59844494
205006_s_at	9397	NMT2	Hs.60339	N-myristoyltransferase 2	-2.2930777	-0.5986428	0.59864235
213484_at			Hs.66187		-2.293615	-0.5988116	0.5988116
242163_at	9967	THRAP3	Hs.160211	thyroid hormone receptor associated prote	-2.2939062	-0.5989032	0.5989032
221618_s_at	51616	TAF9B	Hs.592248	TAF9B RNA polymerase II, TATA box b	-2.2939956	-0.5989313	0.5989313
232797_at			Hs.656287		-2.294719	-0.59915876	0.59915876
241762_at					-2.297492	-0.60002995	0.60002995
242986_at			Hs.678572		-2.297565	-0.60005283	0.60005283
226587_at	6638	SNRPN	Hs.592473	small nuclear ribonucleoprotein polypepti	-2.3008578	-0.60108566	0.60108614
227771_at	3977	LIFR	Hs.133421	leukemia inhibitory factor receptor alpha	-2.3014715	-0.6012783	0.6012783
228825_at	22949	PTGR1	Hs.584864	prostaglandin reductase 1	-2.3017416	-0.6013627	0.6013632
205079_s_at	8777	MPDZ	Hs.169378	multiple PDZ domain protein	-2.303371	-0.6018734	0.6018734

207626_s_at	6542	SLC7A2	Hs.448520	solute carrier family 7 (cationic amino aci	-2.3043046	-0.6021657	0.6021657
225524_at	118429	ANTXR2	Hs.162963	anthrax toxin receptor 2	-2.304751	-0.6023054	0.6023054
226281_at	92737	DNER	Hs.234074	delta/notch-like EGF repeat containing	-2.3048759	-0.6023445	0.6023445
244511_at			Hs.669666		-2.3057544	-0.60261965	0.6026192
221788_at	5238	PGM3	Hs.708038	phosphoglucomutase 3	-2.3071043	-0.60304165	0.60304165
222108_at	347902	AMIGO2	Hs.121520	adhesion molecule with Ig-like domain 2	-2.3083827	-0.60344124	0.60344124
204298_s_at	4015	LOX	Hs.102267	lysyl oxidase	-2.30852	-0.60348415	0.60348415
242553_at					-2.3169377	-0.6061096	0.6061096
225112_at	10152	ABI2	Hs.471156	abl-interactor 2	-2.3181818	-0.6064968	0.6064968
230109_at	27115	PDE7B	Hs.594417	phosphodiesterase 7B	-2.3212047	-0.60743666	0.60743713
204669_s_at	11237	RNF24	Hs.589884	ring finger protein 24	-2.3234692	-0.60814047	0.60814
202833_s_at	5265	SERPINA1	Hs.525557	serpin peptidase inhibitor, clade A (alpha-	-2.3272874	-0.60932446	0.60932493
209568_s_at	23179	RGL1	Hs.497148	ral guanine nucleotide dissociation stimul	-2.3289173	-0.6098294	0.6098299
201522_x_at	6638 /// 8926	SNRPN /// SNU	Hs.555970	small nuclear ribonucleoprotein polypepti	-2.3290658	-0.6098757	0.6098757
201842_s_at	2202	EFEMP1	Hs.76224	EGF-containing fibulin-like extracellular	-2.3296661	-0.61006165	0.61006165
1559067_a_at			Hs.595538		-2.3297713	-0.6100943	0.6100941
203925_at	2730	GCLM	Hs.315562	glutamate-cysteine ligase, modifier subun	-2.33426	-0.6114826	0.6114826
225388_at	10098	TSPAN5	Hs.118118	tetraspanin 5	-2.336066	-0.6120405	0.6120405
235019_at	1368	CPM	Hs.654387	carboxypeptidase M	-2.3366406	-0.6122179	0.6122179
228054_at	93109	TMEM44	Hs.478729	transmembrane protein 44	-2.3376818	-0.6125393	0.6125393
243409_at	2300	FOXL1	Hs.533830	forkhead box L1	-2.3384058	-0.6127629	0.61276245
224964_s_at	54331	GNG2	Hs.187772	guanine nucleotide binding protein (G pro	-2.3392973	-0.6130376	0.6130376
204792_s_at	9742	IFT140	Hs.389438	intraflagellar transport 140 homolog (Chla	-2.339976	-0.6132469	0.6132469
228058_at	124220	ZG16B	Hs.105887	zymogen granule protein 16 homolog B (r	-2.3414948	-0.6137152	0.6137147
242439_s_at	171023	ASXL1	Hs.374043	additional sex combs like 1 (Drosophila)	-2.3414948	-0.6137147	0.6137152
205184_at	2786	GNG4	Hs.159711	guanine nucleotide binding protein (G pro	-2.3429914	-0.6141758	0.6141758
203139_at	1612	DAPK1	Hs.380277	death-associated protein kinase 1	-2.3451514	-0.6148405	0.6148405
225008_at	444	ASPH	Hs.332422	aspartate beta-hydroxylase	-2.3453064	-0.6148882	0.6148882
201109_s_at	7057	THBS1	Hs.164226	thrombospondin 1	-2.3473816	-0.6155262	0.6155262
202124_s_at	66008	TRAK2	Hs.152774	trafficking protein, kinesin binding 2	-2.347869	-0.6156759	0.6156759
1557961_s_at	100127983	LOC100127983	Hs.642655	hypothetical protein LOC100127983	-2.347877	-0.61567855	0.6156783
223395_at	25890	ABI3BP	Hs.477015	ABI family, member 3 (NESH) binding p	-2.3483222	-0.61581516	0.61581516
231644_at			Hs.200266		-2.3496637	-0.61622715	0.61622715
204041_at	4129	MAOB	Hs.654473	monoamine oxidase B	-2.3512492	-0.616714	0.6167135
215037_s_at	598	BCL2L1	Hs.516966	BCL2-like 1	-2.3516433	-0.61683464	0.61683464
213195_at	201229	C17orf108	Hs.724502	chromosome 17 open reading frame 108	-2.3518486	-0.6168976	0.6168976
202460_s_at	9663	LPIN2	Hs.132342	lipin 2	-2.3530054	-0.61725235	0.61725235
225381_at	399959	LOC399959	Hs.44098	hypothetical LOC399959	-2.3550875	-0.61789036	0.61789036
1558733_at	253461	ZBTB38	Hs.518301	zinc finger and BTB domain containing 3	-2.3560934	-0.6181984	0.6181984
229679_at	100510175 /// 40	C12orf76 /// LOC	Hs.44817	chromosome 12 open reading frame 76 ///	-2.356874	-0.6184373	0.6184373
226591_at	6638	SNRPN	Hs.592473	small nuclear ribonucleoprotein polypepti	-2.357354	-0.61858416	0.61858416
214845_s_at	813	CALU	Hs.724446	calumenin	-2.3585236	-0.6189418	0.61894226
222835_at	79875	THSD4	Hs.387057	thrombospondin, type I, domain containin	-2.3587239	-0.6190033	0.6190033

235570_at	27303	RBMS3	Hs.696468	RNA binding motif, single stranded intera	-2.3589017	-0.61905766	0.61905766
237664_at			Hs.671789		-2.3592527	-0.61916494	0.61916494
221511_x_at	9236	CCPG1	Hs.285051	cell cycle progression 1	-2.3602197	-0.6194606	0.6194606
201826_s_at	51097	SCCPDH	Hs.498397	saccharopine dehydrogenase (putative)	-2.3615477	-0.6198664	0.6198664
205462_s_at	3241	HPCAL1	Hs.580427	hippocalcin-like 1	-2.3633187	-0.6204071	0.6204071
200919_at	1912	PHC2	Hs.524271	polyhomeotic homolog 2 (Drosophila)	-2.3657446	-0.62114716	0.62114716
233375_at	84288	EFCAB2	Hs.134857	EF-hand calcium binding domain 2	-2.3681064	-0.6218667	0.6218672
212143_s_at	3486	IGFBP3	Hs.450230	insulin-like growth factor binding protein	-2.3682027	-0.62189627	0.62189627
201340_s_at	8507	ENC1	Hs.104925	ectodermal-neural cortex 1 (with BTB-lik	-2.3684554	-0.62197304	0.6219735
1564475_s_at	728723	LOC728723	Hs.161338	hypothetical LOC728723	-2.3685894	-0.62201405	0.62201405
215575_at	9659	PDE4DIP		phosphodiesterase 4D interacting protein	-2.3706741	-0.6226487	0.6226487
226633_at	51762	RAB8B	Hs.389733	RAB8B, member RAS oncogene family	-2.3739095	-0.62363243	0.62363243
1569500_at			Hs.638926		-2.3752673	-0.6240449	0.6240449
209014_at	9500	MAGED1	Hs.5258	melanoma antigen family D, 1	-2.3763618	-0.62437725	0.62437725
228579_at			Hs.587442		-2.3785958	-0.6250553	0.62505484
243008_at			Hs.671383		-2.3786147	-0.62506056	0.62506104
1558599_at			Hs.662681		-2.3794396	-0.6253109	0.6253109
210139_s_at	5376	PMP22	Hs.372031	peripheral myelin protein 22	-2.380485	-0.625628	0.6256275
233490_at	51164	DCTN4	Hs.675564	dynactin 4 (p62)	-2.3824172	-0.6262131	0.6262131
202275_at	2539	G6PD	Hs.461047	glucose-6-phosphate dehydrogenase	-2.382685	-0.62629414	0.62629414
1556047_s_at	57692	MAGEE1	Hs.8453	melanoma antigen family E, 1	-2.3833578	-0.62649775	0.62649775
242624_at	84448	ABLIM2	Hs.233404	actin binding LIM protein family, membe	-2.3844624	-0.626832	0.626832
223551_at	5570	PKIB	Hs.724566	protein kinase (cAMP-dependent, catalyti	-2.3872287	-0.6276684	0.6276684
219230_at	55273	TMEM100	Hs.173233	transmembrane protein 100	-2.3888767	-0.6281662	0.6281662
1555978_s_at			Hs.713646		-2.389769	-0.6284356	0.6284356
231579_s_at	7077	TIMP2	Hs.633514	TIMP metallopeptidase inhibitor 2	-2.3907013	-0.6287174	0.62871647
1559410_at			Hs.490920		-2.3922536	-0.6291852	0.6291852
230954_at	140688	C20orf112	Hs.516978	chromosome 20 open reading frame 112	-2.3931127	-0.6294441	0.6294441
210665_at	7035	TFPI	Hs.516578	tissue factor pathway inhibitor (lipoprotei	-2.3937714	-0.62964296	0.6296425
202724_s_at	2308	FOXO1	Hs.370666	forkhead box O1	-2.3944767	-0.62985516	0.62985516
1559957_a_at	642852	LOC642852	Hs.11637	hypothetical LOC642852	-2.3949008	-0.62998295	0.62998295
216278_at			Hs.655490		-2.3952756	-0.6300957	0.63009596
1555471_a_at	56776	FMN2	Hs.24889	formin 2	-2.3955154	-0.6301682	0.63016796
200962_at	6160	RPL31	Hs.469473	ribosomal protein L31	-2.3960092	-0.63031673	0.63031673
212040_at	10618	TGOLN2	Hs.593382	trans-golgi network protein 2	-2.397466	-0.63075495	0.6307554
200755_s_at	813	CALU	Hs.724446	calumenin	-2.3977711	-0.630847	0.630847
227351_at	730094	C16orf52	Hs.498890	chromosome 16 open reading frame 52	-2.398181	-0.6309705	0.63097
228909_at	642852	LOC642852	Hs.11637	hypothetical LOC642852	-2.401271	-0.63189936	0.6318989
236656_s_at	100288911	LOC100288911	Hs.432924	hypothetical LOC100288911	-2.4015877	-0.63199425	0.63199425
203810_at	11080	DNAJB4	Hs.13852	DnaJ (Hsp40) homolog, subfamily B, mer	-2.4029152	-0.6323929	0.6323929
211177_s_at	10587	TXNRD2	Hs.443430	thioredoxin reductase 2	-2.4034514	-0.6325536	0.63255405
226886_at	2673	GFPT1	Hs.580300	glutamine--fructose-6-phosphate transami	-2.4038718	-0.63267994	0.63267994
239942_at			Hs.547771		-2.4043734	-0.6328304	0.6328306

243785_at	100272217	LOC100272217	Hs.601255	hypothetical LOC100272217	-2.4122574	-0.6351919	0.6351919
210718_s_at	100294341 /// 51	ARL17A /// LOC100294341		ADP-ribosylation factor-like 17A /// ADP	-2.4128315	-0.6353636	0.6353636
206623_at	5145	PDE6A	Hs.567314	phosphodiesterase 6A, cGMP-specific, ro	-2.4129272	-0.6353922	0.6353922
204906_at	6196	RPS6KA2	Hs.655277	ribosomal protein S6 kinase, 90kDa, poly	-2.4138684	-0.6356735	0.6356735
233986_s_at	64857	PLEKHG2	Hs.631574	pleckstrin homology domain containing, f	-2.4141612	-0.63576126	0.6357608
214378_at	7035	TFPI	Hs.516578	tissue factor pathway inhibitor (lipoprotei	-2.4156203	-0.6361971	0.6361966
1555366_at	100131275	NSAP11	Hs.623961	nervous system abundant protein 11	-2.4156978	-0.63622	0.63622
1568643_a_at			Hs.535775		-2.4178033	-0.63684845	0.63684845
205992_s_at	3600	IL15	Hs.654378	interleukin 15	-2.4196389	-0.63739586	0.63739586
228573_at	118429	ANTXR2	Hs.162963	anthrax toxin receptor 2	-2.4204204	-0.63762903	0.63762856
204341_at	10626	TRIM16	Hs.123534	tripartite motif-containing 16	-2.4204514	-0.6376381	0.6376381
226931_at	83857	TMTC1	Hs.401954	transmembrane and tetratricopeptide repe	-2.4235222	-0.63855267	0.63855267
217591_at			Hs.721360		-2.4237497	-0.6386204	0.6386204
203408_s_at	6304	SATB1	Hs.517717	SATB homeobox 1	-2.4248219	-0.6389394	0.6389394
232279_at	23338	PHF15	Hs.483419	PHD finger protein 15	-2.4276237	-0.6397724	0.6397724
243629_x_at	100507057	LOC100507057		hypothetical LOC100507057	-2.4286077	-0.6400647	0.6400647
201719_s_at	2037	EPB41L2	Hs.486470	erythrocyte membrane protein band 4.1-lil	-2.4293253	-0.64027786	0.64027786
1553122_s_at	57786	RBAK	Hs.396178	RB-associated KRAB zinc finger	-2.4294634	-0.6403189	0.6403189
213627_at	10916	MAGED2	Hs.522665	melanoma antigen family D, 2	-2.430469	-0.6406174	0.6406174
238441_at	5563	PRKAA2	Hs.437039	protein kinase, AMP-activated, alpha 2 ca	-2.4321501	-0.64111614	0.64111614
235308_at	26137	ZBTB20	Hs.655108	zinc finger and BTB domain containing 2	-2.4354868	-0.6421051	0.6421051
229302_at	130733	TMEM178	Hs.40808	transmembrane protein 178	-2.43855	-0.64301205	0.64301157
217513_at	284021	C17orf60	Hs.631749	chromosome 17 open reading frame 60	-2.4416792	-0.6439371	0.64393663
1559663_at			Hs.676460		-2.4441621	-0.64467	0.64467
236155_at	79670	ZCCHC6	Hs.597057	Zinc finger, CCHC domain containing 6	-2.444836	-0.64486885	0.64486885
228908_s_at	642852	LOC642852	Hs.11637	hypothetical LOC642852	-2.4481733	-0.64585304	0.64585257
227290_at			Hs.60257		-2.4496891	-0.64629936	0.64629936
229103_at	7473	WNT3	Hs.445884	wingless-type MMTV integration site fam	-2.4501605	-0.6464381	0.6464381
213222_at	23236	PLCB1	Hs.431173	phospholipase C, beta 1 (phosphoinositide	-2.451412	-0.6468067	0.64680624
1567224_at	8091	HMGA2	Hs.505924	high mobility group AT-hook 2	-2.4517028	-0.6468921	0.6468921
214954_at	26032	SUSD5	Hs.196647	sushi domain containing 5	-2.453628	-0.64745855	0.6474581
201242_s_at	481	ATP1B1	Hs.291196	ATPase, Na+/K+ transporting, beta 1 poly	-2.4544158	-0.6476898	0.6476898
232176_at	84189	SLITRK6	Hs.525105	SLIT and NTRK-like family, member 6	-2.4582527	-0.6488166	0.6488166
209693_at	23245	ASTN2	Hs.601562	astrotactin 2	-2.4588296	-0.64898586	0.64898586
205364_at	8309	ACOX2	Hs.444959	acyl-CoA oxidase 2, branched chain	-2.4616048	-0.6497998	0.64979935
239503_at			Hs.554052		-2.4624047	-0.65003395	0.65003395
226051_at	140606	SELM	Hs.55940	selenoprotein M	-2.4652157	-0.650857	0.650857
233364_s_at			Hs.715755		-2.4707038	-0.65246105	0.65246105
209676_at	7035	TFPI	Hs.516578	tissue factor pathway inhibitor (lipoprotei	-2.4742122	-0.6534848	0.65348434
236439_at			Hs.660734		-2.4744828	-0.6535635	0.6535635
231956_at	57674	RNF213	Hs.195642	ring finger protein 213	-2.4746742	-0.6536193	0.6536193
204774_at	2123	EVI2A	Hs.591198	ecotropic viral integration site 2A	-2.4754071	-0.6538329	0.6538329
212607_at	10000	AKT3	Hs.498292	v-akt murine thymoma viral oncogene hor	-2.4774582	-0.6544304	0.6544304

226973_at	128434	VSTM2L	Hs.517029	V-set and transmembrane domain contain	-2.4784067	-0.6547065	0.6547065
202478_at	28951	TRIB2	Hs.467751	tribbles homolog 2 (Drosophila)	-2.4784124	-0.6547084	0.6547079
227992_s_at	147650	NCRNA00085	Hs.467174	non-protein coding RNA 85	-2.4815361	-0.65561676	0.65561676
233413_at			Hs.210390		-2.4823434	-0.65585136	0.65585136
232182_at	286272	LOC286272	Hs.648390	hypothetical protein LOC286272	-2.4836626	-0.6562345	0.65623474
233540_s_at	55755	CDK5RAP2	Hs.269560	CDK5 regulatory subunit associated prote	-2.4846747	-0.6565285	0.6565285
200757_s_at	813	CALU	Hs.724446	calumenin	-2.4856405	-0.65680885	0.65680885
224209_s_at	9615	GDA	Hs.494163	guanine deaminase	-2.4877996	-0.65743494	0.6574354
201426_s_at	7431	VIM	Hs.455493	vimentin	-2.4892004	-0.6578417	0.6578407
224480_s_at	84803	AGPAT9	Hs.99196	1-acylglycerol-3-phosphate O-acyltransfe	-2.492419	-0.6587732	0.6587734
214643_x_at	274	BIN1	Hs.193163	bridging integrator 1	-2.4939241	-0.6592088	0.6592088
227032_at	5362	PLXNA2	Hs.497626	plexin A2	-2.4943643	-0.6593361	0.6593361
210299_s_at	2273	FHL1	Hs.435369	four and a half LIM domains 1	-2.4998808	-0.6609297	0.6609297
242778_at	9404	LPXN	Hs.125474	leupaxin	-2.5014777	-0.6613903	0.6613903
236565_s_at	55323	LARP6	Hs.416755	La ribonucleoprotein domain family, mem	-2.502298	-0.6616268	0.6616268
220169_at	80008	TMEM156	Hs.374147	transmembrane protein 156	-2.5026817	-0.66173744	0.66173744
1565701_at			Hs.520751		-2.5032823	-0.66191053	0.66191053
202368_s_at	9697	TRAM2	Hs.520182	translocation associated membrane protei	-2.5064127	-0.66281223	0.66281176
222996_s_at	51523	CXXC5	Hs.189119	CXXC finger protein 5	-2.509998	-0.66384315	0.66384315
235645_at	114799	ESCO1	Hs.464733	establishment of cohesion 1 homolog 1 (S	-2.5112395	-0.6641998	0.6641998
208682_s_at	10916	MAGED2	Hs.522665	melanoma antigen family D, 2	-2.511359	-0.66423416	0.66423416
235555_at			Hs.48729		-2.5114007	-0.6642461	0.6642461
241871_at	814	CAMK4	Hs.591269	calcium/calmodulin-dependent protein ki	-2.5175335	-0.6660054	0.6660056
241459_at			Hs.720426		-2.517767	-0.66607237	0.66607237
231890_at			Hs.273830		-2.5216994	-0.6671982	0.6671982
226252_at			Hs.202577		-2.5220096	-0.6672869	0.6672869
225227_at			Hs.536655		-2.526251	-0.668499	0.668499
235318_at	2200	FBN1	Hs.591133	fibrillin 1	-2.5268557	-0.6686716	0.6686716
211981_at	1282	COL4A1	Hs.17441	collagen, type IV, alpha 1	-2.5341203	-0.6707425	0.6707425
234224_at			Hs.675501		-2.5356386	-0.6711745	0.6711745
1553995_a_at	4907	NT5E	Hs.153952	5'-nucleotidase, ecto (CD73)	-2.5378091	-0.67179155	0.67179203
219032_x_at	23596	OPN3	Hs.724579	opsin 3	-2.5400186	-0.67241955	0.67241955
242345_at	340267	COL28A1	Hs.491104	collagen, type XXVIII, alpha 1	-2.54128	-0.67277765	0.67277765
208964_s_at	3992	FADS1	Hs.503546	fatty acid desaturase 1	-2.5419133	-0.6729574	0.6729574
224392_s_at	23596	OPN3	Hs.724579	opsin 3	-2.5448117	-0.6737795	0.6737795
1562955_at			Hs.683429		-2.5477474	-0.6746111	0.6746111
238121_at	256356	GK5	Hs.135904	glycerol kinase 5 (putative)	-2.5489838	-0.6749611	0.6749611
1555867_at	2786	GNG4	Hs.159711	guanine nucleotide binding protein (G pro	-2.5552697	-0.6767378	0.6767378
1566482_at			Hs.684006		-2.5582037	-0.6775656	0.6775656
232881_at	149775	GNAS-AS	Hs.122718	GNAS antisense RNA (non-protein codin	-2.5602043	-0.6781297	0.6781292
229908_s_at	64718	UNKL	Hs.643536	unkempt homolog (Drosophila)-like	-2.5651839	-0.6795311	0.6795311
1555778_a_at	10631	POSTN	Hs.136348	periostin, osteoblast specific factor	-2.5690444	-0.6806159	0.6806159
228393_s_at	55900	ZNF302	Hs.436350	zinc finger protein 302	-2.5693552	-0.68070316	0.68070316

202557_at	6782	HSPA13	Hs.352341	heat shock protein 70kDa family, member	-2.5698137	-0.6808319	0.6808319
214087_s_at	4604	MYBPC1	Hs.654589	myosin binding protein C, slow type	-2.5707245	-0.6810875	0.6810875
218983_at	51279	C1RL	Hs.631730	complement component 1, r subcomponer	-2.571598	-0.6813326	0.6813326
218718_at	56034	PDGFC	Hs.570855	platelet derived growth factor C	-2.572722	-0.68164825	0.6816473
1556633_at	284677	C1orf204		chromosome 1 open reading frame 204	-2.5734415	-0.6818495	0.6818495
239778_x_at			Hs.690847		-2.5736678	-0.6819129	0.6819129
221207_s_at	26960	NBEA	Hs.491172	neurobeachin	-2.574002	-0.68200636	0.68200684
225202_at	22836	RHOBTB3	Hs.445030	Rho-related BTB domain containing 3	-2.5743942	-0.6821165	0.6821165
230934_at	100506057 /// 28	LOC100506057	Hs.469002	hypothetical LOC100506057 /// serine/thr	-2.5805244	-0.68383217	0.68383217
1570629_at			Hs.684411		-2.582101	-0.68427277	0.68427277
217540_at	91775	FAM55C	Hs.595933	family with sequence similarity 55, memb	-2.5831997	-0.6845794	0.68457985
229549_at			Hs.592258		-2.5875998	-0.6858072	0.6858072
233112_at			Hs.675456		-2.5901659	-0.686522	0.6865225
201289_at	3491	CYR61	Hs.8867	cysteine-rich, angiogenic inducer, 61	-2.5940888	-0.6876135	0.68761444
220148_at	64577	ALDH8A1	Hs.486520	aldehyde dehydrogenase 8 family, membe	-2.5947938	-0.68780994	0.68780994
217388_s_at	8942	KYNU	Hs.470126	kynureninase (L-kynurenine hydrolase)	-2.5949173	-0.6878443	0.6878443
226358_at	83464	APH1B	Hs.511703	anterior pharynx defective 1 homolog B ((	-2.59974	-0.6891837	0.6891837
224516_s_at	51523	CXXC5	Hs.189119	CXXC finger protein 5	-2.6029782	-0.6900816	0.6900816
203845_at	8850	KAT2B	Hs.533055	K(lysine) acetyltransferase 2B	-2.6069765	-0.6911888	0.6911888
224663_s_at	1073	CFL2	Hs.180141	cofilin 2 (muscle)	-2.6071627	-0.6912403	0.6912403
221675_s_at	56994	CHPT1	Hs.293077	choline phosphotransferase 1	-2.6097112	-0.6919451	0.6919451
239001_at	4257	MGST1	Hs.389700	Microsomal glutathione S-transferase 1	-2.6127284	-0.6927786	0.6927786
235844_at	10745	PHTF1	Hs.655824	putative homeodomain transcription facto	-2.6149201	-0.6933837	0.6933832
216069_at			Hs.661229		-2.614962	-0.6933949	0.69339514
229086_at	148898	C1orf213	Hs.61884	chromosome 1 open reading frame 213	-2.615858	-0.69364214	0.69364214
201471_s_at	8878	SQSTM1	Hs.437277	sequestosome 1	-2.62006	-0.6947994	0.6948004
201540_at	2273	FHL1	Hs.435369	four and a half LIM domains 1	-2.6208065	-0.6950054	0.6950054
202976_s_at	22836	RHOBTB3	Hs.445030	Rho-related BTB domain containing 3	-2.621569	-0.6952152	0.6952152
232748_at	5069	PAPPA		pregnancy-associated plasma protein A, p	-2.6218255	-0.6952858	0.6952858
225098_at	10152	ABI2	Hs.471156	abl-interactor 2	-2.6235244	-0.6957531	0.6957531
218211_s_at	79083	MLPH	Hs.102406	melanophilin	-2.624482	-0.6960163	0.6960163
201869_s_at	6907	TBL1X	Hs.495656	transducin (beta)-like 1X-linked	-2.625084	-0.6961818	0.6961818
213093_at	5578	PRKCA	Hs.531704	protein kinase C, alpha	-2.6251934	-0.6962118	0.6962118
223006_s_at	23731	C9orf5	Hs.308074	chromosome 9 open reading frame 5	-2.6258943	-0.69640446	0.69640446
212314_at	23231	SEL1L3	Hs.479384	sel-1 suppressor of lin-12-like 3 (C. elega	-2.6274154	-0.69682217	0.69682217
223007_s_at	23731	C9orf5	Hs.308074	chromosome 9 open reading frame 5	-2.6285446	-0.6971321	0.6971321
244425_at					-2.6288626	-0.6972194	0.6972194
241745_at	100507557	LOC100507557		hypothetical LOC100507557	-2.6357915	-0.69911814	0.69911814
1556250_at			Hs.656807		-2.6376147	-0.6996169	0.6996169
201467_s_at	1728	NQO1	Hs.406515	NAD(P)H dehydrogenase, quinone 1	-2.6395001	-0.70013237	0.70013237
212614_at	84159	ARID5B	Hs.535297	AT rich interactive domain 5B (MRF1-lik	-2.6400654	-0.70028687	0.70028687
209894_at	3953	LEPR	Hs.723178	leptin receptor	-2.6428976	-0.7010603	0.7010603
213112_s_at	8878	SQSTM1	Hs.437277	sequestosome 1	-2.643338	-0.70118046	0.70118046

230585_at			Hs.129583		-2.6434917	-0.7012224	0.7012224
227758_at	85004	RERG	Hs.199487	RAS-like, estrogen-regulated, growth inhi	-2.6434987	-0.7012243	0.7012243
223569_at	84513	PPAPDC1B	Hs.567619	phosphatidic acid phosphatase type 2 dom	-2.6437573	-0.7012949	0.7012949
201666_at	7076	TIMP1	Hs.522632	TIMP metallopeptidase inhibitor 1	-2.6447346	-0.7015619	0.701561
241484_x_at					-2.6454008	-0.7017431	0.7017431
213326_at	6843	VAMP1	Hs.20021	vesicle-associated membrane protein 1 (sy	-2.6478438	-0.70240927	0.7024088
214930_at	26050	SLITRK5	Hs.591208	SLIT and NTRK-like family, member 5	-2.6481512	-0.7024927	0.7024927
203085_s_at	7040	TGFB1	Hs.645227	transforming growth factor, beta 1	-2.648812	-0.7026725	0.70267296
238933_at					-2.6491132	-0.702755	0.7027545
209305_s_at	4616	GADD45B	Hs.110571	growth arrest and DNA-damage-inducible	-2.6506555	-0.7031746	0.7031746
225946_at	11228	RASSF8	Hs.696433	Ras association (RalGDS/AF-6) domain f	-2.6508412	-0.70322514	0.70322514
223008_s_at	23731	C9orf5	Hs.308074	chromosome 9 open reading frame 5	-2.6556926	-0.70454407	0.70454407
206765_at	3759	KCNJ2	Hs.1547	potassium inwardly-rectifying channel, su	-2.6570024	-0.7048998	0.7048998
1557285_at	727738	AREGB	Hs.645475	Amphiregulin B	-2.6573362	-0.7049904	0.7049904
232113_at			Hs.715755		-2.6578913	-0.70514107	0.70514107
216493_s_at	10643	IGF2BP3	Hs.700696	insulin-like growth factor 2 mRNA bindir	-2.6582005	-0.705225	0.705225
241936_x_at			Hs.117688		-2.6590204	-0.7054472	0.7054477
205627_at	978	CDA	Hs.466910	cytidine deaminase	-2.660573	-0.7058687	0.70586824
243841_at	23224	SYNE2	Hs.525392	spectrin repeat containing, nuclear envelo	-2.6606054	-0.7058773	0.7058773
210102_at	4013	VWA5A	Hs.152944	von Willebrand factor A domain containir	-2.666533	-0.7074828	0.70748234
219569_s_at	80723	TMEM22	Hs.477692	transmembrane protein 22	-2.6672056	-0.7076645	0.7076645
210202_s_at	274	BIN1	Hs.193163	bridging integrator 1	-2.6689878	-0.7081466	0.7081461
1566622_at			Hs.684040		-2.6690786	-0.7081709	0.7081709
236699_at			Hs.724721		-2.6695454	-0.7082968	0.70829725
201848_s_at	664	BNIP3	Hs.144873	BCL2/adenovirus E1B 19kDa interacting	-2.6731927	-0.7092819	0.7092819
1555330_at	2729	GCLC	Hs.654465	glutamate-cysteine ligase, catalytic subun	-2.6735082	-0.7093668	0.7093673
239696_at			Hs.666516		-2.6746225	-0.7096677	0.7096677
234986_at	2730	GCLM	Hs.315562	glutamate-cysteine ligase, modifier subun	-2.674713	-0.709692	0.709692
213258_at	7035	TFPI	Hs.516578	tissue factor pathway inhibitor (lipoprotei	-2.676064	-0.7100563	0.7100563
201124_at	3693	ITGB5	Hs.536663	integrin, beta 5	-2.6778786	-0.71054506	0.71054554
1557289_s_at	84163	GTF2IRD2	Hs.647017	GTF2I repeat domain containing 2	-2.682299	-0.7117348	0.71173525
224560_at	7077	TIMP2	Hs.633514	TIMP metallopeptidase inhibitor 2	-2.687395	-0.71310425	0.71310425
203811_s_at	11080	DNAJB4	Hs.13852	DnaJ (Hsp40) homolog, subfamily B, mer	-2.6922183	-0.7143979	0.71439743
227963_at			Hs.659940		-2.6929047	-0.7145817	0.7145815
242037_at					-2.6931136	-0.7146373	0.71463776
206547_s_at	5475	PPEF1	Hs.211589	protein phosphatase, EF-hand calcium bir	-2.694806	-0.71509075	0.71509075
217904_s_at	23621	BACE1	Hs.504003	beta-site APP-cleaving enzyme 1	-2.695877	-0.71537733	0.71537733
244358_at					-2.6959116	-0.71538687	0.7153864
243634_at					-2.7019036	-0.7169881	0.7169881
1555855_at			Hs.677669		-2.706249	-0.7181473	0.7181473
231897_at	22949	PTGR1	Hs.584864	prostaglandin reductase 1	-2.7064493	-0.7182007	0.7182007
203438_at	8614	STC2	Hs.233160	stanniocalcin 2	-2.7125032	-0.7198124	0.7198124
222253_s_at	29774	POM121L9P	Hs.534980	POM121 membrane glycoprotein-like 9, p	-2.7127578	-0.7198801	0.7198801

231380_at	116328	C8orf34	Hs.491941	chromosome 8 open reading frame 34	-2.712787	-0.719888	0.71988773
202609_at	2059	EPS8	Hs.591160	epidermal growth factor receptor pathway	-2.715431	-0.7205906	0.7205906
236140_at	2730	GCLM	Hs.315562	glutamate-cysteine ligase, modifier subun	-2.7160342	-0.7207508	0.7207508
202619_s_at	5352	PLOD2	Hs.477866	procollagen-lysine, 2-oxoglutarate 5-dioxy	-2.7163322	-0.72082996	0.72082996
202017_at	2052	EPHX1	Hs.89649	epoxide hydrolase 1, microsomal (xenobic	-2.71686	-0.72097015	0.72097015
205623_at	218	ALDH3A1	Hs.531682	aldehyde dehydrogenase 3 family, membe	-2.717108	-0.72103596	0.72103596
224352_s_at	1073	CFL2	Hs.180141	cofilin 2 (muscle)	-2.7188869	-0.721508	0.721508
201389_at	3678	ITGA5	Hs.505654	integrin, alpha 5 (fibronectin receptor, alp	-2.7220535	-0.7223482	0.72234726
228824_s_at	22949	PTGR1	Hs.584864	prostaglandin reductase 1	-2.7250836	-0.72315025	0.72315025
227082_at			Hs.193784		-2.728678	-0.72410107	0.72410107
212822_at	57493	HEG1	Hs.477420	HEG homolog 1 (zebrafish)	-2.7291813	-0.7242341	0.7242341
241384_x_at	256356	GK5	Hs.135904	glycerol kinase 5 (putative)	-2.7302585	-0.7245188	0.7245188
222784_at	64093	SMOC1	Hs.497349	SPARC related modular calcium binding	-2.7311113	-0.7247443	0.72474384
1562056_at					-2.7324297	-0.7250924	0.72509193
204589_at	9891	NUAK1	Hs.724500	NUAK family, SNF1-like kinase, 1	-2.7386727	-0.72673845	0.72673845
235476_at	286827	TRIM59	Hs.212957	tripartite motif-containing 59	-2.7395492	-0.72696924	0.72696924
224279_s_at	26256	CABYR	Hs.511983	calcium binding tyrosine-(Y)-phosphoryla	-2.7402492	-0.7271538	0.7271533
242470_at	126272	EID2B	Hs.135181	EP300 interacting inhibitor of differentiat	-2.7415273	-0.72748995	0.72748995
240038_at			Hs.608694		-2.7418528	-0.7275758	0.7275753
235976_at	84189	SLITRK6	Hs.525105	SLIT and NTRK-like family, member 6	-2.7424881	-0.7277427	0.7277427
232405_at			Hs.662234		-2.7435324	-0.72801733	0.72801733
225387_at	10098	TSPAN5	Hs.118118	tetraspanin 5	-2.745265	-0.7284727	0.7284727
207415_at	22925	PLA2R1	Hs.410477	phospholipase A2 receptor 1, 180kDa	-2.75752	-0.73168564	0.73168564
244414_at					-2.7575855	-0.7317028	0.7317028
201627_s_at	3638	INSIG1	Hs.520819	insulin induced gene 1	-2.7582564	-0.7318783	0.7318783
212816_s_at	875	CBS	Hs.533013	cystathionine-beta-synthase	-2.7583475	-0.7319021	0.7319021
203343_at	7358	UGDH	Hs.572518	UDP-glucose 6-dehydrogenase	-2.7597847	-0.73227787	0.73227787
234222_at			Hs.677309		-2.7598975	-0.7323072	0.73230743
227443_at	286343	C9orf150	Hs.445356	chromosome 9 open reading frame 150	-2.759928	-0.73231554	0.73231506
241376_at	100130097	LOC100130097	Hs.298987	Hypothetical LOC100130097	-2.7607198	-0.732522	0.7325225
222450_at	56937	PMEPA1	Hs.517155	prostate transmembrane protein, androgen	-2.7691338	-0.73471737	0.73471737
220936_s_at	55766	H2AFJ	Hs.724631	H2A histone family, member J	-2.7704237	-0.73505354	0.73505306
236338_at					-2.7801018	-0.73756886	0.73756886
207442_at	1440	CSF3	Hs.2233	colony stimulating factor 3 (granulocyte)	-2.7843099	-0.73865986	0.73865986
209466_x_at	5764	PTN	Hs.371249	pleiotrophin	-2.7852228	-0.73889637	0.73889637
223915_at	54880	BCOR	Hs.659681	BCL6 corepressor	-2.786709	-0.7392812	0.7392812
239280_at			Hs.145520		-2.7914112	-0.7404976	0.7404971
207808_s_at	5627	PROS1	Hs.64016	protein S (alpha)	-2.7916079	-0.74054813	0.74054813
233723_at			Hs.663298		-2.7953527	-0.74151516	0.74151516
214577_at	4131	MAP1B	Hs.335079	microtubule-associated protein 1B	-2.8048944	-0.74397326	0.74397326
1569577_x_at			Hs.364739		-2.8089988	-0.745028	0.745028
225575_at	3977	LIFR	Hs.133421	leukemia inhibitory factor receptor alpha	-2.810966	-0.745533	0.745533
1558404_at	644242	LOC644242	Hs.515383	hypothetical LOC644242	-2.814076	-0.7463305	0.74633074

233607_at			Hs.677063		-2.8147898	-0.74651384	0.74651337
211004_s_at	221	ALDH3B1	Hs.523841	aldehyde dehydrogenase 3 family, membe	-2.8153882	-0.7466669	0.7466669
201141_at	10457	GPNMB	Hs.190495	glycoprotein (transmembrane) nmb	-2.8183832	-0.74743366	0.74743414
236769_at	158402	LOC158402	Hs.494822	hypothetical protein LOC158402	-2.8202395	-0.7479086	0.74790907
1561181_at			Hs.660277		-2.82764	-0.74979925	0.74979925
232459_at			Hs.660657		-2.8307726	-0.75059795	0.75059795
202723_s_at	2308	FOXO1	Hs.370666	forkhead box O1	-2.8310084	-0.75065804	0.75065804
1559360_at			Hs.665919		-2.8316336	-0.7508173	0.7508173
201843_s_at	2202	EFEMP1	Hs.76224	EGF-containing fibulin-like extracellular	-2.831787	-0.7508564	0.7508564
207469_s_at	8544	PIR	Hs.495728	pirin (iron-binding nuclear protein)	-2.8340268	-0.7514267	0.7514267
1555326_a_at	8754	ADAM9	Hs.591852	ADAM metallopeptidase domain 9	-2.8364859	-0.7520523	0.7520523
204099_at	6604	SMARCD3	Hs.647067	SWI/SNF related, matrix associated, actin	-2.8381183	-0.75246716	0.75246763
204072_s_at	10129	FRY	Hs.507669	furry homolog (Drosophila)	-2.8395624	-0.7528343	0.7528343
205582_s_at	2687	GGT5	Hs.437156	gamma-glutamyltransferase 5	-2.8431199	-0.75373745	0.75373745
244804_at	8878	SQSTM1	Hs.437277	sequestosome 1	-2.8487184	-0.7551565	0.7551565
233496_s_at	1073	CFL2	Hs.180141	cofilin 2 (muscle)	-2.8546376	-0.7566538	0.7566538
230682_x_at	8714	ABCC3	Hs.463421	ATP-binding cassette, sub-family C (CFT	-2.855064	-0.75676155	0.75676155
1556696_s_at	441094	FLJ42709	Hs.457407	hypothetical LOC441094	-2.857277	-0.7573204	0.7573204
239921_at	340267	COL28A1	Hs.491104	collagen, type XXVIII, alpha 1	-2.8632705	-0.758832	0.758832
1554140_at	79819	WDR78	Hs.49421	WD repeat domain 78	-2.8632715	-0.7588322	0.7588322
233126_s_at	55301	OLAH	Hs.24309	oleoyl-ACP hydrolase	-2.8632855	-0.7588358	0.7588358
1553708_at	84847	MGC16075	Hs.488236	hypothetical protein MGC16075	-2.8651903	-0.7593155	0.7593155
201313_at	2026	ENO2	Hs.511915	enolase 2 (gamma, neuronal)	-2.8675048	-0.7598977	0.7598982
238497_at	219902	TMEM136	Hs.643516	transmembrane protein 136	-2.8676233	-0.75992775	0.75992775
233955_x_at	51523	CXXC5	Hs.189119	CXXC finger protein 5	-2.8678963	-0.7599964	0.7599964
225571_at	3977	LIFR	Hs.133421	leukemia inhibitory factor receptor alpha	-2.868723	-0.7602043	0.7602043
235251_at			Hs.444290		-2.8697927	-0.76047325	0.76047325
231116_at			Hs.632463		-2.8711483	-0.7608137	0.7608142
222162_s_at	9510	ADAMTS1	Hs.643357	ADAM metallopeptidase with thrombospo	-2.872858	-0.76124334	0.76124334
221565_s_at	51063	CALHM2	Hs.241545	calcium homeostasis modulator 2	-2.8777065	-0.76245975	0.76245975
212758_s_at	6935	ZEB1	Hs.124503	zinc finger E-box binding homeobox 1	-2.8783648	-0.76262474	0.76262474
204867_at	2644	GCHFR	Hs.631717	GTP cyclohydrolase I feedback regulator	-2.8810146	-0.7632885	0.7632885
202363_at	6695	SPOCK1	Hs.596136	sparc/osteonectin, cwcv and kazal-like do	-2.8817623	-0.7634759	0.7634754
222156_x_at	9236	CCPG1	Hs.285051	cell cycle progression 1	-2.8903463	-0.7656212	0.7656212
239901_at			Hs.677739		-2.8909836	-0.76578045	0.76578
214453_s_at	10561	IFI44	Hs.82316	interferon-induced protein 44	-2.8923752	-0.7661271	0.7661276
241824_at			Hs.676352		-2.9001653	-0.76806784	0.76806736
218656_s_at	10186	LHFP	Hs.507798	lipoma HMGIC fusion partner	-2.9048269	-0.7692261	0.7692261
239153_at	100124700	HOTAIR	Hs.197076	hox transcript antisense RNA (non-protein	-2.9064124	-0.76961946	0.76961994
241970_at			Hs.682753		-2.907208	-0.76981735	0.7698169
1554485_s_at	140738	TMEM37	Hs.26216	transmembrane protein 37	-2.9101315	-0.77054214	0.77054214
201825_s_at	51097	SCCPDH	Hs.498397	saccharopine dehydrogenase (putative)	-2.9265018	-0.7745886	0.7745886
226697_at	92689	FAM114A1	Hs.476517	family with sequence similarity 114, mem	-2.928197	-0.7750063	0.7750063

1558706_a_at	84913	ATOH8	Hs.135569	Atonal homolog 8 (Drosophila)	-2.9313674	-0.7757869	0.7757869
202375_at	9871	SEC24D	Hs.189641	SEC24 family, member D (S. cerevisiae)	-2.9359536	-0.7769146	0.7769146
203675_at	4925	NUCB2	Hs.654599	nucleobindin 2	-2.943408	-0.77874374	0.77874374
222167_at			Hs.665725		-2.9455178	-0.77926064	0.77926064
227121_at			Hs.193784		-2.9456785	-0.7793002	0.77929974
211071_s_at	10962	MLLT11	Hs.75823	myeloid/lymphoid or mixed-lineage leuke	-2.9481456	-0.77990437	0.7799034
203505_at	19	ABCA1	Hs.429294	ATP-binding cassette, sub-family A (ABC	-2.950426	-0.78046155	0.7804618
203729_at	2014	EMP3	Hs.9999	epithelial membrane protein 3	-2.9509766	-0.7805958	0.78059673
219383_at	79899	PRR5L	Hs.19987	proline rich 5 like	-2.9547	-0.78150606	0.7815056
215092_s_at	10725	NFAT5	Hs.371987	nuclear factor of activated T-cells 5, tonic	-2.955164	-0.7816191	0.7816191
239923_at			Hs.666935		-2.957719	-0.7822423	0.7822428
242162_at	164781	WDR69	Hs.424594	WD repeat domain 69	-2.9598246	-0.78275585	0.78275585
244026_at			Hs.604381		-2.9624896	-0.7834053	0.7834048
236277_at			Hs.655757		-2.9644663	-0.78388643	0.78388596
219737_s_at	5101	PCDH9	Hs.654709	protocadherin 9	-2.964779	-0.78396225	0.78396225
201720_s_at	7805	LAPTM5	Hs.371021	lysosomal protein transmembrane 5	-2.9651318	-0.7840481	0.7840481
226542_at			Hs.547576		-2.9725971	-0.78586197	0.78586197
223502_s_at	10673	TNFSF13B	Hs.525157	tumor necrosis factor (ligand) superfamily	-2.9735641	-0.7860966	0.7860966
209921_at	23657	SLC7A11	Hs.390594	solute carrier family 7, (cationic amino ac	-2.9761717	-0.78672886	0.78672886
224733_at	123920	CMTM3	Hs.298198	CKLF-like MARVEL transmembrane dor	-2.987701	-0.7895179	0.7895179
210896_s_at	444	ASPH	Hs.332422	aspartate beta-hydroxylase	-2.9956033	-0.79142284	0.7914238
222073_at	1285	COL4A3	Hs.570065	collagen, type IV, alpha 3 (Goodpasture a	-2.999398	-0.79233646	0.79233646
224772_at	89796	NAV1	Hs.585374	neuron navigator 1	-2.999505	-0.7923622	0.7923622
210664_s_at	7035	TFPI	Hs.516578	tissue factor pathway inhibitor (lipoprotei	-3.0007186	-0.79265404	0.79265404
211919_s_at	7852	CXCR4	Hs.593413	chemokine (C-X-C motif) receptor 4	-3.0042436	-0.7935009	0.7935009
225368_at	28996	HIPK2	Hs.724392	homeodomain interacting protein kinase 2	-3.0051334	-0.7937145	0.7937145
239556_at	645513	LOC645513	Hs.709491	Hypothetical LOC645513	-3.0067708	-0.79410744	0.79410744
229088_at	5167	ENPP1	Hs.527295	ectonucleotide pyrophosphatase/phosphoc	-3.0079706	-0.79439497	0.79439545
201718_s_at	2037	EPB41L2	Hs.486470	erythrocyte membrane protein band 4.1-lil	-3.0100121	-0.7948847	0.7948847
235421_at	1326	MAP3K8	Hs.432453///Hs.6630	Mitogen-activated protein kinase kinase k	-3.0160153	-0.79632187	0.79632187
201482_at	5768	QSOX1	Hs.518374	quiescin Q6 sulfhydryl oxidase 1	-3.0177941	-0.7967472	0.7967472
231406_at	80228	ORAI2	Hs.363308	ORAI calcium release-activated calcium r	-3.0295057	-0.799541	0.7995415
215447_at					-3.0308628	-0.7998643	0.7998643
226250_at			Hs.202577		-3.0361707	-0.8011265	0.8011265
205880_at	5587	PRKD1	Hs.508999	protein kinase D1	-3.0366135	-0.80123186	0.8012314
229040_at			Hs.661035		-3.0429099	-0.8027258	0.8027258
202459_s_at	9663	LPIN2	Hs.132342	lipin 2	-3.0430546	-0.8027601	0.8027601
222783_s_at	64093	SMOC1	Hs.497349	SPARC related modular calcium binding	-3.044435	-0.80308723	0.80308723
204385_at	8942	KYNU	Hs.470126	kynureninase (L-kynurenine hydrolase)	-3.047171	-0.80373573	0.8037348
236067_at			Hs.662697		-3.0479023	-0.80390835	0.80390835
228742_at			Hs.133261		-3.048793	-0.8041191	0.8041191
208963_x_at	3992	FADS1	Hs.503546	fatty acid desaturase 1	-3.0515554	-0.8047724	0.8047724
214719_at	283537	SLC46A3	Hs.117167	solute carrier family 46, member 3	-3.0608122	-0.80695724	0.80695724

237411_at	11174	ADAMTS6	Hs.482291	ADAM metallopeptidase with thrombospo	-3.062665	-0.80739355	0.807394
203474_at	10788	IQGAP2	Hs.291030	IQ motif containing GTPase activating pro	-3.0630364	-0.8074813	0.8074813
214110_s_at			Hs.469287		-3.0637028	-0.80763817	0.80763817
209616_s_at	1066	CES1	Hs.558865	carboxylesterase 1	-3.0684211	-0.80874825	0.80874825
1560318_at	9411	ARHGAP29	Hs.483238	Rho GTPase activating protein 29	-3.0686178	-0.8087945	0.8087945
202796_at	11346	SYNPO	Hs.723138	synaptopodin	-3.0708814	-0.8093262	0.80932665
231292_at	493861	EID3	Hs.659857	EP300 interacting inhibitor of differentiat	-3.0711246	-0.80938363	0.8093834
229201_at			Hs.593599		-3.0722134	-0.809639	0.80963945
202369_s_at	9697	TRAM2	Hs.520182	translocation associated membrane protein	-3.074175	-0.8100996	0.8100996
214439_x_at	274	BIN1	Hs.193163	bridging integrator 1	-3.0782132	-0.8110466	0.8110466
213187_x_at	2512	FTL	Hs.433670	ferritin, light polypeptide	-3.07837	-0.8110838	0.81108284
239043_at	342908	ZNF404	Hs.76561	zinc finger protein 404	-3.0805507	-0.81159425	0.811594
221107_at	55584	CHRNA9	Hs.272278	cholinergic receptor, nicotinic, alpha 9	-3.0826585	-0.81208754	0.81208754
239343_at	728705	LOC728705	Hs.129828	hypothetical protein LOC728705	-3.0833738	-0.8122549	0.8122549
209651_at	7041	TGFB1I1	Hs.513530	transforming growth factor beta 1 induced	-3.0883102	-0.81340885	0.81340885
243813_at			Hs.583755		-3.092674	-0.8144274	0.8144274
238429_at	137835	TMEM71	Hs.293842	transmembrane protein 71	-3.0930235	-0.8145089	0.8145089
212327_at	22998	LIMCH1	Hs.335163	LIM and calponin homology domains 1	-3.0952797	-0.81503487	0.81503487
203910_at	9411	ARHGAP29	Hs.483238	Rho GTPase activating protein 29	-3.0994031	-0.8159952	0.8159952
224990_at	201895	C4orf34	Hs.576320	chromosome 4 open reading frame 34	-3.1010222	-0.8163719	0.8163719
229441_at	11098	PRSS23	Hs.25338	Protease, serine, 23	-3.1038768	-0.8170357	0.8170357
205534_at	5099	PCDH7	Hs.479439	protocadherin 7	-3.1092603	-0.81828547	0.81828594
202975_s_at	22836	RHOBTB3	Hs.445030	Rho-related BTB domain containing 3	-3.113133	-0.8191838	0.81918335
241363_at	54932	EXD3	Hs.495553	exonuclease 3'-5' domain containing 3	-3.1136239	-0.8192973	0.8192973
202931_x_at	274	BIN1	Hs.193163	bridging integrator 1	-3.122306	-0.82130575	0.8213062
202665_s_at	7456	WIPF1	Hs.128067	WAS/WASL interacting protein family, m	-3.1241229	-0.82172537	0.82172585
235629_at			Hs.658355		-3.1287796	-0.8227999	0.82280016
211677_x_at	57863	CADM3	Hs.365689	cell adhesion molecule 3	-3.1377578	-0.8248668	0.82486725
205464_at	6338	SCNN1B	Hs.414614	sodium channel, nonvoltage-gated 1, beta	-3.139427	-0.8252506	0.8252506
232268_at			Hs.656897		-3.1416602	-0.82576346	0.8257637
211571_s_at	1462	VCAN	Hs.643801	versican	-3.1510887	-0.8279252	0.8279252
243606_at	91775	FAM55C	Hs.595933	family with sequence similarity 55, memb	-3.156457	-0.82915306	0.82915306
205372_at	5324	PLAG1	Hs.14968	pleiomorphic adenoma gene 1	-3.1608088	-0.830147	0.8301468
226103_at	91624	NEXN	Hs.612385	nexilin (F actin binding protein)	-3.164146	-0.8309083	0.8309078
237313_at			Hs.666723		-3.1666474	-0.8314781	0.8314781
229072_at			Hs.594773		-3.1786456	-0.8342061	0.8342061
230180_at					-3.1801903	-0.8345566	0.8345566
201280_s_at	1601	DAB2	Hs.481980	disabled homolog 2, mitogen-responsive p	-3.181635	-0.83488417	0.83488417
228964_at	639	PRDM1	Hs.436023	PR domain containing 1, with ZNF domai	-3.182873	-0.83516455	0.835165
208962_s_at	3992	FADS1	Hs.503546	fatty acid desaturase 1	-3.1830087	-0.83519554	0.83519554
230744_at	11167	FSTL1	Hs.269512	follistatin-like 1	-3.1955926	-0.8380418	0.8380418
231929_at	22807	IKZF2	Hs.604950	IKAROS family zinc finger 2 (Helios)	-3.2020485	-0.83949757	0.83949757
226333_at	3570	IL6R	Hs.709210	interleukin 6 receptor	-3.2031155	-0.8397379	0.8397379

240771_at	257044	C1orf101	Hs.459534	chromosome 1 open reading frame 101	-3.2046566	-0.8400848	0.840085
58780_s_at	55701	ARHGEF40	Hs.35125	Rho guanine nucleotide exchange factor (	-3.2180438	-0.84309196	0.84309196
205141_at	283	ANG	Hs.283749	angiogenin, ribonuclease, RNase A family	-3.2182617	-0.8431411	0.8431406
210201_x_at	274	BIN1	Hs.193163	bridging integrator 1	-3.226419	-0.8449669	0.8449669
244745_at	85004	RERG	Hs.199487	RAS-like, estrogen-regulated, growth inhi	-3.238162	-0.8475876	0.8475876
213069_at	57493	HEG1	Hs.477420	HEG homolog 1 (zebrafish)	-3.242086	-0.84846115	0.84846115
213455_at	92689	FAM114A1	Hs.476517	family with sequence similarity 114, mem	-3.2426174	-0.8485794	0.8485794
213241_at	10154	PLXNC1	Hs.584845	plexin C1	-3.2435231	-0.8487811	0.84878063
205640_at	221	ALDH3B1	Hs.523841	aldehyde dehydrogenase 3 family, membe	-3.245933	-0.8493166	0.8493166
212325_at	22998	LIMCH1	Hs.335163	LIM and calponin homology domains 1	-3.2459898	-0.8493295	0.849329
204620_s_at	1462	VCAN	Hs.643801	versican	-3.251061	-0.8504553	0.8504553
210757_x_at	1601	DAB2	Hs.481980	disabled homolog 2, mitogen-responsive p	-3.2514951	-0.8505516	0.8505516
44702_at	85360	SYDE1	Hs.528701	synapse defective 1, Rho GTPase, homolo	-3.2544453	-0.8512058	0.8512058
224016_at	28996	HIPK2	Hs.724392	homeodomain interacting protein kinase 2	-3.259257	-0.85227156	0.85227156
210220_at	2535	FZD2	Hs.142912	frizzled homolog 2 (Drosophila)	-3.2704082	-0.8547354	0.8547354
225097_at	28996	HIPK2	Hs.724392	homeodomain interacting protein kinase 2	-3.27674	-0.8561306	0.8561306
228390_at			Hs.594773		-3.2790487	-0.85663843	0.8566389
227584_at	89796	NAV1	Hs.585374	neuron navigator 1	-3.2841346	-0.8577566	0.8577566
206662_at	2745	GLRX	Hs.28988	glutaredoxin (thioltransferase)	-3.285507	-0.858058	0.858058
1560011_at					-3.2889445	-0.85881233	0.85881233
219093_at	55022	PID1	Hs.724663	phosphotyrosine interaction domain conta	-3.2904232	-0.8591366	0.8591366
1558378_a_at	113146	AHNAK2	Hs.441783	AHNAK nucleoprotein 2	-3.2915328	-0.85937977	0.85937977
227647_at	10008	KCNE3	Hs.523899	potassium voltage-gated channel, Isk-relat	-3.2916741	-0.85941076	0.85941076
1556997_a_at	3953	LEPR	Hs.723178	leptin receptor	-3.291711	-0.85941887	0.85941887
219557_s_at	56675	NRIP3	Hs.523467	nuclear receptor interacting protein 3	-3.3021297	-0.86169815	0.8616986
211964_at	1284	COL4A2	Hs.508716	collagen, type IV, alpha 2	-3.3067245	-0.8627014	0.8627014
201397_at	26227	PHGDH	Hs.487296	phosphoglycerate dehydrogenase	-3.3139458	-0.864275	0.864275
215641_at	9871	SEC24D	Hs.189641	SEC24 family, member D (S. cerevisiae)	-3.3345804	-0.8687527	0.8687525
225116_at	28996	HIPK2	Hs.724392	homeodomain interacting protein kinase 2	-3.3422682	-0.8704138	0.8704138
204421_s_at	2247	FGF2	Hs.284244	fibroblast growth factor 2 (basic)	-3.3474014	-0.8715205	0.871521
209866_s_at	23284	LPHN3	Hs.570770	latrophilin 3	-3.349472	-0.87196684	0.87196684
228919_at					-3.3528712	-0.8726983	0.8726988
220935_s_at	55755	CDK5RAP2	Hs.269560	CDK5 regulatory subunit associated prote	-3.3550851	-0.87317467	0.87317467
243707_at			Hs.145520		-3.3633323	-0.87494564	0.87494564
226546_at	100506844	LOC100506844		hypothetical LOC100506844	-3.365841	-0.8754835	0.8754835
227503_at			Hs.656767		-3.3664374	-0.8756113	0.8756113
212328_at	22998	LIMCH1	Hs.335163	LIM and calponin homology domains 1	-3.3846943	-0.8795128	0.8795128
219028_at	28996	HIPK2	Hs.724392	homeodomain interacting protein kinase 2	-3.387488	-0.8801079	0.8801079
1567575_at			Hs.684651		-3.394602	-0.8816211	0.88162136
211980_at	1282	COL4A1	Hs.17441	collagen, type IV, alpha 1	-3.3970404	-0.8821392	0.8821392
228275_at			Hs.551751		-3.415257	-0.8859973	0.8859968
227052_at			Hs.720403		-3.4271197	-0.8884983	0.8884983
204619_s_at	1462	VCAN	Hs.643801	versican	-3.4310095	-0.88931656	0.88931656

219714_s_at	55799	CACNA2D3	Hs.656687	calcium channel, voltage-dependent, alpha	-3.4345267	-0.89005566	0.89005566
208161_s_at	8714	ABCC3	Hs.463421	ATP-binding cassette, sub-family C (CFT	-3.4432263	-0.891881	0.89188004
203989_x_at	2149	F2R	Hs.482562	coagulation factor II (thrombin) receptor	-3.453564	-0.89404297	0.89404297
236798_at					-3.4606712	-0.89552593	0.89552593
221731_x_at	1462	VCAN	Hs.643801	versican	-3.4669955	-0.89684296	0.89684296
235004_at	221662	RBM24	Hs.519904	RNA binding motif protein 24	-3.4705908	-0.89759064	0.89759064
230527_at			Hs.127274		-3.4808242	-0.89971447	0.89971447
222945_x_at	55301	OLAH	Hs.24309	oleoyl-ACP hydrolase	-3.4819427	-0.8999462	0.8999462
223402_at	54935	DUSP23	Hs.425801	dual specificity phosphatase 23	-3.4848855	-0.9005556	0.9005556
243996_at					-3.4866688	-0.9009247	0.9009247
228754_at	6533	SLC6A6	Hs.529488	solute carrier family 6 (neurotransmitter tr	-3.4873557	-0.9010668	0.9010668
218418_s_at	25959	KANK2	Hs.284208	KN motif and ankyrin repeat domains 2	-3.495369	-0.90272236	0.90272236
203708_at	5142	PDE4B	Hs.198072	phosphodiesterase 4B, cAMP-specific	-3.4993258	-0.9035382	0.9035387
1563077_at	100289058	LOC100289058	Hs.348525	Hypothetical protein LOC100289058	-3.5039759	-0.90449667	0.9044962
226433_at	114804	RNF157	Hs.500643	ring finger protein 157	-3.5047972	-0.90466547	0.90466547
209086_x_at	4162	MCAM	Hs.599039	melanoma cell adhesion molecule	-3.5142055	-0.9065995	0.90659904
205945_at	3570	IL6R	Hs.709210	interleukin 6 receptor	-3.5157902	-0.9069247	0.90692425
230435_at	375190	LOC375190	Hs.710370	hypothetical protein LOC375190	-3.53277	-0.9103999	0.9103999
228132_at	84448	ABLIM2	Hs.233404	actin binding LIM protein family, membe	-3.543763	-0.91264105	0.91264105
205960_at	5166	PDK4	Hs.8364	pyruvate dehydrogenase kinase, isozyme 4	-3.5472012	-0.91334057	0.91334057
229838_at	4925	NUCB2	Hs.654599	nucleobindin 2	-3.5481098	-0.9135251	0.9135256
1558199_at	2335	FN1	Hs.203717	fibronectin 1	-3.5556386	-0.9150543	0.9150543
1557227_s_at	7175	TPR	Hs.279640	translocated promoter region (to activated	-3.5557148	-0.91507006	0.9150696
206742_at	2277	FIGF	Hs.11392	c-fos induced growth factor (vascular end	-3.5676363	-0.9174843	0.9174843
236599_at					-3.5686176	-0.91768265	0.91768265
242324_x_at	147372	CCBE1	Hs.34333	collagen and calcium binding EGF domai	-3.5705643	-0.91807604	0.91807604
201272_at	231	AKR1B1	Hs.521212	aldo-keto reductase family 1, member B1	-3.5832424	-0.9206333	0.92063236
243974_at			Hs.648410		-3.5924802	-0.9224901	0.9224901
219928_s_at	26256	CABYR	Hs.511983	calcium binding tyrosine-(Y)-phosphoryla	-3.5977407	-0.92354584	0.92354536
212298_at	8829	NRP1	Hs.131704	neuropilin 1	-3.612842	-0.9265671	0.9265671
203921_at	9435	CHST2	Hs.8786	carbohydrate (N-acetylglucosamine-6-O)	-3.619918	-0.9279785	0.9279785
243805_at	147372	CCBE1	Hs.34333	collagen and calcium binding EGF domai	-3.635962	-0.93116856	0.93116856
203665_at	3162	HMOX1	Hs.517581	heme oxygenase (decycling) 1	-3.637289	-0.9314318	0.9314318
205027_s_at	1326	MAP3K8	Hs.432453	mitogen-activated protein kinase kinase k	-3.643482	-0.9326587	0.93265915
242868_at			Hs.691259		-3.6444454	-0.9328494	0.9328499
1552309_a_at	91624	NEXN	Hs.612385	nexilin (F actin binding protein)	-3.6536345	-0.93466616	0.93466616
242809_at	9173	IL1RL1	Hs.66	interleukin 1 receptor-like 1	-3.6606114	-0.9360423	0.9360423
238919_at			Hs.656886		-3.662952	-0.9365034	0.9365034
1559739_at	56994	CHPT1	Hs.293077	Choline phosphotransferase 1	-3.6638482	-0.93667984	0.93667984
227396_at	5795	PTPRJ	Hs.318547	protein tyrosine phosphatase, receptor typ	-3.6659932	-0.93710184	0.9371023
219049_at	55790	CSGALNACT1	Hs.613729	chondroitin sulfate N-acetylgalactosaminy	-3.6712813	-0.9381418	0.9381418
213338_at	25907	TMEM158	Hs.35861	transmembrane protein 158 (gene/pseudog	-3.6759012	-0.93904924	0.93904877
231879_at	1303	COL12A1	Hs.101302	collagen, type XII, alpha 1	-3.6779783	-0.93945646	0.93945646

240815_at			Hs.657583		-3.6852038	-0.9408722	0.9408722
203186_s_at	6275	S100A4	Hs.654444	S100 calcium binding protein A4	-3.6908305	-0.94197273	0.94197273
214078_at			Hs.655757		-3.6920786	-0.9422164	0.9422169
209101_at	1490	CTGF	Hs.410037	connective tissue growth factor	-3.6957524	-0.94293404	0.94293404
209210_s_at	10979	FERMT2	Hs.509343	fermitin family member 2	-3.6994383	-0.9436531	0.9436531
236264_at	23284	LPHN3	Hs.28391	latrophilin 3	-3.7222154	-0.948081	0.94808054
209209_s_at	10979	FERMT2	Hs.509343	fermitin family member 2	-3.728553	-0.94930744	0.9493084
203058_s_at	9060	PAPSS2	Hs.524491	3'-phosphoadenosine 5'-phosphosulfate sy	-3.7406175	-0.9516382	0.9516382
241837_at					-3.7443678	-0.9523611	0.9523611
228482_at	284040	CDRT4	Hs.164595	CMT1A duplicated region transcript 4	-3.7514734	-0.9537287	0.9537287
1553772_at	256356	GK5	Hs.135904	glycerol kinase 5 (putative)	-3.7630088	-0.9559431	0.9559436
213156_at			Hs.592414		-3.7672536	-0.9567566	0.9567566
228702_at	378805	FLJ43663	Hs.702887	hypothetical LOC378805	-3.7708027	-0.9574361	0.9574356
204439_at	10964	IFI44L	Hs.724492	interferon-induced protein 44-like	-3.7758799	-0.95840645	0.95840645
217678_at	23657	SLC7A11	Hs.390594	solute carrier family 7, (cationic amino ac	-3.7840807	-0.9599714	0.9599714
207980_s_at	10370	CITED2	Hs.82071	Cbp/p300-interacting transactivator, with	-3.787299	-0.96058464	0.96058464
1557437_a_at			Hs.655836		-3.8220406	-0.96717143	0.96717167
52837_at	85352	KIAA1644	Hs.6829	KIAA1644	-3.8331602	-0.9692669	0.96926737
213158_at			Hs.592414		-3.8353221	-0.96967363	0.9696741
210663_s_at	8942	KYNU	Hs.470126	kynureninase (L-kynurenine hydrolase)	-3.8372912	-0.97004414	0.97004414
223378_at	84662	GLIS2	Hs.592087	GLIS family zinc finger 2	-3.861633	-0.97460556	0.97460556
209357_at	10370	CITED2	Hs.82071	Cbp/p300-interacting transactivator, with	-3.8707416	-0.976305	0.976305
211737_x_at	5764	PTN	Hs.371249	pleiotrophin	-3.903916	-0.982461	0.982461
201266_at	7296	TXNRD1	Hs.724443	thioredoxin reductase 1	-3.9068975	-0.9830122	0.98301125
244455_at	343450	KCNT2	Hs.657046	potassium channel, subfamily T, member	-3.9110947	-0.98378634	0.9837861
234066_at	9173	IL1RL1	Hs.66	interleukin 1 receptor-like 1	-3.939327	-0.9889746	0.9889746
209183_s_at	11067	C10orf10	Hs.93675	chromosome 10 open reading frame 10	-3.94229	-0.9895172	0.98951674
1552658_a_at	89795	NAV3	Hs.655301	neuron navigator 3	-3.945133	-0.99003696	0.99003696
215446_s_at	4015	LOX	Hs.102267	lysyl oxidase	-3.947794	-0.99052334	0.99052334
206090_s_at	100303453 /// 27	DISC1 /// TSNA	Hs.13318	disrupted in schizophrenia 1 /// TSNAX-I	-3.9524183	-0.9913678	0.9913678
1563467_at			Hs.683994		-3.9532714	-0.99152374	0.99152327
37408_at	9902	MRC2	Hs.7835	mannose receptor, C type 2	-3.9624612	-0.9931984	0.9931984
200665_s_at	6678	SPARC	Hs.111779	secreted protein, acidic, cysteine-rich (ost	-3.9719334	-0.99492073	0.99492073
202437_s_at	1545	CYP1B1	Hs.154654	cytochrome P450, family 1, subfamily B,	-3.9883142	-0.9978895	0.9978895
239336_at					-3.9947996	-0.9990616	0.9990616
224989_at			Hs.720403		-3.999799	-0.99996376	0.99996376
219295_s_at	26577	PCOLCE2	Hs.8944	procollagen C-endopeptidase enhancer 2	-4.016316	-1.0029364	1.0029364
209276_s_at	2745	GLRX	Hs.28988	glutaredoxin (thioltransferase)	-4.0381775	-1.0068521	1.0068521
201069_at	4313	MMP2	Hs.513617	matrix metallopeptidase 2 (gelatinase A, 7	-4.045655	-1.0081863	1.0081868
215646_s_at	1462	VCAN	Hs.643801	versican	-4.052405	-1.0093894	1.0093889
209890_at	10098	TSPAN5	Hs.118118	tetraspanin 5	-4.0572014	-1.0102425	1.0102425
57715_at	51063	CALHM2	Hs.241545	calcium homeostasis modulator 2	-4.0677714	-1.0121193	1.0121193
210764_s_at	3491	CYR61	Hs.8867	cysteine-rich, angiogenic inducer, 61	-4.078843	-1.01408	1.01408

222719_s_at	56034	PDGFC	Hs.570855	platelet derived growth factor C	-4.0797453	-1.0142398	1.0142393
220334_at	26575	RGS17	Hs.166313	regulator of G-protein signaling 17	-4.0985465	-1.0175562	1.0175562
202214_s_at	8450	CUL4B	Hs.102914	cullin 4B	-4.108358	-1.0192814	1.0192804
214621_at	2998	GYS2	Hs.82614	glycogen synthase 2 (liver)	-4.1269274	-1.0225341	1.0225339
239367_at	627	BDNF	Hs.502182	brain-derived neurotrophic factor	-4.131758	-1.0233779	1.0233779
203889_at	6447	SCG5	Hs.156540	secretogranin V (7B2 protein)	-4.1329737	-1.0235901	1.0235901
201278_at	1601	DAB2	Hs.481980	disabled homolog 2, mitogen-responsive p	-4.133422	-1.0236683	1.0236683
203895_at	5332	PLCB4	Hs.472101	phospholipase C, beta 4	-4.1431165	-1.0253582	1.0253582
202436_s_at	1545	CYP1B1	Hs.154654	cytochrome P450, family 1, subfamily B,	-4.158185	-1.027977	1.027977
226614_s_at	83648	FAM167A	Hs.124299	family with sequence similarity 167, mem	-4.164523	-1.0290756	1.0290756
209641_s_at	8714	ABCC3	Hs.463421	ATP-binding cassette, sub-family C (CFT	-4.1782217	-1.0314445	1.0314445
222880_at	10000	AKT3	Hs.498292	v-akt murine thymoma viral oncogene hor	-4.178574	-1.0315056	1.0315051
1552607_at	100132967	NCRNA00204	Hs.391764	non-protein coding RNA 204	-4.182482	-1.0321798	1.0321794
202765_s_at	2200	FBN1	Hs.591133	fibrillin 1	-4.1952662	-1.0343809	1.0343814
218651_s_at	55323	LARP6	Hs.416755	La ribonucleoprotein domain family, mem	-4.207294	-1.0364461	1.0364466
233223_at					-4.20976	-1.036869	1.036869
202948_at	3554	IL1R1	Hs.701982	interleukin 1 receptor, type I	-4.2097993	-1.0368757	1.0368757
229487_at	1879	EBF1	Hs.573143	early B-cell factor 1	-4.218322	-1.0383348	1.0383344
1554867_a_at	51334	PRR16	Hs.157461	proline rich 16	-4.229029	-1.040163	1.0401635
207624_s_at	6103	RPGR	Hs.61438	retinitis pigmentosa GTPase regulator	-4.236314	-1.0414047	1.0414047
204575_s_at	4327	MMP19	Hs.591033	matrix metallopeptidase 19	-4.243842	-1.0426855	1.0426855
205110_s_at	2258	FGF13	Hs.6540	fibroblast growth factor 13	-4.252985	-1.0442376	1.0442381
204105_s_at	4897	NRCAM	Hs.21422	neuronal cell adhesion molecule	-4.2648892	-1.0462542	1.0462542
209201_x_at	7852	CXCR4	Hs.593413	chemokine (C-X-C motif) receptor 4	-4.291463	-1.050735	1.0507345
202435_s_at	1545	CYP1B1	Hs.154654	cytochrome P450, family 1, subfamily B,	-4.311576	-1.0541077	1.0541077
232125_at			Hs.587185		-4.325182	-1.0563805	1.0563803
212912_at	6196	RPS6KA2	Hs.655277	ribosomal protein S6 kinase, 90kDa, polyp	-4.327326	-1.0567379	1.0567379
1559400_s_at	5069	PAPPA	Hs.643599	pregnancy-associated plasma protein A, p	-4.338032	-1.0585203	1.0585203
212423_at	219654	ZCCHC24	Hs.523080	zinc finger, CCHC domain containing 24	-4.353418	-1.0610743	1.0610743
225540_at	4133	MAP2	Hs.368281	microtubule-associated protein 2	-4.369956	-1.0638094	1.0638094
227240_at	25791	NGEF	Hs.97316	neuronal guanine nucleotide exchange fac	-4.3715973	-1.0640802	1.0640802
204151_x_at	1645	AKR1C1	Hs.460260	aldo-keto reductase family 1, member C1	-4.38181	-1.0657635	1.0657635
228640_at	5099	PCDH7	Hs.479439	protocadherin 7	-4.3956337	-1.0680356	1.0680356
204140_at	8460	TPST1	Hs.724538	tyrosylprotein sulfotransferase 1	-4.4079275	-1.0700502	1.0700502
1561079_at	23243	ANKRD28	Hs.335239	ankyrin repeat domain 28	-4.4213777	-1.072248	1.072248
226953_at	2532 /// 57863	CADM3 /// DAF	Hs.153381	cell adhesion molecule 3 /// Duffy blood g	-4.435109	-1.0744848	1.0744848
232882_at			Hs.687769		-4.453552	-1.0774779	1.0774784
228737_at	84969	TOX2	Hs.26608	TOX high mobility group box family men	-4.454248	-1.077591	1.077591
203896_s_at	5332	PLCB4	Hs.472101	phospholipase C, beta 4	-4.4549665	-1.0777073	1.0777073
220407_s_at	7042	TGFB2	Hs.133379	transforming growth factor, beta 2	-4.470371	-1.0801971	1.0801973
205158_at	6038	RNASE4		ribonuclease, RNase A family, 4	-4.482862	-1.0822101	1.0822101
212494_at	23371	TENC1	Hs.6147	tensin like C1 domain containing phospha	-4.4829793	-1.0822291	1.0822287
220180_at	80323	CCDC68	Hs.120790	coiled-coil domain containing 68	-4.49581	-1.0842905	1.0842905

218332_at	55859	BEX1	Hs.334370	brain expressed, X-linked 1	-4.49914	-1.0848246	1.0848246
210662_at	8942	KYNU	Hs.470126	kynureninase (L-kynurenine hydrolase)	-4.5029926	-1.0854421	1.0854421
203939_at	4907	NT5E	Hs.153952	5'-nucleotidase, ecto (CD73)	-4.505145	-1.0857868	1.0857868
205767_at	2069	EREG	Hs.115263	epiregulin	-4.505969	-1.0859189	1.0859184
229041_s_at			Hs.661035		-4.5194745	-1.0880775	1.0880775
238127_at	650669	FLJ41484	Hs.720848	hypothetical LOC650669	-4.5344057	-1.090457	1.0904565
210015_s_at	4133	MAP2	Hs.368281	microtubule-associated protein 2	-4.5394306	-1.0912557	1.0912557
235392_at			Hs.720470		-4.5547857	-1.0936918	1.0936913
231146_at	196792	FAM24B	Hs.114648	family with sequence similarity 24, memb	-4.5707726	-1.0962191	1.0962191
204236_at	2313	FLI1	Hs.504281	Friend leukemia virus integration 1	-4.593344	-1.0997725	1.0997725
209699_x_at	1646	AKR1C2	Hs.567256	aldo-keto reductase family 1, member C2	-4.598044	-1.1005106	1.1005096
203217_s_at	8869	ST3GAL5	Hs.415117	ST3 beta-galactoside alpha-2,3-sialyltrans	-4.598802	-1.1006293	1.1006289
209581_at	11145	PLA2G16	Hs.502775	phospholipase A2, group XVI	-4.604574	-1.1015339	1.1015339
225115_at	28996	HIPK2	Hs.724392	homeodomain interacting protein kinase 2	-4.619086	-1.1038036	1.1038036
212419_at	219654	ZCCHC24	Hs.523080	zinc finger, CCHC domain containing 24	-4.6268353	-1.1050129	1.1050129
204115_at	2791	GNG11	Hs.83381	guanine nucleotide binding protein (G prc	-4.6320624	-1.1058273	1.1058273
1555538_s_at	171483	FAM9B	Hs.371894	family with sequence similarity 9, membe	-4.6327868	-1.1059403	1.1059399
213429_at	80114	BICC1	Hs.158745	bicaudal C homolog 1 (Drosophila)	-4.638699	-1.1068602	1.1068602
207264_at	11015	KDELR3	Hs.720434	KDEL (Lys-Asp-Glu-Leu) endoplasmic re	-4.687123	-1.1143513	1.1143513
215209_at	9871	SEC24D	Hs.189641	SEC24 family, member D (S. cerevisiae)	-4.688786	-1.1146071	1.1146073
203440_at	1000	CDH2	Hs.464829	cadherin 2, type 1, N-cadherin (neuronal)	-4.7355256	-1.1217623	1.1217623
217764_s_at	11031	RAB31	Hs.724447	RAB31, member RAS oncogene family	-4.739838	-1.1224189	1.1224189
205830_at	1047	CLGN	Hs.86368	calmegin	-4.7823224	-1.1288557	1.1288557
206157_at	5806	PTX3	Hs.591286	pentraxin 3, long	-4.8269663	-1.1355586	1.1355581
225516_at	6542	SLC7A2	Hs.448520	solute carrier family 7 (cationic amino aci	-4.8751225	-1.1427193	1.1427193
224341_x_at	7099	TLR4	Hs.174312	toll-like receptor 4	-4.8986044	-1.1461854	1.1461854
206758_at	1907	EDN2	Hs.1407	endothelin 2	-4.945216	-1.153017	1.1530166
213325_at	25945	PVRL3	Hs.293917	poliovirus receptor-related 3	-4.9791102	-1.1579442	1.1579437
225207_at	5166	PDK4	Hs.8364	pyruvate dehydrogenase kinase, isozyme 4	-4.9864635	-1.159009	1.159008
212886_at	26112	CCDC69	Hs.655336	coiled-coil domain containing 69	-4.993592	-1.160039	1.160039
209160_at	8644	AKR1C3	Hs.78183	aldo-keto reductase family 1, member C3	-5.0164933	-1.1633396	1.1633396
205226_at	5157	PDGFRL	Hs.458573	platelet-derived growth factor receptor-lik	-5.017085	-1.163425	1.1634245
213397_x_at	6038	RNASE4		ribonuclease, RNase A family, 4	-5.0219197	-1.1641192	1.1641197
214212_x_at	10979	FERMT2	Hs.509343	fermitin family member 2	-5.036897	-1.1662679	1.1662674
235030_at	91775	FAM55C	Hs.595933	family with sequence similarity 55, memb	-5.0536375	-1.1686611	1.1686611
220301_at	79839	CCDC102B	Hs.280781	coiled-coil domain containing 102B	-5.056848	-1.1691191	1.1691194
215330_at			Hs.671957		-5.0644193	-1.1701984	1.1701984
204686_at	3667	IRS1	Hs.471508	insulin receptor substrate 1	-5.077479	-1.1720562	1.1720562
227529_s_at	9590	AKAP12	Hs.371240	A kinase (PRKA) anchor protein 12	-5.137154	-1.1804845	1.1804848
202149_at	4739	NEDD9	Hs.37982	neural precursor cell expressed, developm	-5.1494846	-1.1822138	1.1822143
1557278_s_at	3842	TNPO1	Hs.482497	Transportin 1	-5.1582336	-1.1834388	1.1834383
239968_at	114038	C21orf84	Hs.592161	chromosome 21 open reading frame 84	-5.1589613	-1.1835403	1.1835403
205407_at	8434	RECK	Hs.388918	reversion-inducing-cysteine-rich protein v	-5.161083	-1.1838369	1.1838369

231766_s_at	1303	COL12A1	Hs.101302	collagen, type XII, alpha 1	-5.1843004	-1.1870747	1.1870747
232918_at	541471	LOC541471	Hs.652426	Hypothetical LOC541471	-5.1997104	-1.1892157	1.1892157
202213_s_at	8450	CUL4B	Hs.102914	cullin 4B	-5.219067	-1.1918955	1.1918964
1555097_a_at	5737	PTGFR	Hs.654365	prostaglandin F receptor (FP)	-5.23313	-1.1938369	1.1938372
243357_at	257194	NEGR1	Hs.146542	neuronal growth regulator 1	-5.2449856	-1.1954694	1.1954694
210119_at	3772	KCNJ15	Hs.411299	potassium inwardly-rectifying channel, su	-5.2961936	-1.2024779	1.2024779
210257_x_at	8450	CUL4B	Hs.102914	cullin 4B	-5.335469	-1.2078075	1.2078075
231175_at	221336	BEND6	Hs.582993	BEN domain containing 6	-5.348047	-1.209506	1.209506
203875_at	6594	SMARCA1	Hs.152292	SWI/SNF related, matrix associated, actin	-5.371274	-1.2126322	1.2126322
213413_at	11037	STON1	Hs.44385	stonin 1	-5.3801465	-1.2138226	1.2138228
238688_at	7168	TPM1	Hs.133892	Tropomyosin 1 (alpha)	-5.4650526	-1.2251177	1.2251177
237290_at			Hs.658819		-5.474895	-1.2264156	1.2264156
206766_at	8515	ITGA10	Hs.158237	integrin, alpha 10	-5.4789715	-1.2269526	1.2269526
204955_at	8406	SRPX	Hs.15154	sushi-repeat-containing protein, X-linked	-5.480051	-1.2270947	1.2270947
202686_s_at	558	AXL	Hs.590970	AXL receptor tyrosine kinase	-5.5765615	-1.2396879	1.2396879
201721_s_at	7805	LAPTM5	Hs.371021	lysosomal protein transmembrane 5	-5.583127	-1.2405367	1.2405367
1555938_x_at	7431	VIM		vimentin	-5.6303763	-1.2466154	1.2466159
217762_s_at	11031	RAB31	Hs.724447	RAB31, member RAS oncogene family	-5.6349244	-1.2471981	1.2471981
240450_at			Hs.656768		-5.649067	-1.2490063	1.2490063
205082_s_at	316	AOX1	Hs.406238	aldehyde oxidase 1	-5.669987	-1.2516727	1.2516727
228333_at	9839	ZEB2	Hs.34871	zinc finger E-box binding homeobox 2	-5.6914988	-1.2544041	1.2544045
229307_at					-5.8148637	-1.2698727	1.2698727
231227_at	7474	WNT5A	Hs.643085	Wingless-type MMTV integration site fan	-5.8339195	-1.272233	1.2722325
203060_s_at	9060	PAPSS2	Hs.524491	3'-phosphoadenosine 5'-phosphosulfate sy	-5.8584847	-1.2752638	1.2752638
230147_at	2151	F2RL2	Hs.42502	coagulation factor II (thrombin) receptor-l	-5.888788	-1.2789853	1.2789855
204422_s_at	2247	FGF2	Hs.284244	fibroblast growth factor 2 (basic)	-5.9013777	-1.2805257	1.2805262
229778_at	80763	C12orf39	Hs.130692	chromosome 12 open reading frame 39	-5.9346204	-1.2845778	1.2845778
213493_at	25992	SNED1	Hs.471834	sushi, nidogen and EGF-like domains 1	-5.947934	-1.2861943	1.2861943
217763_s_at	11031	RAB31	Hs.724447	RAB31, member RAS oncogene family	-5.971299	-1.2890224	1.2890224
219975_x_at	55301	OLAH	Hs.24309	oleoyl-ACP hydrolase	-5.972932	-1.2892199	1.2892194
209612_s_at	125	ADH1B	Hs.4	alcohol dehydrogenase 1B (class I), beta p	-5.9768834	-1.2896967	1.2896967
223618_at	56776	FMN2	Hs.24889	formin 2	-5.9972563	-1.2921512	1.2921515
204602_at	22943	DKK1	Hs.40499	dickkopf homolog 1 (Xenopus laevis)	-6.0195193	-1.2948246	1.2948236
207177_at	5737	PTGFR	Hs.654365	prostaglandin F receptor (FP)	-6.0206714	-1.2949624	1.2949619
219014_at	51316	PLAC8	Hs.546392	placenta-specific 8	-6.053297	-1.2988605	1.2988605
242002_at	154215	NKAIN2	Hs.656604	Na+/K+ transporting ATPase interacting 2	-6.056795	-1.2992773	1.2992773
208782_at	11167	FSTL1	Hs.269512	follistatin-like 1	-6.081034	-1.3021584	1.3021584
228890_at	84913	ATOH8	Hs.135569	atonal homolog 8 (Drosophila)	-6.102947	-1.3047528	1.3047533
217529_at	80228	ORAI2	Hs.363308	ORAI calcium release-activated calcium r	-6.1059437	-1.3051071	1.3051071
229641_at	147372	CCBE1	Hs.34333	collagen and calcium binding EGF domai	-6.1069365	-1.3052244	1.3052244
217028_at	7852	CXCR4	Hs.593413	chemokine (C-X-C motif) receptor 4	-6.158211	-1.3112559	1.3112555
224822_at	10395	DLC1	Hs.134296	deleted in liver cancer 1	-6.187215	-1.3146453	1.3146448
205896_at	6583	SLC22A4	Hs.310591	solute carrier family 22 (organic cation/er	-6.2032027	-1.3165069	1.3165064

202803_s_at	3689	ITGB2	Hs.375957	integrin, beta 2 (complement component 3	-6.2345276	-1.3201404	1.3201399
205100_at	9945	GFPT2	Hs.696497	glutamine-fructose-6-phosphate transamir	-6.273355	-1.3246188	1.3246183
210517_s_at	9590	AKAP12	Hs.371240	A kinase (PRKA) anchor protein 12	-6.3059344	-1.3283553	1.3283548
237056_at	387755	INSC	Hs.591997	inscuteable homolog (Drosophila)	-6.3810415	-1.336896	1.336896
215997_s_at	8450	CUL4B	Hs.102914	cullin 4B	-6.4188375	-1.341156	1.341156
207265_s_at	11015	KDELR3	Hs.720434	KDEL (Lys-Asp-Glu-Leu) endoplasmic re	-6.475102	-1.3474517	1.3474512
200982_s_at	309	ANXA6	Hs.412117	annexin A6	-6.4966655	-1.3498497	1.3498497
241418_at	344887	LOC344887	Hs.128803	Similar to hCG2041270	-6.547037	-1.3554211	1.3554211
211653_x_at	1646	AKR1C2	Hs.567256	aldo-keto reductase family 1, member C2	-6.558867	-1.3567238	1.3567228
215294_s_at	6594	SMARCA1	Hs.152292	SWI/SNF related, matrix associated, actin	-6.624789	-1.3639371	1.3639374
216594_x_at	1645	AKR1C1	Hs.460260	aldo-keto reductase family 1, member C1	-6.6767335	-1.3695707	1.3695717
202998_s_at	4017	LOXL2	Hs.626637	lysyl oxidase-like 2	-6.7066116	-1.3727918	1.3727922
227530_at	9590	AKAP12	Hs.371240	A kinase (PRKA) anchor protein 12	-6.731448	-1.3754582	1.3754587
219799_s_at	10170	DHRS9	Hs.179608	dehydrogenase/reductase (SDR family) m	-6.7386007	-1.3762245	1.3762245
210095_s_at	3486	IGFBP3	Hs.450230	insulin-like growth factor binding protein	-6.80172	-1.3829498	1.3829498
239461_at	117248	GALNTL2	Hs.411308	UDP-N-acetyl-alpha-D-galactosamine:po	-6.9264135	-1.3960543	1.3960543
204823_at	89795	NAV3	Hs.655301	neuron navigator 3	-6.969444	-1.4005218	1.4005218
201867_s_at	6907	TBL1X	Hs.495656	transducin (beta)-like 1X-linked	-7.033477	-1.4071188	1.4071193
201438_at	1293	COL6A3	Hs.233240	collagen, type VI, alpha 3	-7.076669	-1.4115353	1.4115353
212667_at	6678	SPARC	Hs.111779	secreted protein, acidic, cysteine-rich (ost	-7.097967	-1.413703	1.413703
209598_at	10687	PNMA2	Hs.591838	paraneoplastic antigen MA2	-7.1175213	-1.4156873	1.4156876
209613_s_at	125	ADH1B	Hs.4	alcohol dehydrogenase 1B (class I), beta p	-7.22031	-1.4260302	1.4260306
206707_x_at	9750	FAM65B	Hs.559459	family with sequence similarity 65, memb	-7.269605	-1.4309382	1.4309387
229893_at	257019	FRMD3	Hs.709357	FERM domain containing 3	-7.423244	-1.4460249	1.4460249
1553102_a_at	26112	CCDC69	Hs.655336	coiled-coil domain containing 69	-7.456529	-1.4492521	1.4492521
204017_at	11015	KDELR3	Hs.720434	KDEL (Lys-Asp-Glu-Leu) endoplasmic re	-7.5281696	-1.4561491	1.45615
217504_at	23460	ABCA6	Hs.709514	ATP-binding cassette, sub-family A (ABC	-7.579371	-1.4610391	1.4610391
202766_s_at	2200	FBN1	Hs.591133	fibrillin 1	-7.6396346	-1.4667516	1.466752
210762_s_at	10395	DLC1	Hs.134296	deleted in liver cancer 1	-7.696713	-1.4721212	1.4721212
242358_at			Hs.560092		-7.7202497	-1.4743237	1.4743237
207526_s_at	9173	IL1RL1	Hs.66	interleukin 1 receptor-like 1	-7.846513	-1.4860258	1.4860258
203603_s_at	9839	ZEB2	Hs.34871	zinc finger E-box binding homeobox 2	-7.867619	-1.4879634	1.4879637
242979_at	3667	IRS1	Hs.471508	insulin receptor substrate 1	-7.883585	-1.4894261	1.4894257
1568765_at	5054	SERPINE1	Hs.414795	serpin peptidase inhibitor, clade E (nexin,	-8.14106	-1.512608	1.5126085
202952_s_at	8038	ADAM12	Hs.594537	ADAM metallopeptidase domain 12	-8.386179	-1.5340066	1.5340071
232231_at	860	RUNX2	Hs.535845	runt-related transcription factor 2	-8.397454	-1.534976	1.534976
210869_s_at	4162	MCAM	Hs.599039	melanoma cell adhesion molecule	-8.403837	-1.5355239	1.5355244
230645_at	257019	FRMD3	Hs.709357	FERM domain containing 3	-8.603889	-1.5524945	1.5524945
211896_s_at	1634	DCN	Hs.156316	decorin	-8.606341	-1.5527	1.5527
211813_x_at	1634	DCN	Hs.156316	decorin	-8.7933655	-1.5682077	1.5682077
212233_at	4131	MAP1B	Hs.335079	microtubule-associated protein 1B	-8.832006	-1.5713706	1.5713706
205083_at	316	AOX1	Hs.406238	aldehyde oxidase 1	-8.8324795	-1.5714092	1.5714092
226777_at	8038	ADAM12	Hs.594537	ADAM metallopeptidase domain 12	-9.004985	-1.5853624	1.5853615

209087_x_at	4162	MCAM	Hs.599039	melanoma cell adhesion molecule	-9.087946	-1.5919771	1.5919771
225664_at	1303	COL12A1	Hs.101302	collagen, type XII, alpha 1	-9.143395	-1.596365	1.596365
235746_s_at					-9.323204	-1.6104128	1.6104131
1562321_at	5166	PDK4	Hs.8364	pyruvate dehydrogenase kinase, isozyme 4	-9.354758	-1.6128502	1.6128502
229014_at	441094	FLJ42709	Hs.457407	hypothetical LOC441094	-9.355024	-1.6128707	1.6128707
226237_at	1295	COL8A1	Hs.654548	collagen, type VIII, alpha 1	-9.435857	-1.6190767	1.6190767
221921_s_at	57863	CADM3	Hs.365689	cell adhesion molecule 3	-9.449251	-1.6201	1.6201
1563469_at			Hs.684023		-9.512776	-1.6249332	1.6249332
208335_s_at	2532	DARC	Hs.153381	Duffy blood group, chemokine receptor	-9.587801	-1.6306	1.6306
202627_s_at	5054	SERPINE1	Hs.414795	serpin peptidase inhibitor, clade E (nexin,	-9.616835	-1.632781	1.632781
210495_x_at	2335	FN1	Hs.203717	fibronectin 1	-9.652819	-1.6354752	1.6354752
203874_s_at	6594	SMARCA1	Hs.152292	SWI/SNF related, matrix associated, actin	-9.654708	-1.6356163	1.6356163
202663_at	7456	WIPF1	Hs.128067	WAS/WASL interacting protein family, m	-9.711383	-1.6398385	1.6398382
216442_x_at	2335	FN1	Hs.203717	fibronectin 1	-9.7997675	-1.6463737	1.6463737
209335_at	1634	DCN	Hs.156316	decorin	-9.812979	-1.6473455	1.6473455
203680_at	5577	PRKAR2B	Hs.433068	protein kinase, cAMP-dependent, regulato	-9.875665	-1.6519389	1.6519389
211340_s_at	4162	MCAM	Hs.599039	melanoma cell adhesion molecule	-10.009483	-1.6616478	1.6616478
205609_at	284	ANGPT1	Hs.369675	angiopoietin 1	-10.068479	-1.6658869	1.6658869
231849_at	144501	KRT80	Hs.140978	keratin 80	-10.070702	-1.6660461	1.6660461
212464_s_at	2335	FN1	Hs.203717	fibronectin 1	-10.154918	-1.6720533	1.6720533
211719_x_at	2335	FN1	Hs.203717	fibronectin 1	-10.211138	-1.6760359	1.6760359
213488_at	25992	SNED1	Hs.471834	sushi, nidogen and EGF-like domains 1	-10.222836	-1.6768618	1.6768618
220330_s_at	64092	SAMSN1	Hs.473341	SAM domain, SH3 domain and nuclear lo	-10.224559	-1.6769834	1.6769834
201893_x_at	1634	DCN	Hs.156316	decorin	-10.237321	-1.6778831	1.6778831
213790_at	8038	ADAM12	Hs.594537	ADAM metallopeptidase domain 12	-10.249472	-1.6787391	1.6787386
213425_at	7474	WNT5A	Hs.643085	wingless-type MMTV integration site fam	-10.346134	-1.6855102	1.6855097
212764_at	6935	ZEB1	Hs.124503	zinc finger E-box binding homeobox 1	-10.382867	-1.6880665	1.6880665
227646_at	1879	EBF1	Hs.573143	early B-cell factor 1	-10.463688	-1.6936598	1.6936598
206382_s_at	627	BDNF	Hs.502182	brain-derived neurotrophic factor	-10.963566	-1.7273226	1.7273226
206029_at	27063	ANKRD1	Hs.448589	ankyrin repeat domain 1 (cardiac muscle)	-11.203155	-1.7429166	1.7429166
226084_at	4131	MAP1B	Hs.335079	microtubule-associated protein 1B	-11.32302	-1.7505932	1.7505937
226751_at	25927	CNRIP1	Hs.212885	cannabinoid receptor interacting protein 1	-11.421011	-1.7568092	1.7568092
1562102_at	1645	AKR1C1	Hs.460260	Aldo-keto reductase family 1, member C1	-11.49226	-1.7612953	1.7612953
205990_s_at	7474	WNT5A	Hs.643085	wingless-type MMTV integration site fam	-11.978398	-1.7911816	1.7911816
205207_at	3569	IL6	Hs.654458	interleukin 6 (interferon, beta 2)	-12.139157	-1.8007979	1.8007984
229978_at	729993	SHISA9	Hs.724830	shisa homolog 9 (Xenopus laevis)	-12.320913	-1.8115187	1.8115187
214587_at	1295	COL8A1	Hs.654548	collagen, type VIII, alpha 1	-12.414322	-1.8169665	1.816967
202628_s_at	5054	SERPINE1	Hs.414795	serpin peptidase inhibitor, clade E (nexin,	-12.426839	-1.8176937	1.8176937
224009_x_at	10170	DHRS9	Hs.179608	dehydrogenase/reductase (SDR family) m	-12.807432	-1.8394547	1.8394547
202664_at	7456	WIPF1	Hs.128067	WAS/WASL interacting protein family, m	-13.659154	-1.8858981	1.8858981
209909_s_at	7042	TGFB2	Hs.133379	transforming growth factor, beta 2	-13.815274	-1.8940959	1.8940964
205608_s_at	284	ANGPT1	Hs.369675	angiopoietin 1	-13.845522	-1.8956738	1.8956738
229461_x_at	257194	NEGR1	Hs.146542	neuronal growth regulator 1	-14.867603	-1.9470501	1.9470501

1556200_a_at	118611	C10orf90	Hs.587663	chromosome 10 open reading frame 90	-14.975254	-1.9522543	1.9522543
223952_x_at	10170	DHRS9	Hs.179608	dehydrogenase/reductase (SDR family) m	-15.2023535	-1.9631114	1.9631114
228121_at	7042	TGFB2	Hs.133379	transforming growth factor, beta 2	-16.578558	-2.0256233	2.0256233
244503_at			Hs.671876		-16.638216	-2.0282145	2.0282145
237435_at			Hs.61596		-17.168245	-2.0508351	2.0508356
210222_s_at	6252	RTN1	Hs.368626	reticulon 1	-17.240131	-2.0538497	2.0538492
201981_at	5069	PAPPA	Hs.643599	pregnancy-associated plasma protein A, p	-17.321596	-2.05725	2.05725
224942_at	5069	PAPPA	Hs.643599	pregnancy-associated plasma protein A, p	-17.477352	-2.0637074	2.0637074
212190_at	5270	SERPINE2	Hs.38449	serpin peptidase inhibitor, clade E (nexin,	-17.635206	-2.0701933	2.0701933
224940_s_at	5069	PAPPA	Hs.643599	pregnancy-associated plasma protein A, p	-17.97539	-2.0839753	2.0839758
209829_at	9750	FAM65B	Hs.559459	family with sequence similarity 65, memb	-18.039228	-2.086533	2.0865326
210809_s_at	10631	POSTN	Hs.136348	periostin, osteoblast specific factor	-18.078676	-2.0881085	2.0881085
203485_at	6252	RTN1	Hs.368626	reticulon 1	-18.144512	-2.0907307	2.0907307
228176_at	1903	S1PR3		sphingosine-1-phosphate receptor 3	-19.479961	-2.1419597	2.1419592
209505_at	7025	NR2F1	Hs.519445	nuclear receptor subfamily 2, group F, me	-19.508059	-2.1429992	2.1429992
218723_s_at	28984	C13orf15	Hs.507866	chromosome 13 open reading frame 15	-19.83042	-2.1548219	2.1548214
228128_x_at	5069	PAPPA	Hs.643599	pregnancy-associated plasma protein A, p	-19.9968	-2.1608486	2.1608486
236361_at	117248	GALNTL2	Hs.411308	UDP-N-acetyl-alpha-D-galactosamine:po	-20.140778	-2.1660237	2.1660237
224941_at	5069	PAPPA	Hs.643599	pregnancy-associated plasma protein A, p	-20.44172	-2.176722	2.1767225
226771_at	57198	ATP8B2	Hs.435700	ATPase, class I, type 8B, member 2	-23.971252	-2.291617	2.2916164
226279_at	11098	PRSS23	Hs.25338	protease, serine, 23	-24.393763	-2.3042202	2.3042202
217626_at	1645	AKR1C1	Hs.460260	aldo-keto reductase family 1, member C1	-24.584946	-2.3098516	2.3098516
228501_at	117248	GALNTL2	Hs.411308	UDP-N-acetyl-alpha-D-galactosamine:po	-24.93398	-2.3200207	2.3200207
235743_at					-25.423904	-2.3340569	2.3340569
213400_s_at	6907	TBL1X	Hs.495656	transducin (beta)-like 1X-linked	-25.943924	-2.3486624	2.3486624
213948_x_at	57863	CADM3	Hs.365689	cell adhesion molecule 3	-28.2643	-2.4104543	2.4104548
1555854_at	1645 /// 1646	AKR1C1 /// AK	Hs.567256	aldo-keto reductase family 1, member C1	-28.763153	-2.4230752	2.4230747
202458_at	11098	PRSS23	Hs.25338	protease, serine, 23	-30.866488	-2.4739847	2.4739847
220014_at	51334	PRR16	Hs.157461	proline rich 16	-40.126747	-2.663246	2.6632462
201859_at	5552	SRGN	Hs.1908	serglycin	-47.258938	-2.7812576	2.7812576
201858_s_at	5552	SRGN	Hs.1908	serglycin	-70.43599	-3.0691204	3.0691204

Supplementary Table 2. Antibodies used in this study

Antibody	Supplier	Catalogue number	Applications in this study
EGFR (D38B1)	Cell Signaling Technology	4267	Western blot (WB) (1: 1000 dilution)
EGFR (EGFR.1)	Thermo Scientific	MS-331	Immunofluorescence (IF) (1: 100 dilution), Immunoprecipitation (1: 100 dilution)
EGFR (14C10)	in-house-prepared	-	Neutralization (Neu) (10 ug/mL)
Phospho-EGFR Tyr1173	Epitomics	1124	WB (1: 1000 dilution)
Phospho-EGFR Tyr845	Epitomics	2342	WB (1: 1000 dilution)
Phospho-EGFR Tyr1045	Cell Signaling Technology	2237	WB (1: 1000 dilution)
Phospho-EGFR Tyr1068 (D7A5)	Cell Signaling Technology	3777	WB (1: 1000 dilution)
Phospho-EGFR Tyr1086	Epitomics	1139	WB (1: 1000 dilution)
HER2/ErbB2 (D8F12)	Cell Signaling Technology	4290	WB (1: 1000 dilution)
MEK1 (61B12)	Cell Signaling Technology	2352	WB (1: 1000 dilution)
ERK1/2 (137F5)	Cell Signaling Technology	4695	WB (1: 1000 dilution)
Phospho-ERK1/2 Thr202/pTyr204 (D13.14.4E)	Cell Signaling Technology	4370	WB (1: 1000 dilution)
FRA1 (N-17)	Santa Cruz Biotechnology	sc-183	WB (1: 1000 dilution)
Phospho-FRA1 Ser265 (D22B1)	Cell Signaling Technology	5841	WB (1: 1000 dilution)
Akt	Cell Signaling Technology	9272	WB (1: 1000 dilution)
Phospho-Akt Ser473	Cell Signaling Technology	9271	WB (1: 1000 dilution)
RSK1 (D6D5)	Cell Signaling Technology	8408	WB (1: 1000 dilution)
RSK2 (D21B2)	Cell Signaling Technology	5528	WB (1: 1000 dilution)
Ubiquitin (P4D1)	Cell Signaling Technology	3936	WB (1: 1000 dilution)
ZEB1 (D80D3)	Cell Signaling Technology	3396	WB (1: 500 dilution)
Snail (C15D3)	Cell Signaling Technology	3879	WB (1: 1000 dilution)
Slug (C19G7)	Cell Signaling Technology	9585	WB (1: 1000 dilution)
E-cadherin (24E10)	Cell Signaling Technology	3195	WB (1: 1000 dilution), IF (1: 200 dilution)
E-cadherin (SHE78-7)	TaKaRa	M126	Neu (4 ug/mL)
N-cadherin	Cell Signaling Technology	4061	WB (1: 1000 dilution)
Vimentin (D21H3)	Cell Signaling Technology	5741	WB (1: 1000 dilution), IF (1: 200 dilution)
Fibronectin (10/Fibronectin)	BD Bioscience	610077	WB (1: 1000 dilution)
Occuludin	Invitrogen	71-1500	WB (1: 1000 dilution)
Twist (Twist2C1a)	Abcam	ab50887	WB (1: 1000 dilution)
Claudin-1	Cell Signaling Technology	4933	WB (1: 1000 dilution)

beta-Actin (AC-15)	Sigma-Aldrich	A5441	WB (1: 5000 dilution)
beta-Tubulin (JDR.3B8)	Sigma-Aldrich	T8535	WB (1: 5000 dilution)
PE anti-human EGFR (EGFR1)	Abcam	ab130738	Flow Cytometry (FC) (2 uL/2E+05 cells)
PE mouse IgG2b, k isotype Ctrl	BioLegend	400314	FC (2 uL/2E+05 cells)
FITC anti-human CD44 (G44-26)	BD Pharmingen	560977	FC (2 uL/2E+05 cells)
FITC mouse IgG2b, k isotype Ctrl	BioLegend	400309	FC (2 uL/2E+05 cells)
APC anti-human CD24 (ML5)	BioLegend	311117	FC (2 uL/2E+05 cells)
APC mouse IgG2a, k isotype Ctrl	BioLegend	400219	FC (2 uL/2E+05 cells)
APC anti-human CD326 (EpCAM) (9C4)	BioLegend	324207	FC (4 uL/2E+05 cells)
APC mouse IgG2b, k isotype Ctrl	BioLegend	400319	FC (0.6 uL/2E+05 cells)
FITC anti-human CD49f (GoH3)	BD Pharmingen	555735	FC (2 uL/2E+05 cells)
FITC Rat IgG2a, k isotype Ctrl	BD Pharmingen	555843	FC (2 uL/2E+05 cells)

Supplementary Table 3. Primers used in this study

	Gene	Forward	Reverse
RT-qPCR	EGFR	GTCTGCCATGCCTTGTGCTC	CTTGTCCACGCATTCCCTGC
	ErbB2	AGCCTTGCCCCATCAACTG	AATGCCAACCACCGCAGA
	ErbB3	CCCTGCCATGAGAACTGCAC	TCACTGTCAAAGCCATTGTCAGAT
	ErbB4	CCTGGAAGAAAGACGACTCGTTC	CGTCACTCTGATGGGTGAATTTCC
	E-cadherin	TGCCCAGAAAATGAAAAAGG	GTGTATGTGGCAATGCGTTC
	N-cadherin	ACAGTGGCCACCTACAAAGG	CCGAGATGGGGTTGATAATG
	ZEB1	GGAAAGGAAGGGCAAGAAATCCTGG	TGGTGTGCCCTGCCTCTGGT
	ZEB2	CAAGAGGCGCAAACAAGC	GGTTGGCAATACCGTCATCC
	Snail	CCTCCCTGTCAGATGAGGAC	CCAGGCTGAGGTATTCCTTG
	Slug	GGGGAGAAGCCTTTTTCTTG	TCCTCATGTTTGTGCAGGAG
	Twist1	GGAGTCCGCAGTCTTACGAG	TCTGGAGGACCTGGTAGAGG
	Vimentin	GAGAACTTTGCCGTTGAAGC	GCTTCCTGTAGGTGGCAATC
	Fibronectin 1	CAGTGGGAGACCTCGAGAAG	TCCCTCGGAACATCAGAAAC
	TGF-beta1	TGTCACCGGAGTTGTGCGGC	GCAGTGGGCGCTAAGGCGAA
	TGF-beta2	ACCAACCGGCGGAAGAA	CACCCTAGATCCCTCTTGAAATCA
	TGF-beta3	AGGCCCTTGCCCATACCT	AGATGCTTCAGGGTTCAGAGTGTT
	GAPDH	CATGAGAAGTATGACAACAGCCT	AGTCCTTCCACGATACCAAAGT
Construction	wt MEK1 cDNA cloning	TTActcgaggccaccATGCCCAAGAAGAAGCCGAC	TTAggatccTTAGACGCCAGCAGCATGGG
	CA-MEK1 mutagenesis	GAATTCGTGGGCACAAGGTCCTA	GTTGGCCATGTCGTCGATGAGCTG
	DN-MEK1 mutagenesis	GCCTTCGTGGGCACAAGGTCCTA	GTTGGCCATGGCGTCGATGAGCT
	ZEB1 cDNA cloning	ATACTCGAGATGGCGGATGGCCCCAGGTG	GGCTCTAGATTAGGCTTCATTTGTCTTTTCTTCAGACACTTGCTC

Supplementary Table 4. siRNAs used in this study

Gene	Supplier	Catalogue number	Sequences (sense strand)	Descriptions/References
ErbB2	Dharamacon	L-003126-00-0005	-	ON-TARGETplus siRNA - SMARTpool
ErbB3	Dharamacon	L-003127-00-0005	-	ON-TARGETplus siRNA - SMARTpool
MEK1	Sigma-Aldrich	SASI_Hs01_00090167	-	MISSION siRNA
ERK1	Sigma-Aldrich	Custom Products	5'-CGUCUAAUAUAUAAAUAUAdTdT-3'	Anjum et al, Curr Biol. 2005;15(19):1762-7.
ERK2	Sigma-Aldrich	Custom Products	5'-CAUGGUAGUCACUAACAUAdTdT-3'	Anjum et al, Curr Biol. 2005;15(19):1762-7.
RSK1	Sigma-Aldrich	Custom Products	5'-CCCAACAUCAUCACUCUGAAAdTdT-3'	Anjum et al, Curr Biol. 2005;15(19):1762-7.
RSK2	Sigma-Aldrich	Custom Products	5'-AGCGCUGAGAAUGGACAGCAAdTdT-3'	Anjum et al, Curr Biol. 2005;15(19):1762-7.
FRA1	Sigma-Aldrich	Custom Products	5'-CACCAUGAGUGGCAGUCAGdTdT-3'	Vial and Marshall, J Cell Sci. 2003;116(Pt 24):4957-63.
ZEB1	Sigma-Aldrich	Custom Products	5'-UGAUCAGCCUCAAUCUGCAdTdT-3'	Eger et al, Oncogene. 2005;24(14):2375-85.

Supplementary Table 5. Synthetic microRNA inhibitors used in this study

microRNA	Supplier	Catalogue number	Descriptions
miR-200c	Life Technologies	ID:MH11714	mirVana miRNA inhibitor
miR-205	Life Technologies	ID:MH11015	mirVana miRNA inhibitor
